# Chronic Ethanol Exposure Induces Early Epithelial-to-Mesenchymal Transition (EMT) and Premalignant Changes in Gingival Keratinocytes: An In Vitro Model of Very Early Oral Carcinogenesis

**DOI:** 10.3390/cells14231887

**Published:** 2025-11-27

**Authors:** Martin Philipp Dieterle, Thorsten Steinberg, Ayman Husari, Pascal Tomakidi

**Affiliations:** 1 Division of Oral Biotechnology, Center for Dental Medicine, Medical Center—University of Freiburg, Faculty of Medicine, University of Freiburg, Hugstetterstr. 55, 79106 Freiburg, Germany; martin.dieterle@uniklinik-freiburg.de (M.P.D.); pascal.tomakidi@uniklinik-freiburg.de (P.T.); 2 Department of Orthodontics, Center for Dental Medicine, Medical Center—University of Freiburg, Faculty of Medicine, University of Freiburg, Hugstetterstr. 55, 79106 Freiburg, Germany; ayman.husari@uniklinik-freiburg.de

**Keywords:** squamous cell carcinoma of head and neck, carcinogenesis, ethanol, human papillomavirus, epithelial–mesenchymal transition, Cadherins, Vimentin

## Abstract

**Highlights:**

**What are the main findings?**

- Chronic ethanol exposure of gingival keratinocytes induces biochemical and morphological changes that resemble the very early steps in the development of oral squamous cell carcinoma.
- Even morphologically “normal” keratinocytes already show early, premalignant metabolic changes, which underscores the concept of a stepwise cell transformation from a benign to a premalignant phenotype.

**What are the implications of the main findings?**

- The multistep process of ethanol-induced gingival keratinocyte transformation can be modelled in an animal-free cell culture system.
- Strategies for the early diagnosis and treatment of ethanol-induced human oral squamous cell carcinoma should consider both metabolic and proteinaceous changes in gingival keratinocytes.

**Abstract:**

Early molecular events underlying ethanol-induced oral squamous cell carcinoma development remain insufficiently understood, primarily due to a lack of suitable in vitro systems that recapitulate the initial stages of premalignant transformation. Therefore, a cell culture model of human gingival keratinocytes representing progressive stages of early ethanol-induced cell transformation was established and comprehensively characterized. The three cell lines, named “gingival keratinocytes” (GK), “epithelioid” (EPI) and “fibroblastoid” (FIB), and their derivatives were analyzed by morphological, cell biological and biochemical methods, with an emphasis on epithelial-to-mesenchymal transition (EMT)-related signaling pathways. All cell lines were non-tumorigenic in vitro. Chronic ethanol exposure induced distinct morphological and molecular alterations that capture early premalignant changes in vitro. This includes reduced *E-Cadherin* and enhanced *Vimentin* expression, accompanied by an increased production of reactive oxygen species. Notably, even morphologically stable cell lines displayed metabolic susceptibility to EMT induction, indicating the early activation of transformation-associated signaling cascades even in a premalignant state. These alterations, however, closely mirrored pathohistological features of oral squamous cell carcinomas such as loss of epithelial integrity and acquisition of mesenchymal characteristics. Collectively, the presented model provides a robust and accessible in vitro platform for investigating very early ethanol-induced oral carcinogenesis mechanisms that are relevant in a premalignant state and may facilitate the identification of diagnostic and preventive biomarkers to improve patient outcomes in alcohol-associated oral cancer and precursor lesions.

## 1. Introduction

Oral squamous cell carcinomas (OSCCs) represent the most frequent malignancy in the oral cavity and constitute a major medical and socioeconomic burden. In 2022, approximately 390,000 people were newly diagnosed with, and 190,000 people died from, cancers of the lip and oral cavity, according to data from the Global Cancer Observatory. Although advances in surgery and adjuvant therapy have improved local control, the five-year survival rates of OSCCs have not significantly changed for decades, mainly due to the lack of early diagnostic markers. Understanding the early molecular events that precede overt malignancy is therefore critical for developing innovative preventive and diagnostic approaches [1,2,3,4].

The main exogenous drivers of OSCC development are exposure to tobacco products and the consumption of alcohol-containing beverages [5,6,7]. The carcinogenic potential of alcoholic beverages was acknowledged by the International Agency for Research on Cancer (IACR) as early as 1988 [8]. Ethanol (EtOH) and its primary metabolite, acetaldehyde (AA), are considered the main carcinogenic agents acting on the oral cavity [9]. From a clinical point of view, low to moderate alcohol consumption already leads to an increased risk of developing cancer and acts synergistically with tobacco use [10,11]. EtOH and AA toxicity in the oral cavity are based on multiple mechanisms, including DNA adduct formation, the inactivation of DNA-repairing enzymes and epigenetic changes [12,13,14,15]. Intracellularly, EtOH is metabolized to AA by alcohol dehydrogenases (ADH) and subsequently to acetic acid by aldehyde dehydrogenases (ALDH). Genetic polymorphisms of ADH and ALDH substantially influence reaction kinetics and contribute to individual OSCC susceptibility, especially in the Asian population, where ALDH variants with reduced function are common [16,17,18,19]. Additionally, larger amounts of EtOH can be metabolized by the microsomal ethanol oxidizing system (MEOS) and its cytochrome-P450-Oxidases (subtype 2E1), which produce reactive oxygen species (ROS) that promote oxidative stress and lipid peroxidation [20,21]. Moreover, bacterial metabolism in the oral cavity also contributes to high AA concentrations in saliva due to the low turnover of AA to acetic acid by oral bacteria [22,23]. While these mechanisms are conceptually well-understood, the precise molecular events driving the very early transformation of oral epithelial cells undergoing chronic EtOH exposure remain poorly defined.

Investigations into early OSCC development are hindered by the lack of suitable in vitro models. Primary explant cultures are very often bacterially contaminated, and therefore, many commercially available cell lines are derived from OSCC metastases [24]. The latter thus represent late, genetically complex tumor stages. Tissue-engineering or organoid-based platforms trying to simulate the multistep in vitro tumorigenesis of OSCCs mainly rely on OSCC-derived cell lines and, consequently, also fail to capture the initial transformation process [25,26,27,28,29]. Only very few model systems have tried to systematically induce cell transformation in vitro in gingival keratinocytes by continuous or repeated exposure to defined carcinogens. Concerning EtOH-induced OSCC development, there is, to the best of our knowledge, only one established model system, which was the basis for this experimental study [30]. The model system comprises three morphologically stable cell lines: a human papillomavirus type 16 (HPV16) E6/E7-immortalized gingival keratinocyte cell line, designated as GK, and two derivatives of GK, which are designated as epithelioid (EPI) and fibroblastoid (FIB). Both EPI and FIB were derived from GK by chronic in vitro EtOH exposure. Based on previous experimental findings, EPI and FIB represent progressive cellular transformation stages, since the molecular and phenotypic changes observed in EPI and FIB cells are consistent with the first hallmarks of epithelial-to-mesenchymal transition (EMT) [31]. Of note, both cell types remain non-tumorigenic in the *nude*-mouse model, express HPV-16 E6/E7, which proves their origin, and exhibit molecular marker profiles that clearly distinguish them from human gingival fibroblasts [30,31,32,33,34,35].

EMT involves complex modulation of mechanobiological signaling pathways at adherens junctions (AJs), focal adhesions (FAs) and the Hippo signaling axis [36,37]. Although numerous studies have described the individual role of these signaling pathways in OSCCs, their concerted deregulation in response to chronic EtOH exposure has not been systematically studied. Therefore, it remains unclear how EtOH-driven genetic and biochemical alterations, as well as oxidative stress, translate into mechanobiological reprogramming in oral keratinocytes in the context of premalignant transformation.

To this end, the present study aimed at (i) further developing and characterizing the GK/EPI/FIB cell culture model as an in vitro system of early EtOH-induced transformation in human gingival keratinocytes; (ii) defining EtOH-associated changes in AJs, FAs, Hippo signaling, cytoskeletal organization and EMT markers, and interpreting these findings in the context of premalignant cell transformation; and (iii) assessing the susceptibility of these differentially transformed cell lines to further experimental induction of EMT.

By integrating morphological, cell biological and biochemical analyses, this work provides novel insights into the very early events of EtOH-induced oral carcinogenesis and offers new perspectives for future clinical secondary prevention through a molecular biomarker-based detection of premalignant lesions in the oral cavity.

## 2. Materials and Methods

### 2.1. Cell Culture

Primary human gingival keratinocytes were isolated from healthy gingival tissue with written informed consent of the donor and approval by the Ethics Committee of Heidelberg University (Approval ID: 148/2003, date 30 September 2005) and in accordance with the principles outlined in the Declaration of Helsinki (1975, revised in 2013). These primary cells were immortalized by introducing the human papillomavirus type 16 (HPV16) E6/E7 genes, giving rise to the gingival keratinocyte cell line (GK) originally established by our research team at the University of Freiburg. The parental GK cell line used in this study corresponds to the HPV16 E6/E7–immortalized human oral gingival keratinocyte line available from Applied Biological Materials Inc. (ABM, Richmond, BC, Canada; catalog no. GM-T0717). Two GK-derived sublines, termed EPI and FIB, were established by our group through chronic ethanol exposure of GK cells in 2003, as described in [30]. Back then, immortalized GK cells at passages 13–19 were grown in standard cell culture flasks in supplemented KGM-2 medium in a standard cell culture incubator with a humidified atmosphere containing 60 mM of EtOH (replaced once weekly). From passage 20 onwards, 60 mM of EtOH was also added to the culture medium directly. The culture medium was exchanged twice weekly; the measured steady-state EtOH concentration in the medium was 30 mM. After 9 weeks, the medium was changed to DMEM/FCS to obtain differentiation-resistant cell populations, i.e., cells not undergoing terminal differentiation in the presence of high calcium concentrations. After two weeks without trypsinization, differentiation-resistant cells were observed. The latter were transferred to new cultures (corresponding to a new passage 1 of EtOH-treated cells). Parallel control cultures without EtOH exposure did not survive the switch to DMEM/FCS. After 5–6 months, at passages 20–25, two distinct morphological subpopulations appeared, which were separated by differential trypsinization (and not by single-cell cloning) and were designated as EPI and FIB. Notably, both sublines derive from the same treated GK culture and, therefore, are not clonal isolates, but rather enriched subpopulations representing epithelial-like vs. mesenchymal-like phenotypic states of the originally EtOH-exposed cells. Consistent with the original report, the FIB cells exhibited more pronounced mesenchymal features (e.g., spindle shape and later anchorage-independent growth) relative to EPI, reflecting a further advanced stage of premalignant transformation in this in vitro model.

For current experiments, standard cell culture was performed according to the repeatedly published internal laboratory standard at 37 °C and 5% CO_2_ [34,38]. At a confluency of 80–90%, cells were trypsinized (Trypsin 0.05% [*w*/*v*], Ethylenediaminetetraacetic acid 0.02% [*w*/*v*]; Anprotec, Bruckberg, Germany) in phosphate-buffered saline (PBS; Life Technologies GmbH, Darmstadt, Germany) at 37 °C for 5–10 min. After centrifugation at 259× *g*, cells were resuspended in the corresponding medium, counted and seeded according to the experimental needs. The GK, EPI and FIB cell lines, as well as their derivatives, were used in the passages described (see Supplementary Table S.M1). The medium was exchanged completely every two to four days. Before all experiments and regularly during the experimental work, the cells were tested for mycoplasma contamination with a MycoStrip™ (InvivoGen, San Diego, CA, USA) detection kit according to the manufacturer’s protocol.

### 2.2. EtOH Treatment of Cell Lines

To enable the continuous EtOH (Fluka Chemie GmbH, Buchs, Switzerland) exposure of cell lines under the conditions of a standard cell culture laboratory, a new method of EtOH treatment in a bicarbonate-free buffer system with closed cell culture flasks was developed [39]. Closed cell culture flasks were used to ensure stable vapor pressure and, therefore, a constant EtOH concentration in the cell culture medium. Preliminary experiments in our laboratory showed that GK cells could be cultured in KGM-2 medium in closed cell culture flasks for at least 48 h without changing the medium and without showing morphological or functional changes. Therefore, EtOH application was directly possible in these cells (see Supplementary Table S.M1). For EPI and FIB cells, Leibovitz L15 Medium (Thermo Fisher Scientific, Waltham, MA, USA), which was especially developed to be used in CO_2_-free, bicarbonate-independent conditions, was used [40]. A final EtOH concentration of 86 mM, corresponding to 0.5% [*v*/*v*]/5‰ [*v*/*v*] at 20 °C, was used, as it was not directly cytotoxic but led to morphological and cell biological changes within a reasonable timespan. Preliminary vaporization experiments proved that standard cell culture flasks (Greiner Bio-One GmbH, Frickenhausen, Germany) were technically impermeable to EtOH. A total fluid volume of 6 mL was used in 25 cm^2^ culture flasks to (i) reduce the volume of the gas phase, i.e., optimizing the surface/volume ratio of the cell culture, and to (ii) enable a sufficient nutrient supply to the cells for the periods between medium changes (every 24–48 h).

To assess the plausibility of this approach, we conducted a simple physicochemical modeling of this culture system (see Supplementary Material and Methods and [41]).

With this theoretical background, GK cells grew in fully supplemented KGM-2 medium with 0.5% [*v*/*v*] EtOH in closed cell culture flasks for two months at 37 °C. Similarly, EPI and FIB cells grew in L15 medium with 10% [*v*/*v*] fetal calf serum (FCS), 0.1 mg/mL Kanamycin and 0.5% EtOH [*v*/*v*] in closed cell culture flasks for two months at 37 °C. The medium was exchanged completely every 24 to 48 h. As control conditions, the same cell lines grew in closed cell culture flasks for two months at 37 °C without the addition of EtOH (designated as “EtOH Ko” cell lines, see Supplementary Table S.M1).

After two months, EtOH-treated EPI and FIB cells showed remarkable morphological changes as detected via light microscopy. Morphologically different subclones were isolated using differential trypsinization [30,42]. The resulting cell lines are designated as “EtOH” in this article. Adaptation to standard cell culture conditions was performed stepwise by replacing increasing amounts of L15 medium with DMEM medium. Subsequent cell culture was performed as described in Section 2.1. Taken together, nine different, morphologically stable cell lines, as shown in Supplementary Table S.M1, were used in this study.

### 2.3. Experimental Induction of Epithelial-to-Mesenchymal Transition (EMT)

To stably induce EMT in the aforementioned cell lines, the StemXVivo^®^ EMT Inducing Media Supplement (100×) of R&D Systems (Minneapolis, MI, USA) was used [43]. According to the manufacturer’s instructions, the EMT cocktail was diluted 1:100 in the respective cell culture medium, and cells were dissolved in the EMT cocktail-containing medium (“+EMT”) before seeding and treated for the timespan as indicated for the respective experiment. If needed, the medium was exchanged after 72 h and replaced by EMT cocktail-containing medium again. “−EMT” describes the negative control experimental setup, where no EMT cocktail was added.

### 2.4. Quantitative Polymerase Chain Reaction (qPCR)

Cell lines were cultivated to sub-confluence for a total of six days (if indicated, in the presence of the EMT cocktail). RNA extraction and cDNA synthesis were performed according to the previously published internal laboratory standard using the RNeasy^®^ Plus Mini Kit (Qiagen, Hilden, Germany) and the RevertAid First Strand cDNA Synthesis Kit (Thermo Fisher Scientific, Waltham, MA, USA) [38]. In brief, cells were washed in PBS three times and incubated with lysis buffer (RLT-buffer by Qiagen, Hilden, Germany, containing a final concentration of 4 mM Dithiothreitol [DTT; Sigma-Aldrich, St. Louis, MI, USA]) for 5 min at 4 °C in a shaking incubator before RNA extraction. Subsequently, the lysate was homogenized via pipetting and centrifuged for 2 min at 19,090× *g* through a QIAshredder chromatography column (Qiagen, Hilden, Germany). Next, samples were treated according to the manufacturer’s protocol of the RNeasy^®^ Plus Mni Kit (Qiagen, Hilden, Germany). The RNA concentration was determined using the microfluidic QIAxpert^®^ UV/VIS-spectrometer (Qiagen, Hilden, Germany). The mean value of two technical replicates was used for further calculations. All samples were diluted to a final concentration of 100 ng/µL with nuclease-free water (Qiagen, Hilden, Germany) and stored at −80 °C until further use. cDNA was synthesized using the RevertAid First Strand cDNA Synthesis Kit (Thermo Fisher Scientific, Waltham, MA, USA) according to the manufacturer’s protocol. A total of 10 µL of Mastermix (containing deoxyribonucleotides, Oligo-dT-Primers, random primers, RNAse-inhibitors and reverse transcriptase) was mixed with 1 µg of RNA and amplified using the CFX96™ thermocycler (Bio-Rad Laboratories GmbH, Basel, Switzerland). After equilibration for 5 min at 25 °C, cDNA synthesis was performed at 42 °C for 1 h. Subsequently, the cDNA-RNA hybrid double strands were denatured at 70 °C for 5 min. cDNA samples were stored at −20 °C until further use. For all samples a comparable efficiency of reverse transcription was assumed; i.e., no further measurements of nucleic acid concentration were performed.

SYBR^®^ Green I-based qPCR was performed using the qPCR Master Mix (Qiagen) and the corresponding, pre-validated RT^2^ qPCR Primer Assays (Qiagen; see Supplementary Table S.M2). According to the manufacturer’s instructions, 0.2 µL of cDNA was mixed with 1 µL of the primer of interest, 12.5 µL of SYBR^®^ Green Mastermix and 10.5 µL of nuclease-free water (Qiagen) in a 96-well plate (Greiner Bio-One GmbH, Frickenhausen, Germany). After the activation of the “hot start”-DNA Taq-Polymerase (10 min, 95 °C), DNA amplification was performed in 40 cycles of denaturation (15 s, 95 °C) and synthesis (1 min, 60 °C) in the CFX96™ thermocycler. A “no template control [NTC]” (specific primer without addition of cDNA) as well as a “no reverse transcript control [NRT]” (containing an RNA template and a Glyceraldehyde 3-phosphate dehydrogenase [GAPDH]-specific primer but no cDNA) were used as negative controls.

Data evaluation was based on cycle threshold (Ct) values. For each gene of interest (GOI), the arithmetic mean Ct value of two technical replicates was calculated (Ct GOI). The geometric mean Ct value of the arithmetic mean of two technical replicates of four housekeeping genes, namely *β-Tubulin (TBCB)*, *β-Actin (ACTB)*, *60S ribosomal protein L13a (RPL13A)* and *Glyceraldehyde 3-phosphate dehydrogenase (GAPDH)*, was calculated as well (Ct reference). The Ct reference was subtracted from Ct GOI for each biological replicate (ΔCt-values, n = 3 biological replicates). Further data evaluation was performed according to Section 2.11.

### 2.5. Gel Electrophoresis and Western Blot

Gel electrophoresis and Western Blot analyses were conducted according to the repeatedly published internal laboratory standard [34]. In brief, the respective cell lines were grown to sub-confluence for six days (if indicated, in the presence of the EMT cocktail). Whole cell protein lysates were produced using RIPA lysis buffer (Sigma-Aldrich) containing protease inhibitors (cOmplete™ Protease Inhibitor Cocktail, Roche Holding, Basel, Switzerland; 1 pill per 10 mL of RIPA lysis buffer) and phosphatase inhibitors (PhosSTOP™, Roche Holding; 1 pill per 10 mL of RIPA lysis buffer) for 5 min at room temperature (RT) in a shaking incubator. The lysates were centrifuged at 9838× *g* for 10 min at 4 °C. The supernatant was transferred to fresh reaction vials. Whole cell protein was quantified colometrically in triplicate using the Pierce™ BCA Protein Assay Kit (Life Technologies GmbH, Darmstadt, Germany) according to the manufacturer’s protocol (incubation for 30 min at 37 °C; colorimetric measurement at λ = 562 mm in an Infinite^®^ M200 microplate reader [Tecan Group AG, Männedorf, Switzerland]). The Pre-Diluted Protein Assay Standards: Bovine Serum Albumin (BSA) Set (Thermo Fisher Scientific) was used as a reference. Protein samples were diluted to 500 µg/mL for further use.

For Sodium dodecyl sulfate-polyacrylamide gel electrophoresis (SDS-PAGE), samples were mixed with 4× Laemmli buffer (Bio-Rad Laboratories GmbH) containing DTT in a final concentration of 4 mM, resulting in a final protein concentration of 375 µg/mL. Then, samples were denatured at 95 °C for 5 min, centrifuged at 9838× *g*, and the supernatant was transferred to a new reaction vial. Criterion™ TGX Stain-Free™ Precast gels (4–15%; Bio-Rad Laboratories GmbH, Basel, Switzerland), Tris/Glycin/SDS-running buffer (Bio-Rad Laboratories GmbH) and Precision Plus Protein™ All Blue Prestained Protein Standards (Bio-Rad Laboratories GmbH) were used for electrophoresis. In each lane, 10 µg of total protein was loaded, and the gels were run for 20 min at 90 V until samples reached the separation gel and again for 40 min at 180 V.

The gels were incubated in Trans-Blot Turbo 5x Transfer Buffer (Bio-Rad Laboratories GmbH) and subsequently placed on Polyvinylidene fluoride (PVDF) membranes (Bio-Rad Laboratories GmbH), which were activated in methanol (Sigma-Aldrich) before use. Blotting was performed with the help of the TransBlot^®^ TurboTM transfer system (Bio-Rad Laboratories GmbH) for 7 min at 25 V and 2.5 A. Total lane protein was quantified using the ChemiDoc™-Touch Imaging Systems (Bio-Rad Laboratories GmbH). Next, membranes were incubated in 5% [*w*/*v*] BSA (Sigma-Aldrich) in Tris(hydroxymethyl)aminomethane (TRIS)-buffered saline (TBS; Bio-Rad Laboratories GmbH) for 2 h at RT. Incubation with the respective primary antibody was performed overnight at 4 °C in a shaking incubator (see Supplementary Table S.M3). Membranes were subsequently washed three times in TBS buffer containing 0.05% [*v*/*v*] Tween^®^ 20 (Bio-Rad Laboratories GmbH; = TBST buffer). Afterwards, the samples were incubated with a horseradish peroxidase-coupled secondary antibody for 1 h at RT in a shaking incubator (see Supplementary Table S.M3). After two subsequent washing steps in TBS buffer (10 min at RT), membranes were incubated in the Clarity™ Western enhanced chemiluminescence (ECL) Substrate solution (Bio-Rad Laboratories GmbH) for 5 min at RT in the dark. Chemiluminescence intensity was detected with the ChemiDoc™-Touch Imaging System in the “optimal auto-exposure” mode. The relative quantification of Western Blot bands was performed with the software ImageLab (version 6.1; Bio-Rad Laboratories GmbH) according to a previously published stain-free total-protein normalization method [44]. For each lane, the pixel density of the respective specific protein band was normalized to the total protein of the corresponding lane. As a reference (normalization factor 1), the arithmetic mean value of the total lane volume of the GK sample (n = 3 biological replicates) was used. The arithmetic mean and standard deviation (SD) of each sample were calculated according to the methods in Section 2.11.

### 2.6. Indirect Immunofluorescence (IIF)

To assess the intracellular distribution of proteins, cells were analyzed via IIF according to the previously published internal laboratory standard [38]. In brief, glass microscopy slides (New Erie Scientific LLC, Portsmouth, NH, USA) were sterilized twice in EtOH for 15 min at RT, subsequently washed in deionized water (Merck Millipore, Darmstadt, Germany) and air-dried. Cells were cultured as described in Section 2.1, trypsinized and seeded on the glass slides (final density: 11,500 cells/cm^2^ for FIB, FIB EtOH Ko and FIB EtOH cells; 7700 cells/cm^2^ for all other cell lines). Cells were grown on glass slides for six days, and medium was exchanged after three days (if indicated, in the presence of the EMT cocktail). After six days, cells were washed twice with PBS and fixed with ice-cold 4% [*w*/*v*] paraformaldehyde (Carl Roth GmbH + Co. KG, Karlsruhe, Germany). Until further use, cells were stored in the paraformaldehyde solution at 4 °C.

Before staining, cells were washed in PBS twice. Afterwards, the samples were incubated in a blocking solution containing 5% [*w*/*v*] BSA and 0.1% [*v*/*v*] Triton^®^ X-100 (Sigma-Aldrich) in PBS for 30 min at RT in a shaking incubator. Then, cells were incubated with the respective primary antibody (see Supplementary Table S.M3) overnight at 4 °C. After three washing steps with PBS, samples were subjected to the corresponding secondary antibody (see Supplementary Table S.M3) and Phalloidin-iFluor 594 Reagent (Abcam, Cambridge, UK) or Phalloidin-iFluor 488 Reagent (Abcam) for 1 h at RT in the dark. After three more washing steps with PBS, samples were incubated with DAPI (Invitrogen, Waltham, MA, USA) for 15 min at RT in the dark. Finally, cells were washed with PBS twice and once with deionized water and embedded in Fluoromount-G™ (SouthernBiotech, Birmingham, AL, USA). Detection of fluorescence intensity was performed with the BZ-9000 microscope (Keyence, Neu-Isenburg, Germany). Exposure times were the same in all samples for each protein of interest.

### 2.7. Detection of Reactive Oxygen Species (ROS)

Dichlorodihydrofluorescein-diacetate/2′,7′-Dichlorodihydrofluorescein-diacetate (DCFDA/H2DCFDA)-Cellular ROS Assay Kit (Abcam) was used to detect cellular ROS production. Cells were grown to sub-confluence for six days (if indicated, in the presence of the EMT cocktail). Fluorescence assay-compatible 96-well plates (Invitrogen) were sterilized twice with EtOH (2× 15 min at RT), washed in PBS and incubated overnight at 4 °C with a 10 µg/mL Fibronectin solution in PBS (Sigma-Aldrich) to enable cell adhesion [45]. A total of 25,000 cells in 100 µL of the respective medium (if indicated, in the presence of the EMT cocktail) were seeded in each well. Cells were allowed to adhere for 24 h. The subsequent procedure was performed strictly according to the manufacturer’s protocol [46]. Positive controls with tert-Butyl hydroperoxide (TBHP) were incubated for 4 h. Fluorescence intensity was detected in n = 3 biological replicates in the Infinite^®^ M200 microplate reader. Statistical analysis was performed as described in Section 2.11.

### 2.8. Detection of Anaerobic Cellular Metabolism (L-Lactate Assay)

An L-Lactate Assay Kit (Merck Millipore, Darmstadt, Germany) was used according to the manufacturer’s protocol [47]. A total of 25,000 cells per cell line were seeded into each well of a 96-well plate (if indicated, in the presence of the EMT cocktail). The medium was exchanged after 72 h and 120 h. At 24 h after the last medium exchange (corresponding to a total culture time of 144 h), the reaction was started. Since DMEM and KGM-2 medium both contain phenol red, the reaction conditions were adjusted according to the manufacturer’s recommendations. As it was previously unknown if gingival keratinocytes secrete a relevant amount of lactate dehydrogenase (LDH) into the cell culture supernatant, control reactions without the exogenous addition of LDH were performed to adjust the results for this potential source of L-lactate turnover. The initial absorbance AI565 was measured at a wavelength of 565 nm with the Infinite^®^ M200 microplate reader. After incubation for 20 min at RT, the final absorbance AF565 was measured at the same wavelength. The reactions were performed in n = 3 biological replicates with and without the addition of exogenous LDH. ΔA565,E is the difference between the initial and final absorbance in samples without exogenous LDH, whereas ΔA565,M is the same difference in samples with exogenous LDH. The difference in absorbance, corrected for L-lactate turnover by endogenously secreted LDH, is therefore ΔA565,K = ΔA565,M − ΔA565,E. Absolute L-lactate values were determined using a linear regression model of a calibration curve with known L-lactate concentrations. Statistical analyses were conducted as described in Section 2.11.

### 2.9. Analysis of Cell Proliferation with the iCELLigence™ System

The analysis of cell proliferation was performed with the iCELLigence™ system (OMNI Life Sciences GmbH & Co. KG, Bremen, Germany) using the corresponding E-Plate L8 (OMNI Life Sciences GmbH & Co. KG) [48]. Cells were cultured as described in Section 2.1. Blank measurements were performed with 100 µL of the respective medium. A total of 20,000 cells of each cell line were resuspended in 500 µL of the corresponding medium (if indicated, in the presence of the EMT cocktail) and seeded on the E-Plate L8. After adhesion for 30 min at 37 °C and 5% CO_2_, E-Plate L8 was transferred to the real-time analyzer in the cell culture incubator (37 °C, 5% CO_2_). The cell index (CI) was registered every minute for the first 24 h, and every 15 min for the next 96 h. Complete medium exchange was achieved after 72 h. n = 4 biological replicates were analyzed for each experimental condition. Growth charts were modeled with a non-linear regression based on exponential growth with an upper bound/exponential plateau and an ordinate section point of 0 (GraphPad Prism, version 10.4.2, Graphpad Software, Inc., La Jolla, CA, USA). The maximum value *Y_m_* (corresponding to the upper bound) and the exponential coefficient *k* were analyzed statistically as described in Section 2.11.

### 2.10. Detection of Cellular Migration

An Ibidi^®^ Culture-Insert 2 Well 24 (Ibidi GmbH, Gräfelfing, Germany) was used to monitor cell migration. If the EMT cocktail was used, cells were cultured for six days in the presence of the EMT-inducing cocktail before trypsinization. 70 µL of a 3 × 10^5^ cell/mL solution in the respective medium (if indicated, in the presence of the EMT cocktail) was pipetted into the wells of the culture inserts. After adhesion for 24 h at 37 °C and 5% CO_2_ and reaching confluence, culture inserts were removed, cell layers were washed with phosphate-buffered saline (PBS; Life Technologies GmbH) and covered with culture medium (if indicated, in the presence of the EMT cocktail). The moment of culture insert removal was defined as t_0_ = 0 h, and cell layers had a distance of 500 µm at this moment. After t_1_ = 1 h, t_2_ = 2 h, t_3_ = 3 h, t_4_ = 4 h, t_5_ = 5 h, t_6_ = 6 h, t_7_ = 8 h, t_8_ = 10 h, t_9_ = 12 h, t_10_ = 16 h, t_11_ = 20 h and t_12_ =24 h, cells were assessed via light microscopy at 10-fold magnification (Leica DMIL, Leica AG, Wetzlar, Germany) and images were acquired using a Canon EOS 77D camera (Canon Inc., Tokyo, Japan). Images were uploaded to the visualization platform Roboflow (Roboflow, Des Moines, IA, USA) and segmented with the integrated SAM2-algorithm (Meta, Menlo Park, CA, USA). Segmentation was controlled manually for every image. The images were subsequently analyzed in ImageJ (version 1.54p, NIH, Bethesda, MD, USA) by converting them into an 8-bit binary (black and white) format. The pixels of the cell-free area were counted using the integrated “area measurement” tool. Data from n = 3 biological replicates were analyzed as described in Section 2.11.

### 2.11. Statistical Analysis

For all experiments, data were analyzed and visualized using GraphPad Prism (version 10.4.2, Graphpad Software Inc., La Jolla, CA, USA).

First, it was analyzed whether there is a statistically significant difference in the respective values between the cell lines GK, GK EtOH Ko, GK EtOH, EPI, EPI EtOH Ko, EPI EtOH, FIB, FIB EtOH Ko and FIB EtOH. To this end, one-way analysis of variance (ANOVA) was applied to compare mean values, assuming normal distribution of measurements [49]. If *p*-values < 0.05 were detected, the Tukey–Kramer test (Tukey Honest Significant Difference post hoc Test) was applied for pairwise comparison of the respective cell lines. The latter test corrects for multiple testing [50]. Test results were evaluated using commonly available q-value charts (α = 0.05) [51].

For experiments making use of the EMT cocktail, it was analyzed whether there exists a statistically significant difference between treated (“+EMT”) and untreated (“−EMT”) conditions, to directly assess the effect of the intervention. Pairwise comparison was performed using an unpaired, two-tailed *t*-test (assuming normal distribution with homogenous variances between groups). Results were considered statistically significant if *p* < 0.05.

## 3. Results

### 3.1. EtOH and EMT Cocktail Treatment-Induced Distinct Morphological Changes in Gingival Keratinocytes

The three cell lines GK, EPI and FIB served as the basis for subsequent experiments. Each cell line was continuously exposed to 5‰ [*v*/*v*] EtOH for two months in a newly developed, closed culturing system (see Section 2.2; new cell lines are recognizable by the additional ending “EtOH”). To control for possible effects of the closed atmosphere, additional lines were cultured under identical conditions without EtOH (designated “EtOH Ko”). After treatment, nine different stable cell lines were used for further analyses: GK, GK EtOH Ko, GK EtOH, EPI, EPI EtOH Ko, EPI EtOH, FIB, FIB EtOH Ko and FIB EtOH (see Supplementary Table S.M1).

**Figure 1:**
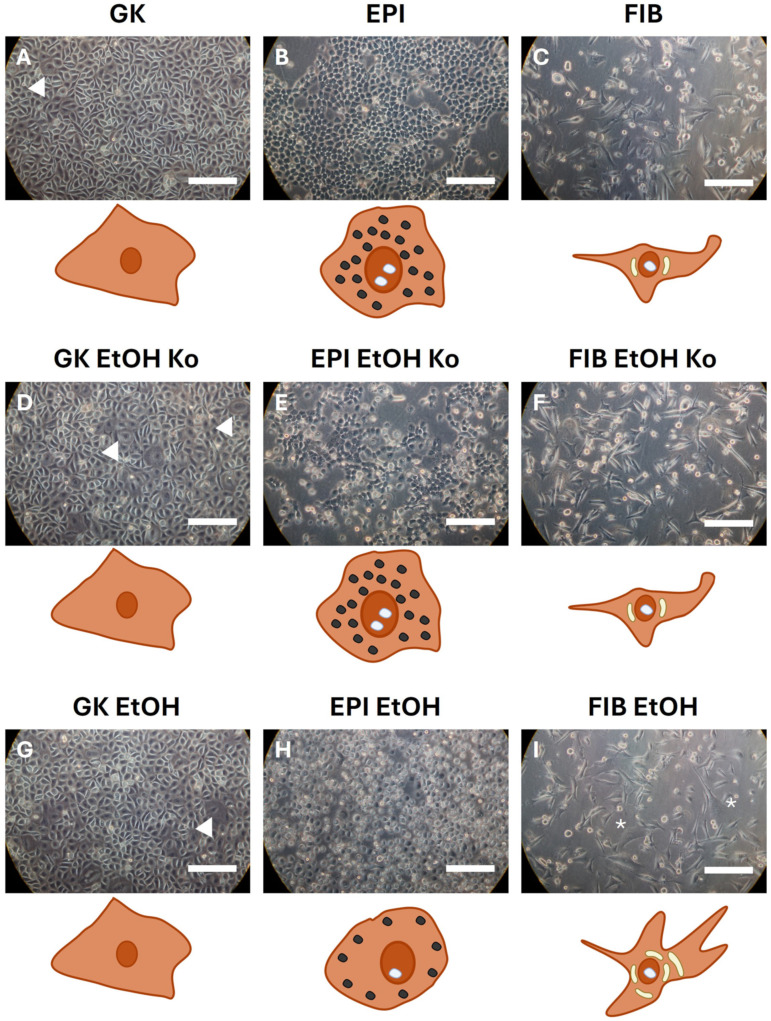
EtOH-induced morphological changes in gingival keratinocytes as detected by phase-contrast light microscopy. Representative light micrographs (upper panels) and schematic cell illustrations (lower panels) are depicted. (**A**–**C**): Parental cell lines (GK, EPI, FIB). (**D**–**F**): Closed-culture controls (GK EtOH Ko, EPI EtOH Ko, FIB EtOH Ko). (**G**–**I**): EtOH-treated cell lines (GK EtOH, EPI EtOH, FIB EtOH). Light brown = cytoplasm; dark brown = nucleus; white spots within nucleus = nucleoli; black circles = light-absorbing granules; yellow = perinuclear vacuoles; white arrowheads = giant cells; white asterisks = enlarged cell bodies. Scale bars = 200 µm. Created in Biorender. Steinberg, T. (2025) https://BioRender.com/pnqrfh0.

Figure 1 shows representative light micrographs (20-fold magnification) at (sub-)confluence and schematic sketches of the corresponding cell morphologies. GK (Figure 1A), GK EtOH Ko (Figure 1D) and GK EtOH (Figure 1G) cells showed a similar, clubbed, polygonal morphology with translucent cell borders. Occasionally, multinucleated giant cells (white arrowheads) could be detected [52].

EPI formed densely packed epithelial layers with clearly visible nucleoli and a high nuclear–cytoplasmic ratio. The cytoplasm contained numerous light-absorbing granules (Figure 1B; [31]). EPI EtOH Ko cells appeared comparable but showed reduced cell density (Figure 1E). EPI EtOH cells lost the cobblestone-like pattern and displayed round to oval morphologies with blurred cell borders and decreased adherence to the culture surface (own observations; Figure 1H).

FIB cells presented fibroblast-like and spindle-shaped cell bodies with dendritic processes, perinuclear vacuoles and prominent nucleoli (Figure 1C). FIB EtOH Ko cells were morphologically similar to FIB cells (Figure 1F). FIB EtOH cells exhibited enlarged cell bodies and had an increased number of perinuclear vacuoles (white asterisks in Figure 1I).

Upon exposure to the EMT-inducing cocktail (“+EMT”; see Section 2.3), GK (Supplementary Figure S1A,B), GK EtOH Ko (Supplementary Figure S1G,H) and GK EtOH (Supplementary Figure S1M,N) cells showed no apparent morphological changes. In contrast, EPI (Supplementary Figure S1C,D) and EPI EtOH Ko (Supplementary Figure S1I,J) cells displayed a partial detachment of single, roundish cells with granular cytoplasm and an overall reduced cell density. EPI EtOH cells (Supplementary Figure S1O,P) appeared unchanged in response to “+EMT” treatment. FIB cells exhibited elongated cell bodies and a grouped growth pattern (Supplementary Figure S1E,F), whereas FIB EtOH Ko (Supplementary Figure S1K,L) and FIB EtOH (Supplementary Figure S1Q,R) cells grew in a storiform pattern in the presence of the EMT-inducing medium.

In summary, culturing in closed flasks alone did not alter cell morphology. However, both EtOH and “+EMT” treatment induced distinct cellular and organellar changes. Storiform growth patterns were exclusively observed after “+EMT” treatment, with additive effects in FIB EtOH Ko and FIB EtOH cells. Overall, the EPI and FIB lineages were more susceptible to both EtOH and “+EMT” treatment when compared to GK cells.

A schematic summary of morphological features is provided in Supplementary Figure S2.

### 3.2. EtOH Treatment Modulated Adhesion- and EMT-Associated Gene Expression in GK, EPI and FIB Cells

**Figure 2:**
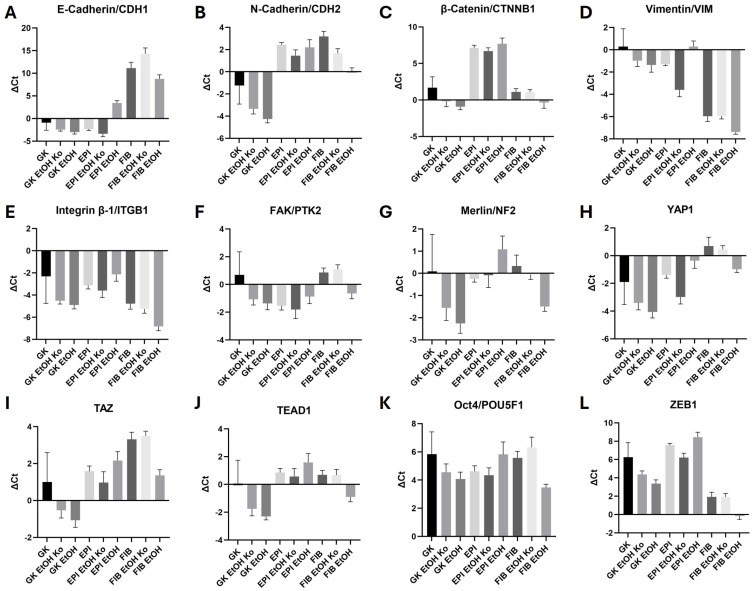
EtOH-dependent transcriptional modulation of AJ, FA and Hippo signaling components and EMT-associated genes in GK, EPI and FIB cells and their derivatives. qPCR analysis of twelve target genes in parental, closed-culture (EtOH Ko) and EtOH-treated (EtOH) cell lines. The bars represent mean ΔCt values (normalized to reference genes) with the corresponding SDs of n = 3 biological replicates (high ΔCt values correspond to a low gene expression and vice versa). For detailed statistical evaluation, see Supplementary Tables S1–S12. (**A**): *CDH1*; (**B**): *CDH2*; (**C**): *CTNNB1*; (**D**): *VIM*; (**E**): *ITGB1*, (**F**): *PTK2*; (**G**): *NF2*; (**H**): *YAP1*; (**I**): *TAZ*; (**J**): *TEAD1*; (**K**): *POU5F1* and (**L**): *ZEB1*. The diagrams were created with GraphPad Prism.

To examine whether chronic EtOH treatment affects the transcriptional regulation of adhesion- and EMT-related signaling, qPCR analyses were performed for twelve target genes representing AJs, FAs and the Hippo pathway. Mean ΔCt ± SD values are depicted in Figure 2 (note that higher ΔCt values correspond to lower relative gene expression). Statistical data are summarized in Supplementary Tables S1–S12.

*AJ Markers*

As expected for epithelial cells, GK, GK EtOH Ko and GK EtOH cells displayed a high *CDH1* expression (encoding epithelial E-Cadherin) without significant differences. EPI and EPI EtOH Ko cells exhibited comparable amounts of *CDH1* transcripts, whereas EPI EtOH cells showed a statistically significant reduction. *CDH1* expression was scarce in FIB cells and their derivatives, consistent with their fibroblast-like morphology. The differences between GK and EPI cells, as well as their derivatives, were statistically significant (Figure 2A, Supplementary Table S1). GK EtOH Ko and GK EtOH cells exhibited a significantly higher *CDH2* expression (encoding mesenchymal neuronal (N)-Cadherin) when compared to GK cells. Conversely, EPI cells and their derivatives, as well as FIB and FIB EtOH Ko cells, had a significantly lower *CDH2* expression. EtOH treatment led to a moderate but significant rise in *CDH2* transcripts in FIB EtOH relative to FIB cells (Figure 2B, Supplementary Table S2). ΔCt values of *CTNNB1* (encoding β-Catenin) were similar in GK and GK EtOH Ko cells, as well as in FIB cells and their derivatives. EtOH treatment induced a significant increase in *CTNNB1* in GK EtOH cells. In contrast, EPI cells and their derivatives had statistically significantly lower levels of *CTNNB1*-RNA than GK cells (Figure 2C, Supplementary Table S3).

*Vimentin and FA components*

Expression of the mesenchymal intermediate filament *Vimentin* was low in GK, GK EtOH Ko, GK EtOH, EPI and EPI EtOH cells. EPI EtOH Ko cells showed moderately higher *VIM* levels (*p* < 0.05), while FIB cells and their derivatives exhibited the highest overall expression. EtOH treatment caused a further, non-significant *VIM* increase in FIB EtOH cells (Figure 2D, Supplementary Table S4). *ITGB1* transcripts (encoding Integrin β-1) were abundant across all lines with no statistically significant differences (Figure 2E, Supplementary Table S5). The analysis of *PTK2* expression (encoding the focal adhesion kinase FAK) showed variable expression with homogeneous ΔCt values in GK, GK EtOH Ko, EPI EtOH, FIB, FIB EtOH Ko and FIB EtOH cells and a higher number of transcripts in GK EtOH, EPI and EPI EtOH Ko cells (Figure 2F, Supplementary Table S6).

*Hippo pathway and EMT transcription factors (TFs)*

The expression profiles of *neurofibromatosis type 2* (*NF2*; encoding the protein Merlin), *Yes-associated protein 1 (YAP1)*, *transcriptional co-activator with PDZ-binding motif (TAZ)* and *TEA domain family members 1 (TEAD1)* showed similar patterns. Expression was highest in GK EtOH cells, whereas FIB and FIB EtOH Ko cells showed lower *YAP1* and *TAZ* levels compared with GK cells (Figure 2G–J, Supplementary Tables S7–S10). *POU5F1* (encoding Oct4) was altered only in FIB cells upon EtOH treatment (Figure 2K, Supplementary Table S11). *Homologue zinc finger E-box-binding homeobox 1 (ZEB1)* expression increased significantly in GK EtOH and FIB EtOH cells, indicating partial transcriptional activation of EMT-related genes in these cell lines (Figure 2L, Supplementary Table S12). 

A recapitulatory view of these findings is given in Supplementary Figure S3.

### 3.3. The EMT-Inducing Cocktail Triggered Vimentin Expression and Modulated AJ Components

**Figure 3:**
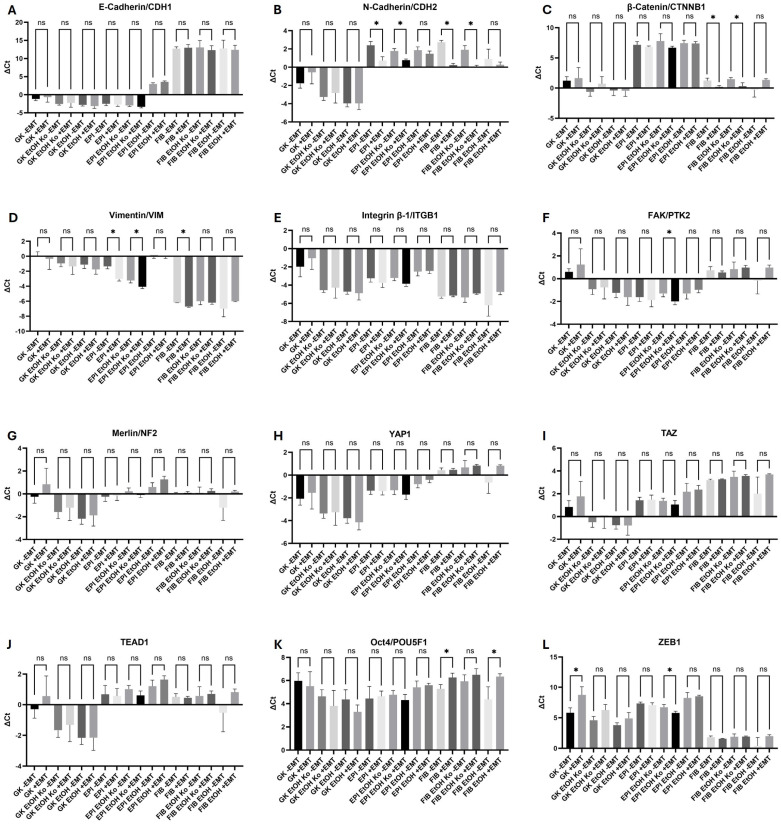
EMT cocktail-induced transcriptional modulation of AJ, FA and Hippo signaling components and EMT-associated genes in GK, EPI and FIB cells and their derivatives. qPCR analysis of twelve target genes in parental, closed-culture (EtOH Ko) and EtOH-treated (EtOH) cell lines under untreated (“−EMT”) and EMT cocktail-treated (“+EMT”) conditions. The bars represent mean ΔCt values (normalized to reference genes) with the corresponding SDs of n = 3 biological replicates (high ΔCt values correspond to a low gene expression and vice versa). Statistical significance was determined by pairwise comparison (“−EMT” vs. “+EMT”). For detailed statistical evaluation, see Supplementary Table S13. (**A**): *CDH1*; (**B**): *CDH2*; (**C**): *CTNNB1*; (**D**): *VIM*; (**E**): *ITGB1*, (**F**): *PTK2*; (**G**): *NF2*; (**H**): *YAP1*; (**I**): *TAZ*; (**J**): *TEAD1*; (**K**): *POU5F1* and (**L**): *ZEB1*. ns = not significant; * = *p* < 0.05 (statistically significant). The diagrams were created with GraphPad Prism.

To assess the transcriptional effects of biochemical EMT induction, all cell lines were treated with the EMT-inducing cocktail as described in Section 2.3. Gene expression was quantified by qPCR and analyzed in pairwise comparisons between untreated (“−EMT”) and treated (“+EMT”) conditions (Section 2.11). Mean ΔCt ± SD values are depicted in Figure 3 (note that higher ΔCt values correspond to lower relative gene expression). Corresponding *p*-values are summarized in Supplementary Table S13.

*AJ and FA markers:*

*CDH1* transcripts were not significantly altered by “+EMT” treatment across all cell lines (Figure 3A). In contrast, *CDH2* expression increased significantly in EPI and FIB cells following exposure to the EMT cocktail (Figure 3B). *CTNNB1* levels rose significantly in FIB cells (Figure 3C), whereas *ITGB1* (Figure 3E) and *PTK2* (Figure 3F) transcripts displayed variable, non-systematic regulation.

*Vimentin and Hippo pathway components:*

The expression of *VIM* was upregulated in most cell lines under “+EMT” conditions, confirming transcriptional responsiveness to biochemical EMT induction. An exception was FIB EtOH cells, where *VIM* decreased slightly relative to untreated controls (Figure 3D). The gene expression of *NF2*, *YAP1*, *TAZ* and *TEAD1* was synchronously modulated in all cell lines. “+EMT” treatment led to a decrease in gene transcripts in GK, EPI EtOH (exception *TAZ*) and FIB EtOH cells (Figure 3G–J).

*EMT TFs*

Contrary to the other findings, the EMT-associated marker genes POU5F1 and ZEB1 were mainly influenced by the EMT cocktail in GK cells and their derivatives (Figure 3K,L).

Overall, VIM and AJ-associated genes were most responsive to EtOH and “+EMT” treatment. EPI and FIB cells, as well as their derivatives, displayed stronger transcriptional responses than GK cells upon “+EMT” treatment, indicating higher EMT susceptibility and thus a more advanced state of transformation.

### 3.4. EtOH Treatment Altered the Abundance of Mechanobiologically Relevant Marker Proteins in GK, EPI and FIB Cells

**Figure 4:**
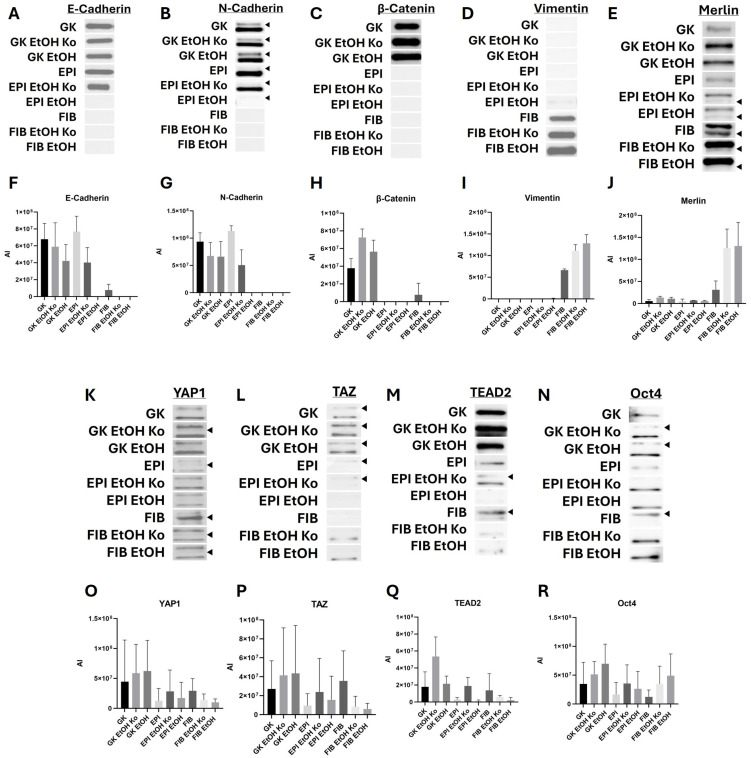
Western Blot analysis of mechanobiological and EMT-associated marker proteins in GK, EPI and FIB cells and their derivatives. Representative immunoblots (upper panels) and quantitative densitometry (lower panels) are depicted for selected proteins. The bars show mean AIs (absolute intensities) with the corresponding SDs of n = 3 biological replicates. Black arrowheads indicate additional specific protein bands. Statistical details can be found in Supplementary Tables S14–S22. (**A**,**F**): E-Cadherin. (**B**,**G**): N-Cadherin. (**C**,**H**): β-Catenin. (**D**,**I**): Vimentin. (**E**,**J**): Merlin. (**K**,**O**): YAP1. (**L**,**P**): TAZ. (**M**,**Q**): TEAD2. (**N**,**R**): Oct4. The diagrams were created with GraphPad Prism.

Following the transcriptional analyses, the levels of selected marker proteins were examined by SDS-PAGE, Western Blot, immunodetection and quantitative densitometry. Figure 4 depicts representative immunoblots and quantification data. Statistical analyses are listed in Supplementary Tables S14–S22.

*AJ markers*

Consistent with the qPCR findings, GK cells and their derivatives, which retained an epithelial morphology, showed strong E-Cadherin signals without significant differences. EPI and EPI EtOH Ko cells also displayed prominent E-Cadherin bands, whereas the signal was markedly reduced in EPI EtOH cells. In FIB cells and their derivatives, the E-Cadherin signal was weak or undetectable (Figure 4A,F, Supplementary Table S14). N-Cadherin abundance followed a similar pattern in all cell lines. An additional, distinct N-Cadherin protein band could be detected, whose intensity was differentially modulated in the different cell lines (Figure 4B,G, Supplementary Table S15; [53,54]). β-Catenin was most abundant in GK EtOH Ko cells, whereas EPI and FIB cells and their derivatives exhibited faint or absent signals (Figure 4C,H, Supplementary Table S16).

*Vimentin*

Vimentin was barely detectable in GK and EPI cells, as well as their derivatives, but present at high levels in FIB, FIB EtOH Ko and FIB EtOH cells (increasing in this order). The differences between FIB and FIB EtOH Ko cells and between FIB and FIB EtOH cells were statistically significant (Figure 4D,I, Supplementary Table S17).

*Hippo pathway and EMT TFs*

Merlin showed intense protein bands in FIB EtOH Ko and FIB EtOH cells (*p* < 0.05 vs. all other cell lines). Additional specific protein bands could be detected in EPI EtOH Ko, EPI EtOH, FIB, FIB EtOH Ko and FIB EtOH cells, whose relative intensity differed considerably between cell lines (Figure 4E,J, Supplementary Table S18; [55]). For YAP1, TAZ, TEAD2 and Oct4, total protein levels did not differ significantly between groups (Figure 4K–R; Supplementary Tables S19–S22). Multiple bands of varying molecular weight were observed for all four proteins, indicating the presence of post-translationally modified isoforms [56,57].

A graphical summary of the findings concerning protein levels is provided in Supplementary Figure S4.

### 3.5. Biochemical EMT Induction Predominantly Modulated Proteins of the Cadherin–Catenin System

**Figure 5:**
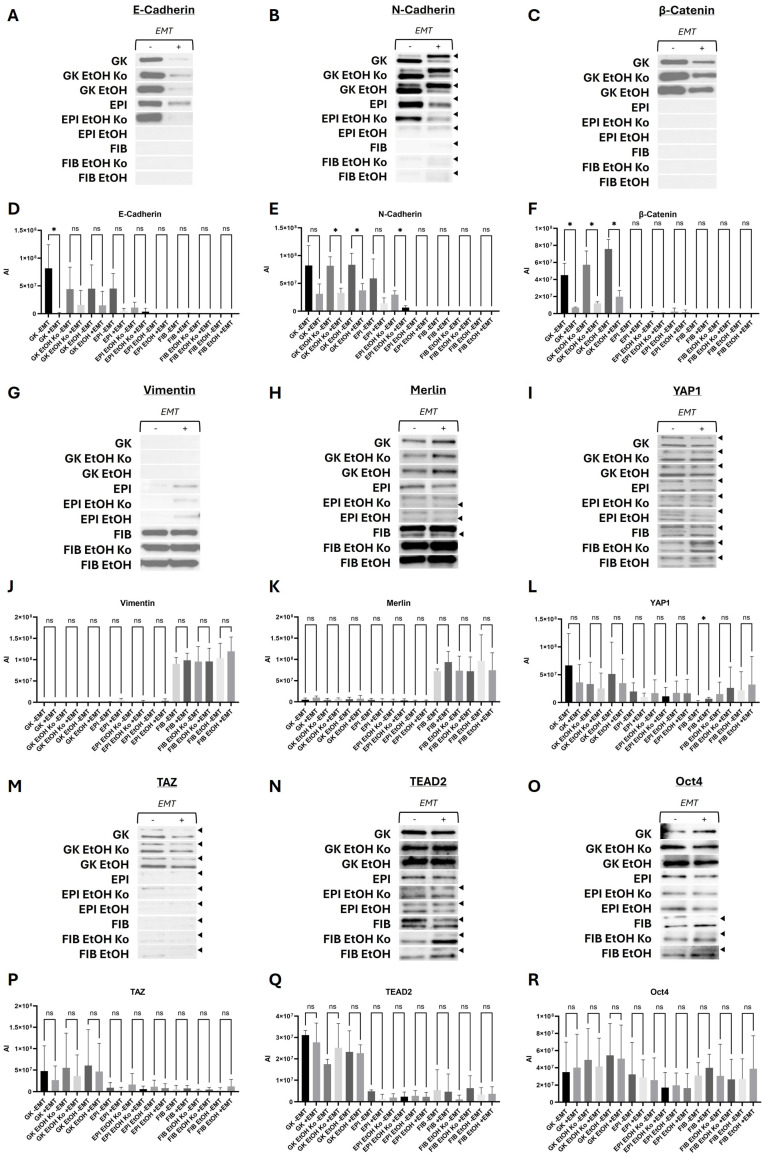
Western Blot analysis of mechanobiological and EMT-associated marker proteins in GK, EPI and FIB cells and their derivatives. Representative immunoblots (upper panels) and quantitative densitometry (lower panels) are depicted for selected proteins in untreated (“−EMT”) and treated (“+EMT”) cell lines. The bars show mean AIs (absolute intensities) with the corresponding SDs of n = 3 biological replicates. Black arrowheads indicate additional specific protein bands. Statistical details can be found in Supplementary Table S23. (**A**,**D**): E-Cadherin. (**B**,**E**): N-Cadherin. (**C**,**F**): β-Catenin. (**G**,**J**): Vimentin. (**H**,**K**): Merlin. (**I**,**L**): YAP1. (**M**,**P**): TAZ. (**N**,**Q**): TEAD2. (**O**,**R**): Oct4. ns = not significant; * = *p* < 0.05 (statistically significant). The diagrams were created with GraphPad Prism.

To determine the effects of biochemical EMT induction on protein abundance, the established cell lines were treated with the EMT-inducing cocktail as described in Section 2.3. Western Blot analyses and quantitative densitometry were performed for the same marker proteins as in Section 3.4. Representative immunoblots and quantification data are shown in Figure 5, and corresponding statistical results are summarized in Supplementary Table S23.

*AJ markers*

E-Cadherin protein levels were markedly reduced after “+EMT” treatment in GK cells and their derivatives, as well as in EPI and EPI EtOH Ko cells. This was consistent with the presence of E-Cadherin-blocking antibodies in the EMT cocktail (Figure 5A,D, Supplementary Table S23). N-Cadherin exhibited a similar response pattern to biochemical EMT induction. Protein levels decreased significantly in GK EtOH Ko, GK EtOH and EPI EtOH Ko cells. Moreover, the relative intensity of the additional specific protein bands, as indicated by the black arrowheads in Figure 5, changed upon “+EMT” treatment (Figure 5B,E, Supplementary Table S23). β-Catenin amounts were significantly reduced in GK cells and their derivatives following EMT induction, reflecting the destabilization of AJs and loss of junctional β-Catenin anchorage (Figure 5C,F, Supplementary Table S23).

*Vimentin*, *Hippo pathway and EMT TFs*

Vimentin (Figure 5G,J), Merlin (Figure 5H,K), TAZ (Figure 5M,P), TEAD2 (Figure 5N,Q) and Oct 4 (Figure 5O,R) did not show statistically significant changes upon EMT treatment (Supplementary Table S23). However, several regulatory trends were evident, which are schematically summarized in Supplementary Figure S4. Vimentin amounts tended to increase in EPI and FIB cells, as well as in their derivatives, indicating partial cytoskeletal reorganization. YAP1 and TAZ protein amounts decreased moderately in GK cells and their derivatives, while they increased in FIB cells and their derivatives upon “+EMT” treatment. Treatment with the EMT cocktail again influenced the relative abundance of additional specific protein bands of several proteins, including YAP1 (Figure 5I,L), TAZ (Figure 5M,P), TEAD2 (Figure 5N,Q) and Oct 4 (Figure 5O,R; see also Section 3.4).

In summary, neither EtOH treatment nor closed-culture conditions alone substantially altered the protein abundance of Hippo pathway components. In contrast, biochemical EMT induction notably affected proteins of the Cadherin–Catenin system, particularly E-Cadherin, N-Cadherin and β-Catenin. The concurrent decrease in E-Cadherin and upregulation of Vimentin in FIB cells and their derivatives is consistent with EMT-associated cytoskeletal remodeling. The highest responsiveness towards EtOH and “+EMT” treatment was detected in FIB and FIB EtOH cells, where the effects of both treatments even appeared additive.

### 3.6. EtOH and “+EMT” Treatment Primarily Influenced Subcellular Localization of Proteins from the Cadherin–Catenin System and Vimentin

Since simple Western Blot analysis does not resolve subcellular protein localization, all cell lines were examined by indirect immunofluorescence (IIF). Due to considerable autofluorescence in all cell cultures, IIF intensities were evaluated qualitatively. Representative IIF micrographs are depicted in Figure 6 and Figure 7, while extended datasets are provided in Supplementary Figures S5–S24. A schematic summary of the major findings is presented in Supplementary Figure S25.

*AJ markers*

**Figure 6:**
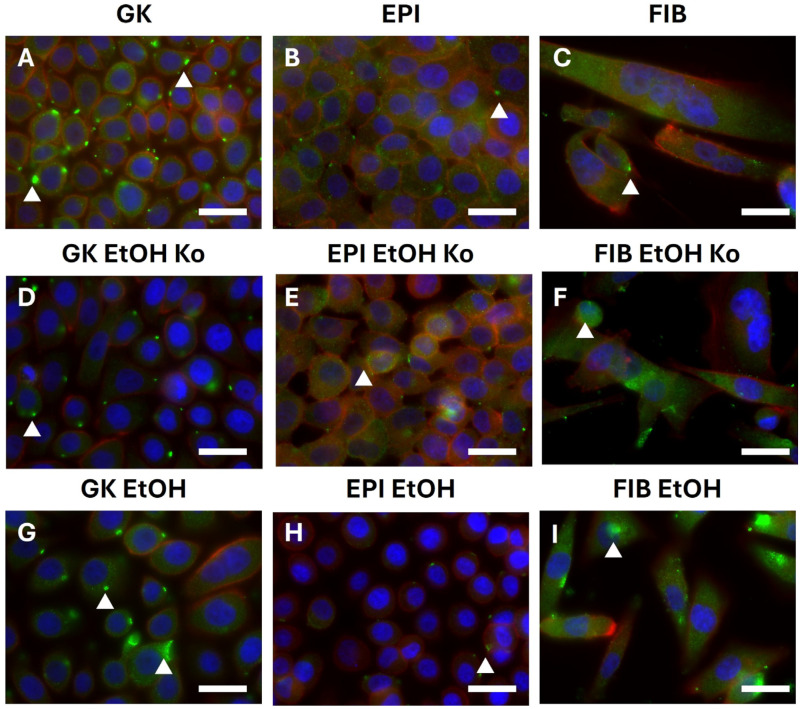
Indirect immunofluorescence (IIF) micrographs with specific detection of N-Cadherin in GK, EPI and FIB cells as well as their derivatives. The specific protein signal is depicted in green (see white arrowheads), the cellular actin cytoskeleton in red and cell nuclei in blue. (**A**): GK cells; (**B**): EPI cells; (**C**): FIB cells; (**D**): GK EtOH Ko cells; (**E**): EPI EtOH Ko cells; (**F**): FIB EtOH Ko cells; (**G**): GK EtOH cells; (**H**): EPI EtOH cells; (**I**): FIB EtOH cells. Scale bars = 40 μm.

E-Cadherin displayed a predominantly perimembranous fluorescence pattern and was mainly detected in GK and EPI cells and their derivatives, which is consistent with the qPCR and Western Blot findings (Supplementary Figure S5). After EMT cocktail exposure, perinuclear puncta of increased E-Cadherin signal appeared in EPI and FIB cells and their derivatives, indicating altered intracellular distribution (white arrowheads in Supplementary Figure S6). It might be speculated that the E-Cadherin-blocking antibodies in the EMT cocktail inhibited E-Cadherin shuttling to the plasma membrane or induced E-Cadherin endocytosis from the plasma membrane. In agreement with published observations, N-Cadherin localized to inclusion body-like cytoplasmic structures of high fluorescence intensity (white arrowheads in Figure 6; [58,59]). These inclusions were most pronounced in GK cells and their derivatives. In EPI cells and their derivatives, the detection of these inclusions was hampered by strong autofluorescence; however, signal intensity was clearly reduced in EPI EtOH cells (Figure 6H). “+EMT” treatment induced a subtle increase in N-Cadherin staining in GK and FIB cells as well as their derivatives (Supplementary Figure S7). β-Catenin was mainly localized at the plasma membrane in GK cells and their derivatives, while it was detected in the nucleus of EPI and FIB cells and their derivatives (white arrowheads and white asterisks in Supplementary Figure S8). “+EMT” treatment markedly reduced β-Catenin staining in GK and GK EtOH cells, mirroring the Western Blot data (Supplementary Figure S9).

*Vimentin and FA components*

**Figure 7:**
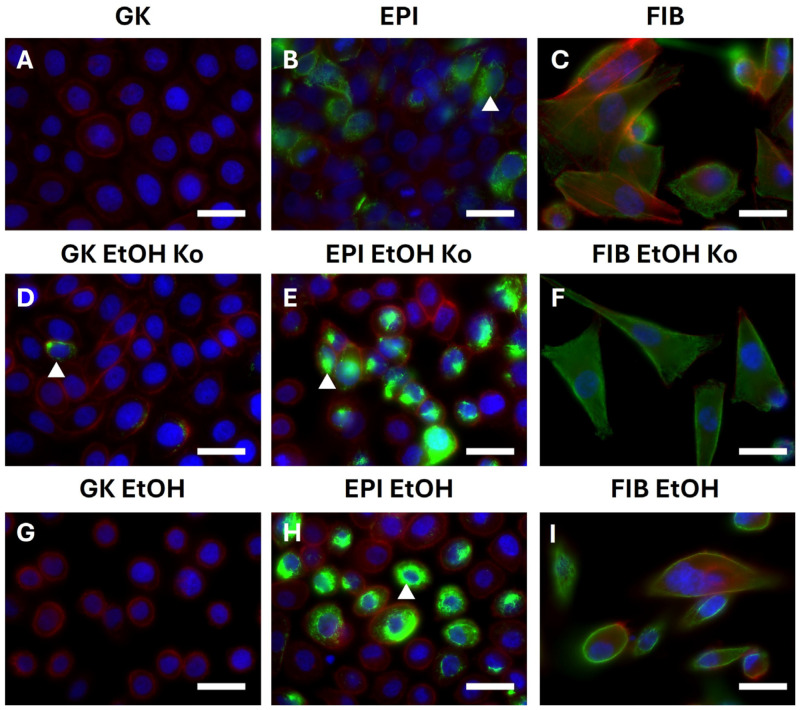
Indirect immunofluorescence (IIF) micrographs with specific detection of Vimentin in GK, EPI and FIB cells as well as their derivatives. The specific protein signal is depicted in green (see white arrowheads), the cellular actin cytoskeleton in red and cell nuclei in blue. (**A**): GK cells; (**B**): EPI cells; (**C**): FIB cells; (**D**): GK EtOH Ko cells; (**E**): EPI EtOH Ko cells; (**F**): FIB EtOH Ko cells; (**G**): GK EtOH cells; (**H**): EPI EtOH cells; (**I**): FIB EtOH cells. Scale bars = 40 μm.

Vimentin was largely absent from GK cells and their derivatives, confirming the results of the qPCR and Western Blot analyses. In EPI cells and especially in EPI EtOH Ko and EPI EtOH cells, an increasing proportion of cells exhibited a specific, perinuclearly clustered Vimentin fluorescence signal (white arrowheads in Figure 7). In FIB cells and their derivatives, cells showed uniform Vimentin staining throughout the cytoplasm, which intensified after EMT induction (Supplementary Figure S10). pFAKY397, the autophosphorylated and activated form of FAK, was distributed evenly in the cytoplasm of GK and EPI cells and their derivatives, while it clustered at discrete cytoplasmic foci in FIB cells and their derivatives, which is consistent with active FAs (white arrowheads in Supplementary Figure S11). “+EMT” treatment did not substantially alter this pattern (Supplementary Figure S12).

*Hippo pathway and EMT TFs*

Merlin exhibited a diffuse cytoplasmic localization with increased fluorescence in EPI cells and their derivatives, as well as in FIB and FIB EtOH cells (Supplementary Figure S13). The addition of the EMT cocktail enhanced the signal intensity in EPI cells and their derivatives (Supplementary Figure S14). YAP1 (Supplementary Figure S15) and TAZ (Supplementary Figure S17) displayed similar cytoplasmic and perinuclear distributions in all cell lines. After “+EMT” treatment, the fluorescence intensity modestly increased in FIB cells and their derivatives (Supplementary Figures S16 and S18). TEAD1 staining yielded heterogeneous results (Supplementary Figure S19) and did not change substantially upon “+EMT” treatment (Supplementary Figure S20). Oct4 localized to discrete cytoplasmic inclusion body-like structures, resembling those observed for N-Cadherin (white arrowheads in Supplementary Figure S21). Biochemical EMT induction did not significantly affect this pattern (Supplementary Figure S22). ZEB1 exhibited low overall abundance in GK and EPI cells and their derivatives. Perinuclear staining was, however, increased in GK EtOH and EPI EtOH cells. In FIB cells and their derivatives, total IIF intensity was higher (Supplementary Figure S23). Treatment with the EMT cocktail led to an increase in IIF intensity in FIB EtOH cells and to a nuclear redistribution of ZEB1 in all cell lines (Supplementary Figure S24).

Taken together, in accordance with qPCR and Western Blot data, IIF analysis demonstrated that EtOH and “+EMT” treatment primarily influenced proteins of the Cadherin–Catenin system and the intermediate filament Vimentin. Furthermore, subtle but consistent shifts in subcellular localization were observed across treatments, indicating altered cytoskeletal organization and junctional remodeling.

### 3.7. Cellular ROS Production Increased in Response to EtOH and “+EMT” Treatment

After characterizing morphological, transcriptional and protein-level changes, metabolic alterations in the cell lines were analyzed next. Cellular ROS production was quantified as described in Section 2.7. The statistical findings are summarized in Supplementary Tables S24 and S25.

**Figure 8:**
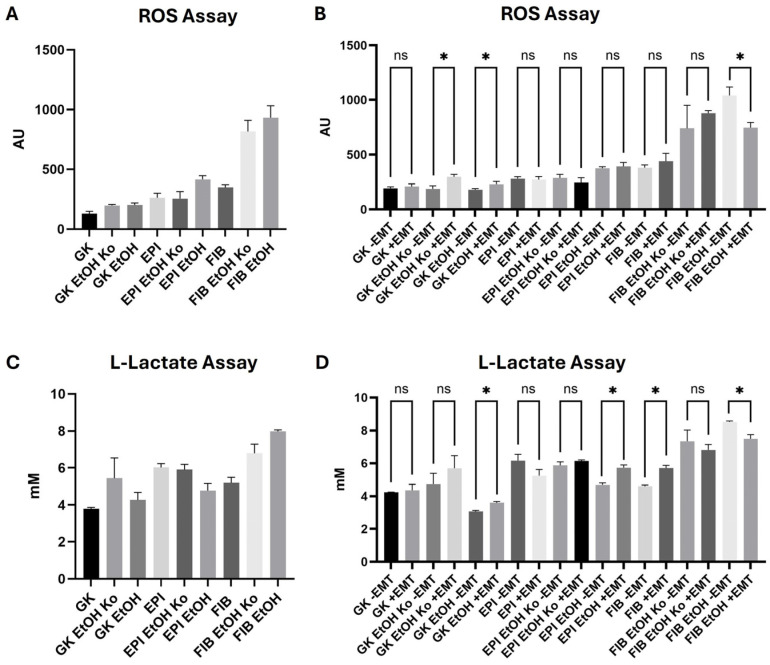
Cellular ROS and L-lactate production in response to EtOH or “+EMT” treatment in GK, EPI and FIB cells and their derivatives. Mean values and the corresponding SDs are shown for n = 3 biological replicates. Statistical data are provided in Supplementary Tables S24–S27. (**A**): Cellular ROS production in the indicated cell lines. (**B**): Cellular ROS production in untreated (“−EMT”) and treated (“+EMT”) cell lines. (**C**): Cellular L-lactate production in the indicated cell lines. (**D**): Cellular L-lactate production in untreated (“−EMT”) and treated (“+EMT”) cell lines. ns = not significant; * = *p* < 0.05 (statistically significant). AU = arbitrary unit; concentrations in mM. The diagrams were created with GraphPad Prism.

As shown in Figure 8A, EtOH treatment significantly elevated ROS levels in EPI and FIB cells. The differences between FIB and GK cells, as well as between FIB EtOH cells and all other cell lines except FIB EtOH Ko, were statistically significant. Upon biochemical EMT induction, ROS levels tended to increase in most cell lines except for EPI EtOH Ko and FIB EtOH. Interestingly, ROS production significantly decreased in FIB EtOH cells when compared to untreated controls (Figure 8B).

In summary, EtOH treatment strongly enhanced cellular ROS production in all cell lines, indicating cellular stress and altered redox homeostasis. “+EMT” treatment produced additive effects in most cases, whereas FIB EtOH cells exhibited a paradoxical reduction, suggesting a partial redox adaptation under chronic EtOH exposure.

### 3.8. Cellular L-Lactate Production Was Differentially Modulated by EtOH and “+EMT” Treatment

According to the Warburg hypothesis, malignant cellular transformation goes along with metabolic reprogramming leading to an increase in anaerobic glycolysis [60]. Thus, to further assess metabolic responses, L-lactate concentrations were determined as indicators of anaerobic glycolytic activity (see Section 2.8). Statistical details are presented in Supplementary Tables S26 and S27.

As shown in Figure 8C, EPI and FIB cells exhibited significantly higher L-lactate levels than GK cells. Notably, EPI cells produced more L-lactate than FIB cells. EtOH treatment led to increased L-lactate release in GK and FIB cells (statistically significant for FIB EtOH vs. FIB cells), whereas EPI EtOH cells showed reduced concentrations relative to EPI cells. In closed-culture controls (“EtOH Ko”), namely GK EtOH Ko and FIB EtOH Ko, cells displayed elevated L-lactate levels, consistent with metabolic adaptation under hypoxic culture conditions.

Treatment with the EMT cocktail led to a statistically significant increase in L-lactate production in GK EtOH, EPI EtOH and FIB cells (Figure 8D). Interestingly, the metabolite levels decreased in EPI, FIB EtOH Ko and FIB EtOH cells (*p* < 0.05 for the latter result). L-lactate production in all “EtOH” cell lines was thus modulated by “+EMT” treatment. The effects of EtOH and “+EMT” treatment were additive in GK EtOH cells but inverse in EPI EtOH and FIB EtOH cells.

A schematic summary of the described metabolic changes is given in Supplementary Figure S26. Collectively, these results indicate that EtOH and biochemical EMT stimuli jointly affect both redox balance and glycolytic activity in a cell-type-dependent manner, with FIB cells and their derivatives showing the strongest metabolic plasticity.

### 3.9. GK, EPI and FIB Cells and Their Derivatives Displayed Distinct Proliferation Kinetics

As a functional indicator of cell transformation, cell proliferation was quantified by recording growth dynamics using the impedance-based iCELLigence™ real-time cell analysis (RTCA) system.

**Figure 9:**
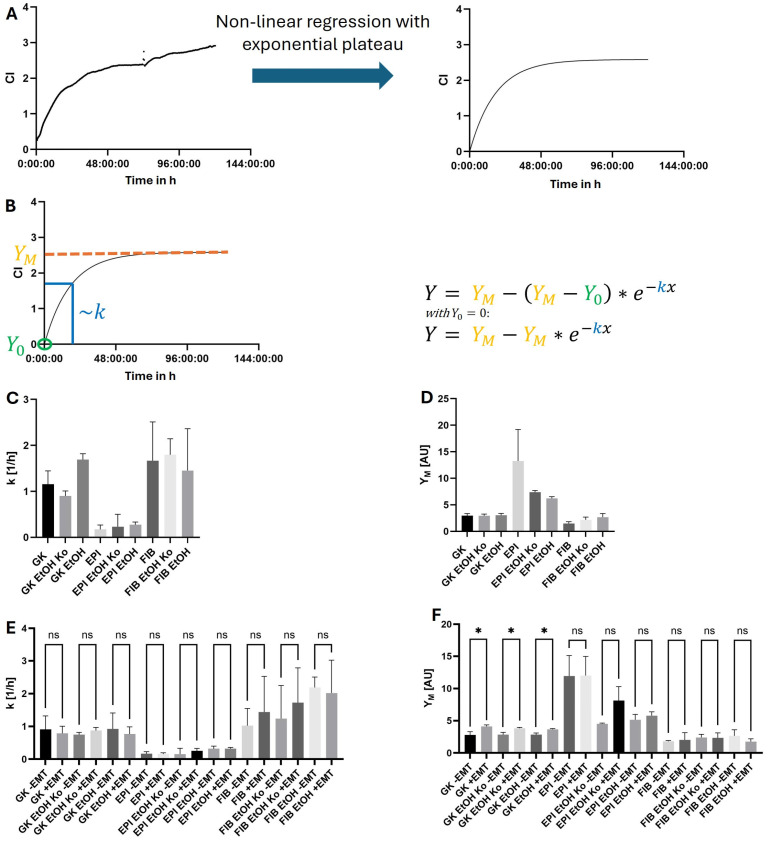
Analysis of cell proliferation with the real-time cell analysis (RTCA) iCELLigence™-system in GK, EPI and FIB cells and their derivatives. (**A**): Left side: exemplary raw data of a 120 h cell proliferation experiment. The break at 72 h resulted from medium exchange. The asymptotic growth curve was modeled by a non-linear regression with an exponential plateau (right side). (**B**): The formula on the right side (upper section) is the mathematical basis for modeling. The ordinate intersection *Y*_0_ was set to 0 for all experiments since the number of initial cells was the same in all cell lines. This simplifies the formula (lower section). The scheme illustrates the meaning of the respective parameters: *Y*_0_ (green) is the ordinate intersection at t = 0 h; *Y_M_* (yellow) is the upper bound/maximum value; the coefficient *k* (blue) defines how fast the function reaches 1 − $\frac{1}{e}$ of *Y_M_*, corresponding to 63.2% of *Y_M_*. (**C**,**D**): Statistical analysis of *k* (**C**) and *Y_M_* (**D**) in the indicated cell lines. The mean values and corresponding SDs of n = 4 independent biological replicates are depicted; the numerical values are given in Supplementary Tables S28 and S29. (**E**,**F**): Similar analysis as in (**C**,**D**) but in untreated (“−EMT”) or treated (“+EMT”) cells. The corresponding statistical values are given in Supplementary Table S30. CI = cell index; AU = arbitrary unit; ns = not significant; * = *p* < 0.05 (statistically significant). The diagrams were created with GraphPad Prism.

Representative raw data and the corresponding regression model are shown in Figure 9A,B. The cell index (CI), given in an arbitrary unit, correlates with the number of adherent cells. As the area of the growth plates was limited, the growth curve reached an asymptote. A non-linear regression with exponential plateau was used to model this growth behavior (Figure 9A right side and Figure 9B):

(1) $$Y=Y_{M}-\left(Y_{M}-Y_{0}\right)*e^{-kx}$$

*Y*_0_ represents the ordinate intersection (it was set to 0 because all experiments started with the same number of cells); *Y_M_* is the upper bound/maximum CI (corresponding to the final cell number). *k* is the rate constant, describing how quickly the cell number reaches *Y_M_* and thus reflects the relative proliferation speed.

Among all cell types, FIB cells and their derivatives exhibited the highest *k*-values, indicating the most rapid proliferation. GK EtOH cells also proliferated faster than GK cells. *k*-values were remarkably lower for EPI cells and their derivatives (Figure 9C, Supplementary Table S28). Analysis of *Y_M_* did not reveal any statistically significant differences between GK cells and their derivatives (Figure 9D, Supplementary Table S29). In contrast, EPI cells exhibited the highest *Y_M_*-value, indicating the highest final cell number and cell density (*p* < 0.05 to all other cell lines). The *Y_M_*-values of FIB cells and their derivatives were comparable to those of GK cells.

Treatment with the EMT-inducing cocktail did not significantly alter *k*-values, although a slight increase was observed for FIB and FIB EtOH Ko cells and a slight decrease for FIB EtOH cells (Figure 9E, Supplementary Table S30). The latter finding reflected the atypical behavior of FIB EtOH cells already observed in the ROS and L-lactate assays. *Y_M_*-values significantly increased in GK cells and their derivatives, while FIB EtOH cells again displayed a paradoxical decrease (Figure 9F, Supplementary Table S30).

In summary, RTCA revealed distinct proliferation kinetics across the different cell lines. GK and FIB cells and their derivatives exhibited similar proliferation kinetics, whereas EPI cells and their derivatives proliferated more slowly but achieved higher final cell densities. These results were consistent with the growth pattern observed by light microscopy (see Figure 1). Supplementary Figure S27 A schematically summarizes the findings concerning cellular proliferation behavior.

### 3.10. EMT Induction Enhanced Cell Migration in Most Cell Lines but Inhibited Migration in FIB EtOH Cells

**Figure 10:**
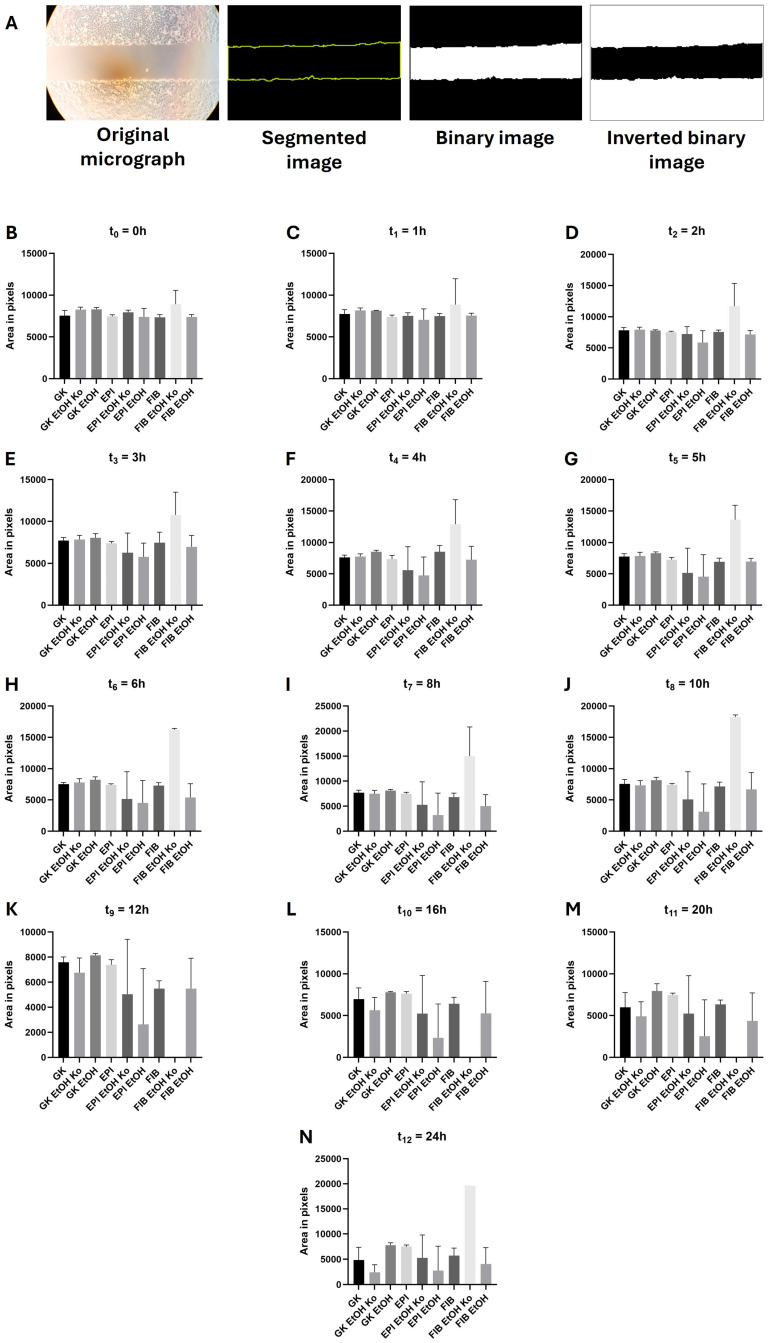
Quantitative analysis of cell migration using the Ibidi^®^ Culture-insert 2 Well 24-System. (**A**): Workflow of image analysis. Light micrographs were segmented with the help of the SAM-2 algorithm, converted to binary images and subsequently inverted. The black area corresponding to the cell-free area was quantified. (**B**–**N**): The migratory behavior of GK, EPI and FIB cells, as well as their derivatives, was analyzed at 13 points in time. Mean values from n = 3 biological replicates and corresponding SDs are shown; the statistical values are listed in Supplementary Tables S31–S43. High pixel counts correspond to a low migratory behavior. For panels (**K**–**M**), FIB EtOH Ko values could not be determined due to technical constraints. The diagrams were created with GraphPad Prism.

Cell migration was analyzed using the Ibidi^®^ Culture-insert 2 Well 24-System, which enables the standardized quantification of cellular migration without mechanical scratching/wounding [61]. The workflow for image processing and analysis is illustrated in Figure 10A. Light micrographs were automatically segmented using the SAM-2 algorithm (with manual visual control), converted to binary images and subsequently inverted. The black pixels represented the cell-free area and were counted. Hence, a high pixel count corresponded to a low migratory behavior. Statistical results are listed in Supplementary Tables S31–S44.

At t_0_ = 0 h, all cell lines exhibited comparable cell-free areas, confirming consistent experimental conditions (Figure 10B). After 2 h, the first differences emerged (Figure 10D). FIB EtOH Ko cells displayed no measurable migration throughout the experiment, resulting in significant differences compared to all other cell lines. No further significant differences were observed in this experimental setup.

**Figure 11:**
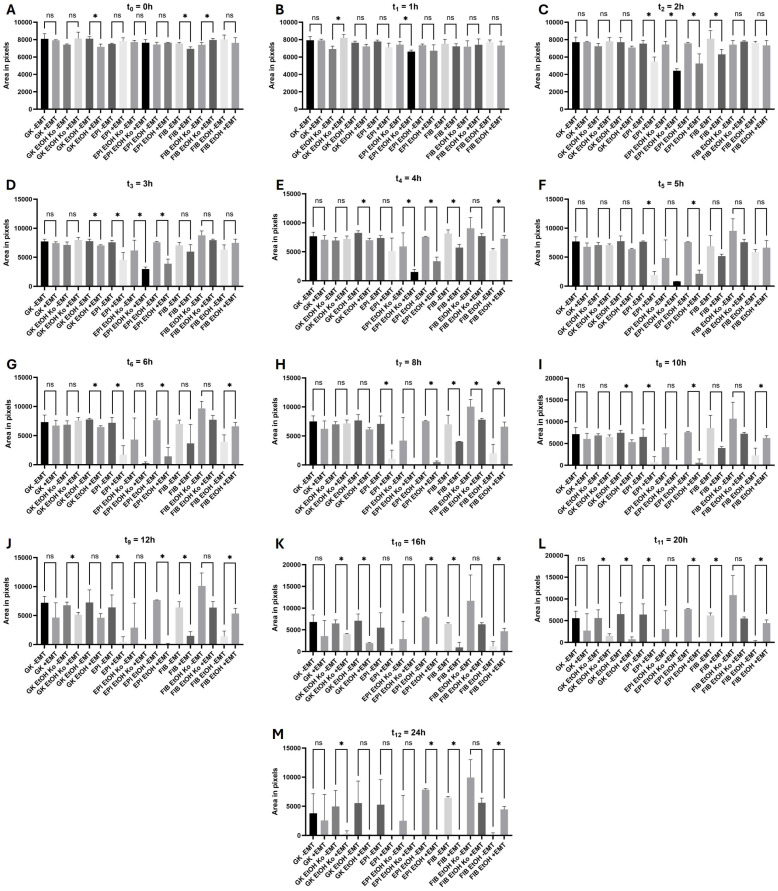
Quantitative analysis of cell migration in GK, EPI and FIB cells and their derivatives in response to treatment with the EMT-inducing cocktail. Cells were analyzed using the Ibidi^®^ Culture-insert 2 Well 24-System. The diagrams depict the mean cell-free area in pixels of n = 3 independent biological experiments with the corresponding SDs. High pixel counts correspond to a low migratory behavior. “−EMT” = untreated cells, “+EMT” = cells exposed to the EMT-inducing cocktail. Statistical values are listed in Supplementary Table S44. (**A**): t_0_ = 0 h, (**B**): t_1_ = 1 h, (**C**): t_2_ = 2 h, (**D**): t_3_ = 3 h, (**E**): t_4_ = 4 h, (**F**): t_5_ = 5 h, (**G**): t_6_ = 6 h, (**H**): t_7_ = 8 h, (**I**): t_8_ = 10 h, (**J**): t_9_ = 12 h, (**K**): t_10_ = 16 h, (**L**): t_11_ = 20 h and (**M**): t_12_ = 24 h. ns = not significant; * = *p* < 0.05 (statistically significant). The diagrams were created with GraphPad Prism.

Biochemical EMT induction markedly enhanced migration in nearly all cell lines (Figure 11, Supplementary Table S44). The strongest responses were detected in EPI cells and their derivatives. The time-resolved analysis shown in Supplementary Figure S27B best reveals the accelerated migratory behavior under “+EMT” conditions. In contrast, FIB EtOH cells exhibited a statistically significant reduction in migration beginning at 4 h (Figure 11E). This finding paralleled their distinct responses in the ROS, L-lactate and RTCA assays.

In summary, EtOH treatment alone had minor effects on cell migration, whereas biochemical EMT induction substantially enhanced cell motility in most cell lines. The inhibitory effect of the EMT cocktail on FIB EtOH cells—despite their fibroblastoid morphology—indicates a unique migratory phenotype within this experimental model.

## 4. Discussion

Despite decades of research, the early molecular and cellular mechanisms driving EtOH-induced OSCCs remain poorly defined. A major limitation has been the lack of robust and reproducible in vitro models that accurately recapitulate the multistep process of noxa-induced oral carcinogenesis. Most available data on OSCC biology have emerged from animal experiments, histological analyses of patient specimens or fully malignant OSCC cell lines derived from advanced tumors or metastases [62,63]. Classical hamster models failed to demonstrate a direct carcinogenic role of EtOH, suggesting rather a co-carcinogenic or tumor-promoting effect [64]. In mice, only a single reproducible EtOH-related OSCC carcinogenesis protocol has been published, using 4-nitroquinoline-1-oxide-induced lesions followed by EtOH exposure. The initial tumor-initiating effects of EtOH could therefore not be studied [65]. Tissue-engineered 3D in vitro models have captured morphological hallmarks such as carcinoma in situ or early invasiveness, yet they rely on fully malignant human OSCC cell lines and thus fail to represent the actual molecular onset of transformation [66]. These research gaps underscore the urgent need for a simple, clinically relevant in vitro model for the study of early EtOH-induced OSCC development.

To date, the GK/EPI/FIB system, originally established by Chamulitrat and colleagues, represents the only well-characterized model simulating the progressive EtOH-induced transformation of human gingival keratinocytes [30]. In this system, primary human gingival keratinocytes were immortalized with the E6 and E7 coding regions of human papillomavirus type 16 (HPV16) to enable long-term in vitro propagation in cell culture. It is important to note that immortalization represents the earliest event in cell transformation, which does not, however, render cells tumorigenic in vivo [67,68,69]. GK cells are morphologically similar to primary human gingival keratinocytes and possess a comparable molecular differentiation, as underscored by their ability to form proper gingival epithelia in vitro without signs of dysplasia or invasion, the expression of *E-Cadherin* and the lack of significant *Vimentin* expression. Chronic in vitro exposure to EtOH led to the establishment of two phenotypically distinct cell populations in GK cultures, namely EPI and FIB, which represent progressive transformation stages. EPI cells are characterized by an epithelioid morphology, an increased nuclear–cytoplasmic ratio and the formation of disorganized epithelia [30,32,33]. FIB cells morphologically resemble fibroblasts and show molecular characteristics of advanced cellular transformation, including reduced contact inhibition, *anoikis*-resistance and increased *Vimentin* expression. EPI and FIB cells remain non-tumorigenic in the *nude*-mouse model [30,32,33].

The molecular and phenotypic changes observed in EPI and FIB cells are in accordance with early EMT. EMT is a central cell biological program characterized by a sequence of molecular events that enable epithelial cells to acquire properties of mesenchymal cells like cell motility and invasiveness. Extracellular signaling molecules like transforming growth factor β1 (TGF-β1), wingless/int-1 5A (WNT-5A), fibroblast growth factors (FGFs) and epidermal growth factors (EGFs) induce EMT [70,71,72,73,74,75]. Intracellularly, the process is coordinated by several key TFs such as Snail, Slug, Twist, POU5F1/Oct4 and ZEB1 [36,76,77]. Phenotypically, these changes can, amongst others, be detected by a gain in mesenchymal Vimentin and a “Cadherin switch” from epithelial E-Cadherin to mesenchymal N-Cadherin [36,37].

From a mechanobiological point of view, AJs and FAs serve as dynamic sensors of mechanical stress, coordinating cellular adhesion with cytoskeletal remodeling and transcriptional activity. Their disruption is a critical step in the initiation of EMT. The above-described Cadherin switch has been shown to be a very early event in OSCC development [78,79,80,81,82]. It weakens cell–cell junctions and liberates β-catenin, which can translocate to the nucleus to activate Wnt-dependent transcriptional programs that confer stem cell–like traits [83,84,85,86]. These observations are supported by pathohistological studies on dysplastic, i.e., premalignant, oral lesions, where a higher degree of cellular atypia and histological dysplasia is linked to an increase in nuclear β-Catenin, presumably through the action of Wnt signaling [87]. Similarly, changes in FA dynamics, including the activation of FAK, promote actin reorganization and facilitate migration [88,89,90,91,92]. The Hippo pathway regulates and integrates these mechanical cues, regulating cell proliferation through the activity of its co-transcriptional activators YAP1, its paralogue TAZ and the TFs TEAD1-4 [93,94,95]. Dysregulated YAP/TAZ signaling has been observed in premalignant oral lesions and correlates with poor prognosis in OSCCs [96,97,98]. Although numerous studies have described the individual role of these signaling pathways in OSCCs and thus underscore the central role of the mechanobiological signaling axes for EMT and cellular transformation in OSCC development, they have never been studied in parallel in a single gingival cell culture system employing chronic EtOH exposure.

Our study thus addressed several as-yet unresolved molecular aspects of the GK/EPI/FIB cell culture model in this context [30].

First, we demonstrated that long-term EtOH exposure generates previously unrecognized intermediate cell phenotypes, i.e., EPI EtOH and FIB EtOH cells, reflecting new transitional stages in the continuum of very early in vitro oral carcinogenesis. This supports the concept of a progressive, stepwise cellular transformation process that can be reliably modeled by stable cell lines. We acknowledge, however, that our 2D culture system neither pictures the transition to a completely malignant cellular phenotype nor fully mimics in vivo tumorigenesis and lacks critical elements such as tumor–stroma interactions and immune signaling.

Second, we established a straightforward closed-culture system that enables continuous EtOH exposure under bicarbonate-free buffering. This setup addresses a long-standing limitation in EtOH toxicity research [39,99,100]. Physicochemical modeling confirmed the stability of EtOH concentrations, while the resulting partial hypoxia induced metabolic shifts, reflected by increased anaerobic glycolysis and L-lactate accumulation. GK EtOH Ko, EPI EtOH Ko and FIB EtOH Ko cells thus exhibited features of early “metabolic reprogramming” that persisted for multiple passages, likely mediated by hypoxia-inducible factors and rapid epigenetic adaptation [101,102].

Third, this study demonstrates for the first time that non-malignant, EtOH-treated gingival keratinocytes respond to biochemical EMT induction. Previously, the EMT cocktail used here was primarily applied to fully malignant carcinoma cell lines [103,104,105,106]. The molecular and morphological responsiveness of our cells to this cocktail underscores a continuous, progressive shift from benign toward premalignant cell phenotypes in OSCC development. It also suggests that EMT-related signaling may already be active during the earliest, premalignant stages of EtOH-induced cell transformation in the oral cavity.

**Figure 12:**
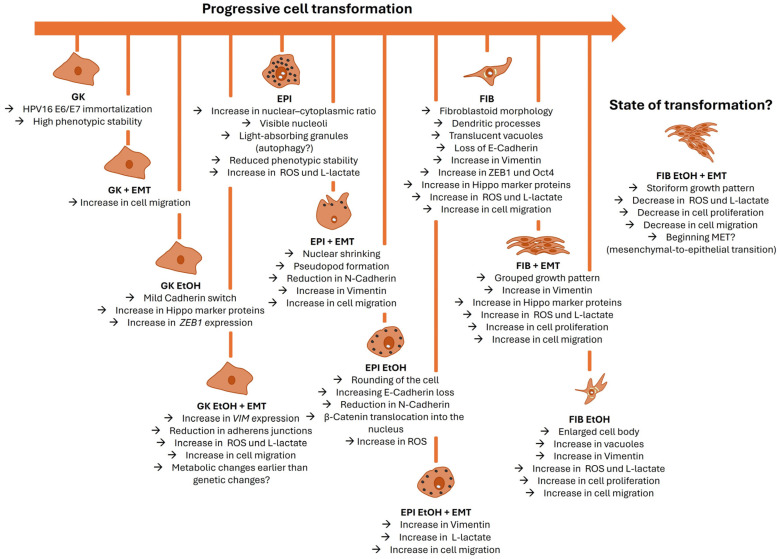
Stepwise cellular transformation of GK, EPI and FIB cells under EtOH and “+EMT”-stimulation. From left to right, the scheme depicts progression from GK (immortalized parental cells) to EPI (epithelial phenotype) and FIB (fibroblastoid/mesenchymal phenotype). For each condition (EtOH, +EMT, EtOH + EMT), characteristic changes in morphology, marker expression, metabolism and cell behavior are summarized. Hallmark alterations across the axis include decreased E-cadherin/CDH1, increased Vimentin, elevated Hippo pathway proteins, higher ROS and L-lactate levels, cell-shape changes (rounding, dendritic processes) and increased migration and proliferation. In FIB EtOH Ko cells under “+EMT” conditions, features consistent with a beginning MET are observed. EtOH = ethanol; EMT = epithelial-to-mesenchymal transition; MET = mesenchymal-to-epithelial transition; ROS = reactive oxygen species; HPV = human papillomavirus. Created in Biorender. Steinberg, T. (2025) https://BioRender.com/sglefuz.

Together, these findings position the GK/EPI/FIB model system as a unique platform for studying the metabolic, transcriptional and behavioral hallmarks of early EtOH-induced OSCC initiation. The key cell line-specific characteristics are discussed below and summarized in Figure 12 and Supplementary Figure S28.

### 4.1. Morphological and Molecular Properties of GK Cells and Their Derivatives

GK cells and their derivatives represent an early, immortalized but phenotypically stable stage of transformation. Their morphology closely resembles primary gingival keratinocytes and differs fundamentally from established OSCC lines [67,107,108,109]. Consistent with this, GK cells stably expressed AJ markers such as *E-Cadherin*, whose loss represents a hallmark of early dysplasia in the human gingiva [110,111]. The low *Vimentin* expression confirmed their epithelial identity and differentiation [112]. Nevertheless, GK EtOH cells displayed subtle increases in *CDH2* and *ZEB1* transcripts as well as Hippo pathway components, suggesting the onset of an EMT-like state. Accordingly, the literature provides evidence for the early role of ZEB1 and YAP1 in OSCC progression [113].

The EMT cocktail exerted only minor transcriptional effects on GK cells and their derivatives but increased ROS and L-lactate levels, indicating a metabolically responsive phenotype [102,114].

Functionally, EtOH treatment modestly enhanced GK proliferation but did not affect migration, whereas the EMT cocktail significantly increased both proliferation and migratory capacity. These behavioral changes reflect a biological trade-off between proliferation and motility typical of EMT progression: cells prioritize migration over division during invasion into neighboring tissues. Later, when cells form metastases at distant sites (“seed-and-soil-theory”), they undergo mesenchymal-to-epithelial transition (MET; the opposite process to EMT), restart proliferation and form micro- and macro-metastases [115,116]. Mouse models of squamous cell carcinomas support this inverse correlation of EMT marker expression and proliferative behavior, which underscores the validity of our cell culture model [116].

In summary, GK cells remain phenotypically stable and express characteristic epithelial markers, yet biochemical EMT induction reprograms their metabolism and behavior. This suggests that immortalization sensitizes cells to metabolic cues capable of initiating EMT. The data further imply that the earliest EtOH-driven events in OSCC are predominantly metabolic and behavioral rather than genomic—echoing the long-standing debate of whether genetic mutations or metabolic rewiring initiate carcinogenesis [117].

### 4.2. EPI Cells and Their Derivatives Represent Intermediate Stages of Cell Transformation

EPI, EPI EtOH Ko and EPI EtOH cells display more advanced transformation features than GK cells and their derivatives. Morphologically, they exhibit polygonal or rounded cell shapes with an elevated nuclear–cytoplasmic ratio and prominent nucleoli—classical indicators of malignant progression, which are also detected in diverse human OSCC cell lines [42,118]. Cytoplasmic granules may represent autophagic vacuoles, consistent with EtOH-induced autophagy in other tissues [119,120]. Chronic EtOH exposure produced the distinct EPI EtOH phenotype with reduced cell–cell adhesion and the loss of cobblestone morphology, consistent with literature on EtOH-induced membrane alterations [121,122,123]. Of note, new artificial intelligence-based models underscore the pivotal interrelationship of cell morphology and function, reviving the old principle of “form follows function” in the context of cell transformation [124].

AJ analysis revealed a marked loss of E-Cadherin in EPI EtOH cells, paralleled by a nuclear redistribution of β-Catenin, signifying a functional shift from adhesion to transcriptional regulation. In vivo, this is equivalent to the molecular processes leading to oral dysplasia [125,126].

*Vimentin* expression and filament stabilization increased after experimental EMT induction, supporting progressive mesenchymal transition. Interestingly, both *CDH1* and *CDH2* declined simultaneously—a deviation from the classical “Cadherin switch” model in the context of EMT [127,128]. However, similar paradoxical patterns were previously observed in rare gastrointestinal and gynecologic malignancies [129,130].

Metabolically, EPI cells exhibited elevated basal ROS levels compared with GK cells, with EPI EtOH showing further increases. The EMT cocktail enhanced L-lactate production in EPI EtOH cells, confirming metabolic responsiveness at a more advanced stage. While GK cells reacted to the treatments mainly at the metabolic and behavioral level, EPI cells responded primarily on transcriptional and protein levels—consistent with their intermediate transformation status [131].

Cell proliferation was largely unaffected by either treatment, but biochemical EMT induction markedly enhanced cell migration, particularly in EtOH-pretreated cells. This indicates that chronic EtOH exposure sensitizes epithelial cells to EMT-inducing signals, which promote motility and plasticity.

Taken together, EPI and EPI EtOH cells exhibited morphological changes that can be attributed to chronic EtOH treatment. Most treatment effects could be detected on the transcriptional or protein level, especially in the context of AJs. Thus, long-term EtOH treatment renders cells more susceptible to EMT-related molecular changes.

### 4.3. EMT and Beyond: Molecular Features of FIB Cells and Their Derivatives

FIB cells and their derivatives represent the most advanced transformation stage within the GK/EPI/FIB system. In line with the observed mesenchymal shift in FIB cells, we found in previous studies that key EMT master regulators Snail1, ZEB1 and Twist1 were markedly upregulated in FIB cells compared to both the parental GK and intermediate EPI cells. These transcription factors are well-known drivers of EMT that repress epithelial genes and induce mesenchymal traits. The significant induction of Snail1, ZEB1 and Twist1 in FIB cells strongly supports the notion that chronic EtOH exposure has triggered an EMT program at the transcriptional level, pushing the cells toward a more mesenchymal and thus premalignant phenotype [31].

In the current study, FIB cells and their derivatives display profound further EtOH and “+EMT”-induced remodeling of morphology, organellar composition and growth pattern—especially their fibroblastoid morphology and the numerous perinuclear vacuoles, which are potentially autophagic membranes (see Section 4.2). Sequential EtOH and “+EMT” treatments exerted additive effects, producing a storiform growth pattern reminiscent of sarcomatoid histology [132]. Similar additive effects of external noxae and autocrine EMT signaling have been reported during OSCC progression in vivo [133,134,135].

At the molecular level, AJ disintegration was complete in FIB cells and their derivatives: *CDH1* expression was lost, β-Catenin localized predominantly to nuclei, and EMT master regulators such as *Oct4* and *ZEB1* were strongly upregulated. The sequential activation of ZEB1 following E-Cadherin loss aligns with canonical EMT progression, likely mediated by feed-forward loops where ZEB1 and Twist repress *CDH1* to stabilize mesenchymal traits [136,137,138].

FIB cells also exhibited the highest Vimentin levels and enhanced Hippo signaling. Nuclear YAP1 accumulation, previously linked to transformation in this model, was further promoted by EMT induction—consistent with in vivo data highlighting YAP1 as a key OSCC driver [78,98,139,140,141].

Metabolically, FIB and especially FIB EtOH cells displayed pronounced ROS and L-lactate production—important hallmarks of metabolic reprogramming associated with aggressive OSCC phenotypes [142,143].

Intriguingly, “+EMT” treatment reversed these trends in FIB EtOH cells, reducing ROS and L-lactate levels, proliferation rate and migration. This paradoxical response may represent a partial mesenchymal-to-epithelial transition (MET), a process observed during metastatic colonization. The concurrent upregulation of *CDH1* and downregulation of *YAP1* in FIB EtOH cells under “+EMT” conditions support this interpretation [37]. Alternatively, the combined noxa exposure may have pushed FIB EtOH cells toward metabolic exhaustion and loss of phenotypic stability, representing an evolutionary “dead-end” incapable of full malignant conversion [144].

Collectively, FIB cells have undergone partial EMT yet remain non-tumorigenic in vivo. The precise transformation state of FIB EtOH cells requires further investigation but may represent a critical transitional node between premalignant and malignant phenotypes.

## 5. Conclusions

This study provides novel insights into the early stages of EtOH-induced oral carcinogenesis and establishes an updated and enhanced GK/EPI/FIB platform for studying stepwise cell transformation. Continuous EtOH exposure and biochemical EMT induction revealed that metabolic reprogramming, redox imbalance and junctional remodeling precede stable genetic transformation.

The data highlight three key concepts:

(1) Immortalization primes epithelial cells for metabolic and behavioral responsiveness to microenvironmental cues such as EtOH or EMT-inducing factors;
(2) Chronic EtOH exposure sensitizes cells to EMT signaling, which in turn drives morphological and functional cell plasticity;
(3) Advanced transformation stages (especially FIB EtOH) exhibit metabolic heterogeneity and potentially even partial MET, reflecting the dynamic equilibrium of epithelial and mesenchymal cell differentiation.

Beyond elucidating fundamental mechanisms, this model paves the way for translational studies aiming at identifying early biomarkers of EtOH-related OSCC development. Future work will extend these findings to 3D co-culture systems and transcriptomic profiling to define diagnostic and preventive targets for EtOH-associated oral malignancies.

## Data Availability

The original data presented in the study are openly available in Zenodo at DOI 10.5281/zenodo.17541823.

## Supplementary Materials

The following supporting information can be downloaded at: https://www.mdpi.com/article/10.3390/cells14231887/s1, Supplementary Materials and Methods with Supplementary Tables S.M1–S.M3; Supplementary Results with Figures S1–S28 and Tables S1–S44.

## Abbreviations

The following abbreviations are used in this manuscript:

AA	Acetaldehyde
ACTB	β-Actin
ADH	Alcohol dehydrogenase
AJ	Adherens junction
ALDH	Aldehyde dehydrogenases
ANOVA	Analysis of variance
CDH1	E-Cadherin
Ct	Cycle threshold
DCFDA	Dichlorodihydrofluorescein-diacetate
DKK-1	Dickkopf-related protein 1
DTT	Dithiothreitol
ECL	Enhanced chemiluminescence
ECM	Extracellular matrix
EGF	Epidermal growth factor
EMT	Epithelial-to-mesenchymal transition
EtOH	Ethanol
FA	Focal adhesion
FAK	Focal adhesion kinase
FCS	Fetal calf serum
FGF	Fibroblast growth factor
GAPDH	Glyceraldehyde 3-phosphate dehydrogenase
GOI	Gene of interest
H2DCFDA	2’,7’-Dichlorodihydrofluorescein-diacetate
HPV16	Human papillomavirus type 16
IACR	International Agency for Research on Cancer
IIF	Indirect immunofluorescence
LDH	Lactate dehydrogenase
MET	Mesenchymal-to-epithelial transition
NF2	Neurofibromatosis type 2
NRT	No reverse transcript control
NTC	No template control
OSCC	Oral squamous cell carcinoma
PBS	Phosphate-buffered saline
PVDF	Polyvinylidene fluoride
qPCR	Quantitative polymerase chain reaction
ROS	Reactive oxygen species
RPL13A	60S ribosomal protein L13a
RT	Room temperature
RTCA	Real-time cell analyzer
SDS-PAGE	Sodium dodecyl sulfate-polyacrylamide gel electrophoresis
sFRP-1	Soluble Frizzled-Related Protein 1
TAZ	Transcriptional co-activator with PDZ-binding motif
TBCB	β-Tubulin
TBHP	Tert-Butyl hydroperoxide
TBS	TRIS-buffered saline
TBST	TBS with Tween^®^ 20
TEAD1-4	TEA domain family members 1-4
TGF-β1	Transforming growth factor β1
TRIS	Tris(hydroxymethyl)aminomethane
WNT-5A	Wingless/int-1 5A
YAP1	Yes-associated protein 1
ZEB1	homologue zinc finger E-box-binding homeobox 1
