# Supplementary material for: Chronic Ethanol Exposure Induces Early Epithelial-to-Mesenchymal Transition (EMT) and Premalignant Changes in Gingival Keratinocytes: An In Vitro Model of Very Early Oral Carcinogenesis
Source: Cells. 2025 Nov 27;14(23):1887. doi: 10.3390/cells14231887 (PMC12691217; doi:10.3390/cells14231887)
Supplement: Supplementary file 1 [file cells-14-01887-s001.zip › cells-3997528-supplementary.pdf]

cells

MDPI

Supplementary Material and Methods

Supplementary Table S.M1 Overview of cell lines and passages used in this study

<table>
<tr><th>Name and passage numbers of cell lines</th><th>Culture medium</th><th>characteristics</th></tr>
<tr><td>GK
P# 37-76</td><td rowspan="3">KGM-2 medium (PromoCell, Heidelberg, Germany)

2,46% [v/v] KGM-2 Supplement mix (PromoCell)
0,06 mM $CaCl_2$ (PromoCell)

0,1 mg/mL Kanamycin (Sigma-Aldrich, St. Louis, MI, USA)</td><td>Parental cell line; immortalized with open reading frames of E6 and E7 of HPV16 (see [30])</td></tr>
<tr><td>GK EtOH Ko
P# 52-82</td><td>Newly established cell line (derivative of GK), which was cultured in closed culture flasks for two months; afterwards it was retransferred to an open, bicarbonate-containing buffer system (see chapter 2.2)</td></tr>
<tr><td>GK EtOH
P# 51-81</td><td>Newly established cell line (derivative of GK), which was cultured in closed culture flasks for two months with continuous EtOH exposure; afterwards it was retransferred to an open, bicarbonate-containing buffer system (see chapter 2.2)</td></tr>
<tr><td>EPI
P# 142-199</td><td rowspan="6">DMEM (low glucose, HEPES, pyruvate; Life Technologies GmbH)

10% [v/v] FCS (Bio & Sell GmbH, Feucht, Germany)

1% [v/v] GlutaMAX™ corresponding to 2 mM L-alanyl-L-glutamin (Thermo Fisher Scientific, Waltham, MA, UA)</td><td>Already established, epitheloid derivative of GK, which was established after continuous EtOH exposure (see [30])</td></tr>
<tr><td>EPI EtOH Ko
P# 155-162</td><td>Newly established cell line (derivative of EPI), which was cultured in closed culture flasks for two months; afterwards it was retransferred to an open, bicarbonate-containing buffer system (see chapter 2.2)</td></tr>
<tr><td>EPI EtOH
P# 145-223</td><td>Newly established cell line (derivative of EPI), which was cultured in closed culture flasks for two months with continuous EtOH exposure; afterwards it was retransferred to an open, bicarbonate-containing buffer system (see chapter 2.2)</td></tr>
<tr><td>FIB
P# 19-49</td><td>Already established, fibroblastoid derivative of GK, which was established after continuous EtOH exposure (see [30])</td></tr>
<tr><td>FIB EtOH Ko
P# 28-55</td><td>Newly established cell line (derivative of FIB), which was cultured in closed culture flasks for two months; afterwards it was retransferred to an open, bicarbonate-containing buffer system (see chapter 2.2)</td></tr>
<tr><td>FIB EtOH
P# 25-59</td><td>Newly established cell line (derivative of FIB), which was cultured in closed culture flasks for two months with continuous EtOH exposure; afterwards it was retransferred to an open, bicarbonate-containing buffer system (see chapter 2.2)</td></tr>
</table>

	0,1 mg/mL Kanamycin	

Physiocochemical Modeling of EtOH-treatment

A simple physicochemical modeling of the culture system was developed, assuming a small primary EtOH metabolism in our cell lines and thus making use of the Antoine equation and the ideal gas equation [102].

Vapor pressure of EtOH at 37°C is approximately 15.2347 kPa = $15.2347 \cdot 10^3 \frac{kg}{m \cdot s^2}$. Assuming the behavior of an ideal gas, Equation S1 can be applied:

$$p \cdot V = n \cdot R \cdot T \quad \text{(Equation S1)}$$

p = gas pressure; V = gas volume; n = amount of substance; R = ideal gas constant with approximately 8.314 $\frac{J}{mol \cdot K}$ (which equals 8.314 $\frac{kg \cdot m^2}{s^2 \cdot mol \cdot K}$) and T = Temperature (in Kelvin). The maximum volume of the 25 cm² culture flasks is 50 mL (according to the manufacturer), which overestimates the maximum gas volume in the flask when using 6 mL of medium. Therefore, it can be estimated:

$$n = \frac{p \cdot V}{R \cdot T} = \frac{15.2347 \cdot 10^3 \frac{kg}{m \cdot s^2} \cdot 5.00 \cdot 10^{-5}\ m^3}{8.314 \frac{kg \cdot m^2}{s^2 \cdot mol \cdot K} \cdot 310.00\ K} = 2.96 \cdot 10^{-7} mol \quad \text{(Equation S2)}$$

If the cell culture medium is regarded as a water and EtOH (86 mM) solution, the percentage of substance amount of EtOH can be calculated as:

$$\frac{c_{Ethanol}}{c_{Water}} = \frac{86.0 \cdot 10^{-3} \frac{mol}{L}}{55.5 \frac{mol}{L}} = 1.6 \cdot 10^{-3} \quad \text{(Equation S3)}$$

$c_{Ethanol}$ refers to the concentration of EtOH; c_{Water} to the concentration of water.
If it is assumed that Raoult´s law applies to the system, multiplication of equations 2 and 3 equals:

$$n_{EtOH,Gas} = 1.6 \cdot 10^{-3} \cdot 2.96 \cdot 10^{-7} mol = 4.6 \cdot 10^{-10} mol \quad \text{(Equation S4)}$$

$n_{EtOH,Gas}$ is the maximum amount of EtOH that can be dissolved in the gas phase of the 25 cm² culture flask. As there are only 6 mL of medium within the flasks, this means:

$$n_{EtOH,Medium} = c_{Ethanol} \cdot V_{Medium} = 86.0 \cdot 10^{-3} \frac{mol}{L} \cdot 6.0 \cdot 10^{-6}\ m^3 = 5.2 \cdot 10^{-7} mol \quad \text{(Equation S5)}$$

The quotient of equations 4 and 5 gives:

$$\frac{n_{EtOH,Gas}}{n_{EtOH,Medium}} = \frac{4.6 \cdot 10^{-10} mol}{5.2 \cdot 10^{-7} mol} = 8.9 \cdot 10^{-4} \quad \text{(Equation S6)}$$

$8.9 \cdot 10^{-4}$ is the proportion of EtOH evaporating into the gas phase as approximated by these calculations, which equals less than 1‰ and can therefore be neglected.

Supplementary Table S.M2 Overview of Primers used in this study

Primer (Gene of interest)	*Manufacturer and Gene Reference ID*
RT² qPCR Primer Assay for Human ACTB	*Qiagen, Hilden, Germany; GeneGlobe ID - PPH00073G; RefSeq# NM_001101*
RT² qPCR Primer Assay for Human CDH1	*Qiagen, Hilden, Germany; GeneGlobe ID - PPH00135F; RefSeq# NM_004360*
RT² qPCR Primer Assay for Human CDH2	*Qiagen, Hilden, Germany; GeneGlobe ID - PPH00636F; RefSeq# NM_001792*
RT² qPCR Primer Assay for Human CTNNB1	*Qiagen, Hilden, Germany; GeneGlobe ID - PPH00643F; RefSeq# NM_001904*
RT² qPCR Primer Assay for Human GAPDH	*Qiagen, Hilden, Germany; GeneGlobe ID - PPH00150F; RefSeq# NM_002046*
RT² qPCR Primer Assay for Human ITGB1	*Qiagen, Hilden, Germany; GeneGlobe ID - PPH00650B; RefSeq# NM_002211*
RT² qPCR Primer Assay for Human NF2	*Qiagen, Hilden, Germany; GeneGlobe ID - PPH00203A; RefSeq# NM_000268*
RT² qPCR Primer Assay for Human POU5F1	*Qiagen, Hilden, Germany; GeneGlobe ID - PPH02394E; RefSeq# NM_002701*
RT² qPCR Primer Assay for Human PTK2	*Qiagen, Hilden, Germany; GeneGlobe ID - PPH02827A; RefSeq# NM_005607*

RT² qPCR Primer Assay for Human RPL13A	*Qiagen, Hilden, Germany; GeneGlobe ID - PPH01020B; RefSeq# NM_012423*
RT² qPCR Primer Assay for Human TAZ	*Qiagen, Hilden, Germany; GeneGlobe ID - PPH60066A; RefSeq# NM_000116*
RT² qPCR Primer Assay for Human TBCB	*Qiagen, Hilden, Germany; GeneGlobe ID - PPH08908A; RefSeq# NM_001281*
RT² qPCR Primer Assay for Human TEAD1	*Qiagen, Hilden, Germany; GeneGlobe ID - PPH02161C; RefSeq# NM_021961*
RT² qPCR Primer Assay for Human VIM	*Qiagen, Hilden, Germany; GeneGlobe ID - PPH00417F; RefSeq# NM_003380*
RT² qPCR Primer Assay for Human YAP1	*Qiagen, Hilden, Germany; GeneGlobe ID - PPH13459A; RefSeq# NM_006106*
RT² qPCR Primer Assay for Human ZEB1	*Qiagen, Hilden, Germany; GeneGlobe ID - PPH01922A; RefSeq# NM_030751*

Supplementary Table S.M3 Overview of primary and secondary antibodies used for immunodetection in Western Blot (WB) and indirect immunofluorescence (IIF) experiments

**Antibody and species**	**Application and dilution**	**Reference number and manufacturer**
Alexa Fluor 488™ Goat anti-Mouse IgG	IIF (1:200 in 2% BSA/PBS)	A11029, Invitrogen, Waltham, MA, USA
Alexa Fluor 488™ Goat anti-Rabbit IgG	IIF (1:200 in 2% BSA/PBS)	A11008, Invitrogen, Waltham, MA, USA
Alexa Fluor 594™ Goat anti-Mouse IgG	IIF (1:200 in 2% BSA/PBS)	A11005, Invitrogen, Waltham, MA, USA
Alexa Fluor 594™ Goat anti-Rabbit IgG	IIF (1:200 in 2% BSA/PBS)	A11012, Invitrogen, Waltham, MA, USA
E-Cadherin (Mouse)	WB 1:2.500 in 0.5% BSA/TBST), IIF (1:100 in 2% BSA/PBS)	ab76055, Abcam, Cambridge, UK
HRP Goat anti-Rabbit (H + L),<br>WesternSure® secondary antibody	WB (1:5,000 in 0.5% BSA/TBST)	926-80011, Li-cor Biosciences GmbH, Bad Homburg vor der Höhe, Germany
HRP Goat anti-Mouse (H + L),<br>WesternSure® secondary antibody	WB (1:5,000 in 0.5% BSA/TBST)	926-80010, Li-cor Biosciences GmbH, Bad Homburg vor der Höhe, Germany
Integrin β-1 (Mouse)	IIF (1:100 in 2% BSA/PBS)	sc-374429, Santa Cruz Biotechnology, Dallas, TX, USA
Merlin (Rabbit)	WB (1:5,000 in 0.5% BSA/TBST)	ab109244, Abcam, Cambridge, UK
Merlin (Mouse)	IIF (1:100 in 2% BSA/PBS)	ab88957, Abcam, Cambridge, UK
N-Cadherin (Mouse)	WB (1:1,000 in 0.5% BSA/TBST); IIF (1:100 in PBS)	ab98952, Abcam, Cambridge, UK
Oct-4 (Mouse)	WB (1:1,100 in 0.5% BSA/TBST); IIF (1:100 in 2% BSA/PBS)	60242-1-Ig, Proteintech® GmbH, Planegg-Martinsried, Germany
pFAKY397 (Rabbit)	IIF (1:100 in 2% BSA/PBS)	ab81298, Abcam, Cambridge, UK
TAZ (Mouse)	WB (1:1,000 in 0.5% BSA/TBST); IIF (1:100 in 2% BSA/PBS)	sc-293183, Santa Cruz Biotechnology, Dallas, TX, USA
TEAD2 (Rabbit)	WB (1:5,000 in 0.5% BSA/TBST), IIF (1:100 in 2% BSA/PBS)	Pab0961-IP, Covalab S.A.S., Bron, France
Vimentin (Rabbit)	WB (1:3,000 in 0.5% BSA/TBST); IIF (1:100 in 2% BSA/PBS)	ab92547, Abcam, Cambridge, UK
YAP1 (Mouse)	WB (1:1,500 in 0.5% BSA/TBST); IIF (1:100 in 2% BSA/PBS)	sc-376830, Santa Cruz Biotechnology, Dallas, TX, USA
ZEB1 (Mouse)	WB (1:1,000 in 0.5% BSA/TBST); IIF (1:100 in 2% BSA/PBS)	66279-1-Ig, Proteintech® GmbH, Planegg-Martinsried, Germany
β-Actin (Rabbit)	WB (1:5,000 in 0.5% BSA/TBST)	ab8227, Abcam, Cambridge, UK
β-Catenin (Rabbit)	WB (1:2,500 in 0.5% BSA/TBST); IIF (1:100 in 2% BSA/PBS)	ab32572, Abcam, Cambridge, UK
β-Tubulin (Rabbit)	WB (1:5,000 in 0.5% BSA/TBST)	ab6046, Abcam, Cambridge, UK

Supplementary Results

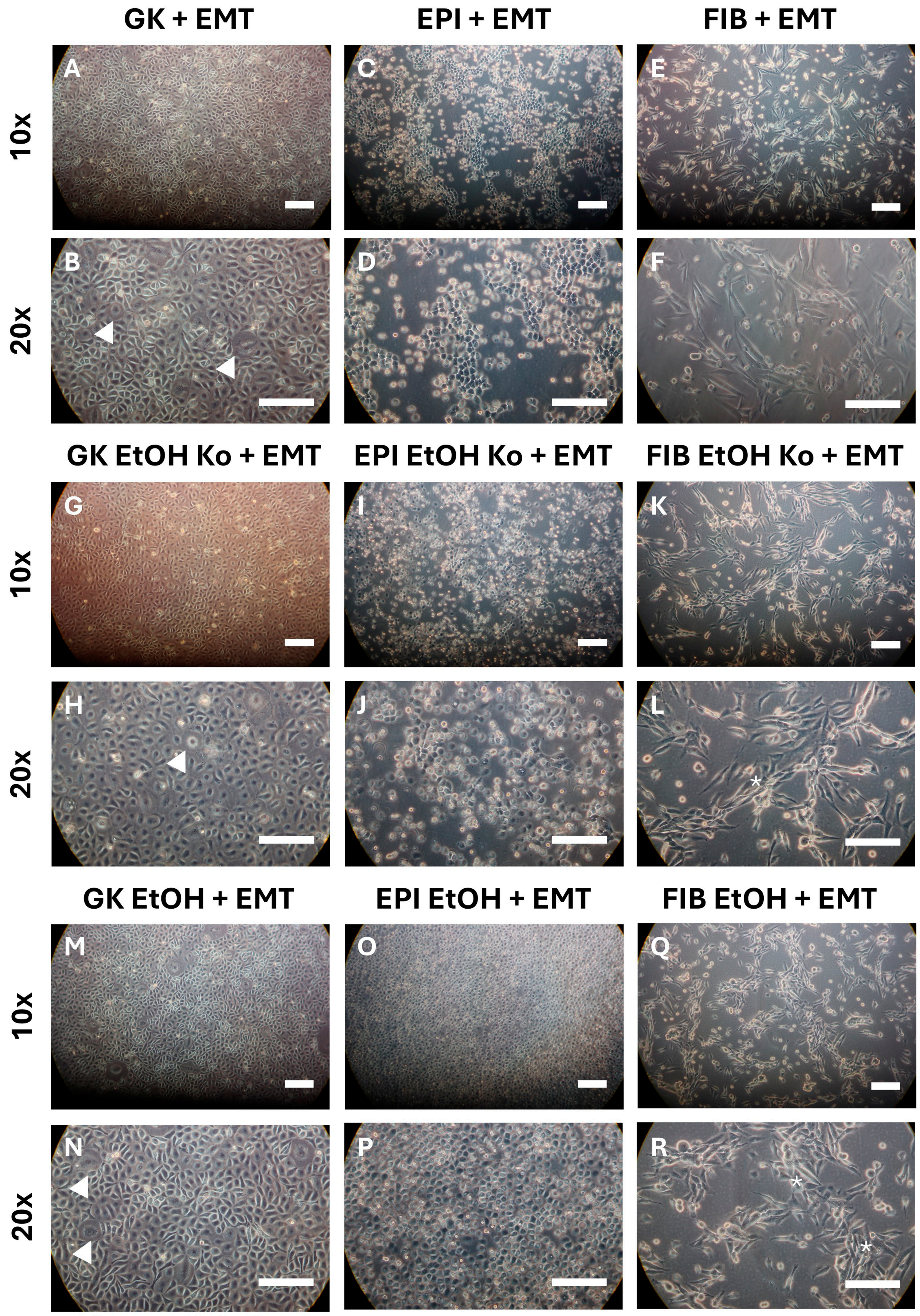

Supplementary Figure S1 EMT cocktail-induced morphological changes in gingival keratinocytes as detected by light microscopy (10x and 20x magnification). A and B: GK cells; C and D: EPI cells; E and F: FIB cells; G and H: GK EtOH Ko cells; I and J: EPI EtOH Ko cells; K and L: FIB EtOH KO cells; M and N: GK EtOH cells; O and P: EPI EtOH cells; Q and R: FIB EtOH cells. Scale bars represent 200 μm.

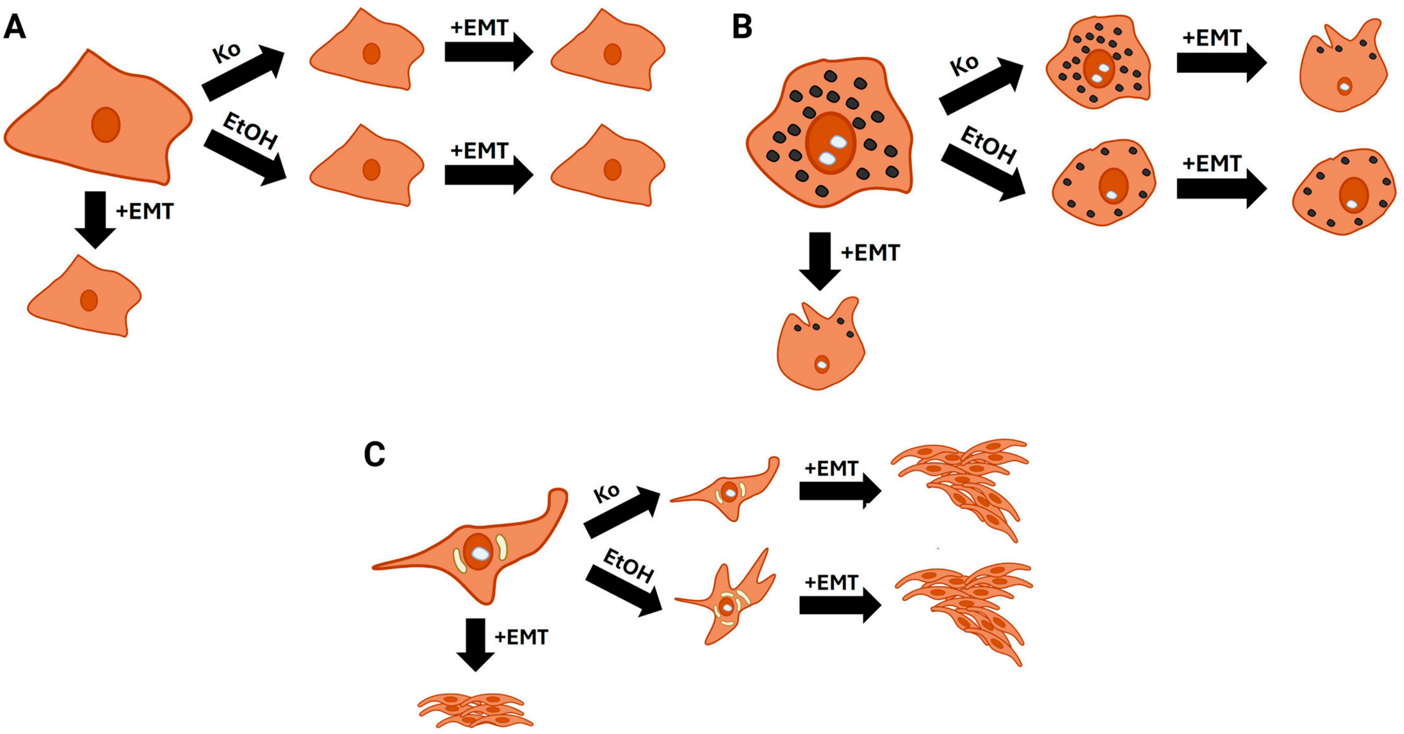

Supplementary Figure S2 Schematic summary of morphological changes induced by EtOH- or "+EMT"-treatment in GK, EPI and FIB cells. For color/symbol legend see Figure 1. A: GK cells and their derivatives. B: EPI cells and their derivatives. C: FIB cells and their derivatives. Created in BioRender. Steinberg, T. (2025) https://BioRender.com/ftesbxb

Supplementary Table S1 Statistical analyses (p-values) concerning the expression of *E-Cadherin/CDH1* in cells without experimental EMT induction (n.s. = not significant)

CDH1	GK	GK EtOH Ko	GK EtOH	EPI	EPI EtOH Ko	EPI EtOH	FIB	FIB EtOH Ko	FIB EtOH
**GK**		n.s.	n.s.	n.s.	n.s.	0.0007	<0.0001	<0.0001	<0.0001
**GK EtOH Ko**	n.s.		n.s.	n.s.	n.s.	<0.0001	<0.0001	<0.0001	<0.0001
**GK EtOH**	n.s.	n.s.		n.s.	n.s.	<0.0001	<0.0001	<0.0001	<0.0001
**EPI**	n.s.	n.s.	n.s.		n.s.	<0.0001	<0.0001	<0.0001	<0.0001
**EPI EtOH Ko**	n.s.	n.s.	n.s.	n.s.		<0.0001	<0.0001	<0.0001	<0.0001
**EPI EtOH**	0.0007	<0.0001	<0.0001	<0.0001	<0.0001		<0.0001	<0.0001	<0.0001
**FIB**	<0.0001	<0.0001	<0.0001	<0.0001	<0.0001	<0.0001		0.0154	n.s.
**FIB EtOH Ko**	<0.0001	<0.0001	<0.0001	<0.0001	<0.0001	<0.0001	0.0154		<0.0001
**FIB EtOH**	<0.0001	<0.0001	<0.0001	<0.0001	<0.0001	<0.0001	n.s.	<0.0001	

Supplementary Table S2 Statistical analyses (p-values) concerning the expression of *N-Cadherin/CDH2* in cells without experimental EMT induction (n.s. = not significant)

CDH2	GK	GK EtOH Ko	GK EtOH	EPI	EPI EtOH Ko	EPI EtOH	FIB	FIB EtOH Ko	FIB EtOH
**GK**		0.0343	0.0013	0.0001	0.0039	0.0003	<0,0001	0.0019	n.s.
**GK EtOH Ko**	0.0343		n.s.	<0,0001	<0,0001	<0,0001	<0,0001	<0,0001	0.0003
**GK EtOH**	0.0013	n.s.		<0,0001	<0,0001	<0,0001	<0,0001	<0,0001	<0,0001
**EPI**	0.0001	<0,0001	<0,0001		n.s.	n.s.	n.s.	n.s.	0.0123
**EPI EtOH Ko**	0.0039	<0,0001	<0,0001	n.s.		n.s.	n.s.	n.s.	n.s.
**EPI EtOH**	0.0003	<0,0001	<0,0001	n.s.	n.s.		n.s.	n.s.	0.0299
**FIB**	<0,0001	<0,0001	<0,0001	n.s.	n.s.	n.s.		n.s.	0.0009
**FIB EtOH Ko**	0.0019	<0,0001	<0,0001	n.s.	n.s.	n.s.	n.s.		n.s.
**FIB EtOH**	n.s.	0.0003	<0,0001	0.0123	n.s.	0.0299	0.0009	n.s.	

Supplementary Table S3 Statistical analyses (p-values) concerning the expression of *β-Catenin*/*CTNNB1* in cells without experimental EMT induction (n.s. = not significant)

CTNNB1	GK	GK EtOH Ko	GK EtOH	EPI	EPI EtOH Ko	EPI EtOH	FIB	FIB EtOH Ko	FIB EtOH
GK		n.s.	0.0085	<0,0001	<0,0001	<0,0001	n.s.	n.s.	n.s.
GK EtOH Ko	n.s.		n.s.	<0,0001	<0,0001	<0,0001	n.s.	n.s.	n.s.
GK EtOH	0.0085	n.s.		<0,0001	<0,0001	<0,0001	n.s.	n.s.	n.s.
EPI	<0,0001	<0,0001	<0,0001		n.s.	n.s.	<0,0001	<0,0001	<0,0001
EPI EtOH Ko	<0,0001	<0,0001	<0,0001	n.s.		n.s.	<0,0001	<0,0001	<0,0001
EPI EtOH	<0,0001	<0,0001	<0,0001	n.s.	n.s.		<0,0001	<0,0001	<0,0001
FIB	n.s.	n.s.	n.s.	<0,0001	<0,0001	<0,0001		n.s.	n.s.
FIB EtOH Ko	n.s.	n.s.	n.s.	<0,0001	<0,0001	<0,0001	n.s.		n.s.
FIB EtOH	n.s.	n.s.	n.s.	<0,0001	<0,0001	<0,0001	n.s.	n.s.	

Supplementary Table S4 Statistical analyses (p-values) concerning the expression of *Vimentin*/*VIM* in cells without experimental EMT induction (n.s. = not significant)

VIM	GK	GK EtOH Ko	GK EtOH	EPI	EPI EtOH Ko	EPI EtOH	FIB	FIB EtOH Ko	FIB EtOH
GK		n.s.	n.s.	n.s.	<0,0001	n.s.	<0,0001	<0,0001	<0,0001
GK EtOH Ko	n.s.		n.s.	n.s.	0.0048	n.s.	<0,0001	<0,0001	<0,0001
GK EtOH	n.s.	n.s.		n.s.	0.0196	n.s.	<0,0001	<0,0001	<0,0001
EPI	n.s.	n.s.	n.s.		0.0149	n.s.	<0,0001	<0,0001	<0,0001
EPI EtOH Ko	<0,0001	0.0048	0.0196	0.0149		<0,0001	0.0116	0.012	<0,0001
EPI EtOH	n.s.	n.s.	n.s.	n.s.	<0,0001		<0,0001	<0,0001	<0,0001
FIB	<0,0001	<0,0001	<0,0001	<0,0001	0.0116	<0,0001		n.s.	n.s.
FIB EtOH Ko	<0,0001	<0,0001	<0,0001	<0,0001	0.012	<0,0001	n.s.		n.s.
FIB EtOH	<0,0001	<0,0001	<0,0001	<0,0001	<0,0001	<0,0001	n.s.	n.s.	

Supplementary Table S5 Statistical analyses (p-values) concerning the expression of *Integrin β-1*/*ITGB1* in cells without experimental EMT induction (n.s. = not significant)

ITGB1	GK	GK EtOH Ko	GK EtOH	EPI	EPI EtOH Ko	EPI EtOH	FIB	FIB EtOH Ko	FIB EtOH
GK		n.s.	n.s.	n.s.	n.s.	n.s.	n.s.	0.0226	0.0003
GK EtOH Ko	n.s.		n.s.	n.s.	n.s.	n.s.	n.s.	n.s.	n.s.
GK EtOH	n.s.	n.s.		n.s.	n.s.	0.0355	n.s.	n.s.	n.s.
EPI	n.s.	n.s.	n.s.		n.s.	n.s.	n.s.	n.s.	0.0026
EPI EtOH Ko	n.s.	n.s.	n.s.	n.s.		n.s.	n.s.	n.s.	0.01
EPI EtOH	n.s.	n.s.	0.0355	n.s.	n.s.		0.049	0.014	0.0002
FIB	n.s.	n.s.	n.s.	n.s.	n.s.	0.049		n.s.	n.s.
FIB EtOH Ko	0.0226	n.s.	n.s.	n.s.	n.s.	0.014	n.s.		n.s.
FIB EtOH	0.0003	n.s.	n.s.	0.0026	0.01	0.0002	n.s.	n.s.	

Supplementary Table S6 Statistical analyses (p-values) concerning the expression of *FAK*/*PTK2* in cells without experimental EMT induction (n.s. = not significant)

PTK2	GK	GK EtOH Ko	GK EtOH	EPI	EPI EtOH Ko	EPI EtOH	FIB	FIB EtOH Ko	FIB EtOH
GK		n.s.	0.0377	0.0205	0.0077	n.s.	n.s.	n.s.	n.s.
GK EtOH Ko	n.s.		n.s.	n.s.	n.s.	n.s.	n.s.	0.0261	n.s.
GK EtOH	0.0377	n.s.		n.s.	n.s.	n.s.	0.0206	0.009	n.s.
EPI	0.0205	n.s.	n.s.		n.s.	n.s.	0.0111	0.0048	n.s.
EPI EtOH Ko	0.0077	n.s.	n.s.	n.s.		n.s.	0.0041	0.0018	n.s.
EPI EtOH	n.s.	n.s.	n.s.	n.s.	n.s.		n.s.	n.s.	n.s.
FIB	n.s.	n.s.	0.0206	0.0111	0.0041	n.s.		n.s.	n.s.
FIB EtOH Ko	n.s.	0.0261	0.009	0.0048	0.0018	n.s.	n.s.		n.s.
FIB EtOH	n.s.	n.s.	n.s.	n.s.	n.s.	n.s.	n.s.	n.s.	

Supplementary Table S7 Statistical analyses (p-values) concerning the expression of *Merlin/NF2* in cells without experimental EMT induction (n.s. = not significant)

NF2	GK	GK EtOH Ko	GK EtOH	EPI	EPI EtOH Ko	EPI EtOH	FIB	FIB EtOH Ko	FIB EtOH
GK		n.s.	0.0142	n.s.	n.s.	n.s.	n.s.	n.s.	n.s.
GK EtOH Ko	n.s.		n.s.	n.s.	n.s.	0.0049	n.s.	n.s.	n.s.
GK EtOH	0.0142	n.s.		0.0482	0.0264	0.0004	0.006	0.0231	n.s.
EPI	n.s.	n.s.	0.0482		n.s.	n.s.	n.s.	n.s.	n.s.
EPI EtOH Ko	n.s.	n.s.	0.0264	n.s.		n.s.	n.s.	n.s.	n.s.
EPI EtOH	n.s.	0.0049	0.0004	n.s.	n.s.		n.s.	n.s.	0.006
FIB	n.s.	n.s.	0.006	n.s.	n.s.	n.s.		n.s.	n.s.
FIB EtOH Ko	n.s.	n.s.	0.0231	n.s.	n.s.	n.s.	n.s.		n.s.
FIB EtOH	n.s.	n.s.	n.s.	n.s.	n.s.	0.006	n.s.	n.s.	

Supplementary Table S8 Statistical analyses (p-values) concerning the expression of *YAP1* in cells without experimental EMT induction (n.s. = not significant)

YAP1	GK	GK EtOH Ko	GK EtOH	EPI	EPI EtOH Ko	EPI EtOH	FIB	FIB EtOH Ko	FIB EtOH
GK		n.s.	0.0236	n.s.	n.s.	n.s.	0.0049	0.0125	n.s.
GK EtOH Ko	n.s.		n.s.	0.0413	n.s.	0.0009	<0,0001	<0,0001	0.0092
GK EtOH	0.0236	n.s.		0.0037	n.s.	<0,0001	<0,0001	<0,0001	0.0008
EPI	n.s.	0.0413	0.0037		n.s.	n.s.	0.0311	n.s.	n.s.
EPI EtOH Ko	n.s.	n.s.	n.s.	n.s.		0.0045	<0,0001	0.0002	0.0432
EPI EtOH	n.s.	0.0009	<0,0001	n.s.	0.0045		n.s.	n.s.	n.s.
FIB	0.0049	<0,0001	<0,0001	0.0311	<0,0001	n.s.		n.s.	n.s.
FIB EtOH Ko	0.0125	<0,0001	<0,0001	n.s.	0.0002	n.s.	n.s.		n.s.
FIB EtOH	n.s.	0.0092	0.0008	n.s.	0.0432	n.s.	n.s.	n.s.	

Supplementary Table S9 Statistical analyses (p-values) concerning the expression of *TAZ* in cells without experimental EMT induction (n.s. = not significant)

TAZ	GK	GK EtOH Ko	GK EtOH	EPI	EPI EtOH Ko	EPI EtOH	FIB	FIB EtOH Ko	FIB EtOH
GK		n.s.	0.0234	n.s.	n.s.	n.s.	0.0093	0.0046	n.s.
GK EtOH Ko	n.s.		n.s.	0.0196	n.s.	0.0021	<0,0001	<0,0001	0.0459
GK EtOH	0.0234	n.s.		0.0024	0.0265	0.0003	<0,0001	<0,0001	0.0057
EPI	n.s.	0.0196	0.0024		n.s.	n.s.	n.s.	0.044	n.s.
EPI EtOH Ko	n.s.	n.s.	0.0265	n.s.		n.s.	0.0082	0.004	n.s.
EPI EtOH	n.s.	0.0021	0.0003	n.s.	n.s.		n.s.	n.s.	n.s.
FIB	0.0093	<0,0001	<0,0001	n.s.	0.0082	n.s.		n.s.	0.0374
FIB EtOH Ko	0.0046	<0,0001	<0,0001	0.044	0.004	n.s.	n.s.		0.0187
FIB EtOH	n.s.	0.0459	0.0057	n.s.	n.s.	n.s.	0.0374	0.0187	

Supplementary Table S10 Statistical analyses (p-values) concerning the expression of *TEAD1* in cells without experimental EMT induction (n.s. = not significant)

TEAD1	GK	GK EtOH Ko	GK EtOH	EPI	EPI EtOH Ko	EPI EtOH	FIB	FIB EtOH Ko	FIB EtOH
GK		n.s.	0.0137	n.s.	n.s.	n.s.	n.s.	n.s.	n.s.
GK EtOH Ko	n.s.		n.s.	0.0055	0.0159	0.0004	0.0103	0.0111	n.s.
GK EtOH	0.0137	n.s.		0.0007	0.0022	<0,0001	0.0014	0.0015	n.s.
EPI	n.s.	0.0055	0.0007		n.s.	n.s.	n.s.	n.s.	n.s.
EPI EtOH Ko	n.s.	0.0159	0.0022	n.s.		n.s.	n.s.	n.s.	n.s.
EPI EtOH	n.s.	0.0004	<0,0001	n.s.	n.s.		n.s.	n.s.	0.0088
FIB	n.s.	0.0103	0.0014	n.s.	n.s.	n.s.		n.s.	n.s.
FIB EtOH Ko	n.s.	0.0111	0.0015	n.s.	n.s.	n.s.	n.s.		n.s.
FIB EtOH	n.s.	n.s.	n.s.	n.s.	n.s.	0.0088	n.s.	n.s.	

Supplementary Table S11 Statistical analyses (p-values) concerning the expression of *Oct4/POU5F1* in cells without experimental EMT induction (n.s. = not significant)

**POU5F1**	**GK**	**GK EtOH Ko**	**GK EtOH**	**EPI**	**EPI EtOH Ko**	**EPI EtOH**	**FIB**	**FIB EtOH Ko**	**FIB EtOH**
**GK**		n.s.	n.s.	n.s.	n.s.	n.s.	n.s.	n.s.	0.0251
**GK EtOH Ko**	n.s.		n.s.	n.s.	n.s.	n.s.	n.s.	n.s.	n.s.
**GK EtOH**	n.s.	n.s.		n.s.	n.s.	n.s.	n.s.	0.0365	n.s.
**EPI**	n.s.	n.s.	n.s.		n.s.	n.s.	n.s.	n.s.	n.s.
**EPI EtOH Ko**	n.s.	n.s.	n.s.	n.s.		n.s.	n.s.	n.s.	0.0265
**EPI EtOH**	n.s.	n.s.	n.s.	n.s.	n.s.		n.s.	n.s.	n.s.
**FIB**	n.s.	n.s.	n.s.	n.s.	n.s.	n.s.		n.s.	n.s.
**FIB EtOH Ko**	n.s.	n.s.	0.0365	n.s.	n.s.	n.s.	n.s.		0.0049
**FIB EtOH**	0.0251	n.s.	n.s.	n.s.	0.0265	n.s.	n.s.	0.0049	

Supplementary Table S12 Statistical analyses (p-values) concerning the expression of *ZEB1* in cells without experimental EMT induction (n.s. = not significant)

**ZEB1**	**GK**	**GK EtOH Ko**	**GK EtOH**	**EPI**	**EPI EtOH Ko**	**EPI EtOH**	**FIB**	**FIB EtOH Ko**	**FIB EtOH**
**GK**		n.s.	0.0011	n.s.	n.s.	0.0165	<0,0001	<0,0001	<0,0001
**GK EtOH Ko**	n.s.		n.s.	0.0004	n.s.	<0,0001	0.0064	0.0059	<0,0001
**GK EtOH**	0.0011	n.s.		<0,0001	0.0013	<0,0001	n.s.	n.s.	0.0001
**EPI**	n.s.	0.0004	<0,0001		n.s.	n.s.	<0,0001	<0,0001	<0,0001
**EPI EtOH Ko**	n.s.	n.s.	0.0013	n.s.		0.0141	<0,0001	<0,0001	<0,0001
**EPI EtOH**	0.0165	<0,0001	<0,0001	n.s.	0.0141		<0,0001	<0,0001	<0,0001
**FIB**	<0,0001	0.0064	n.s.	<0,0001	<0,0001	<0,0001		n.s.	0.0244
**FIB EtOH Ko**	<0,0001	0.0059	n.s.	<0,0001	<0,0001	<0,0001	n.s.		0.0263
**FIB EtOH**	<0,0001	<0,0001	0.0001	<0,0001	<0,0001	<0,0001	0.0244	0.0263	

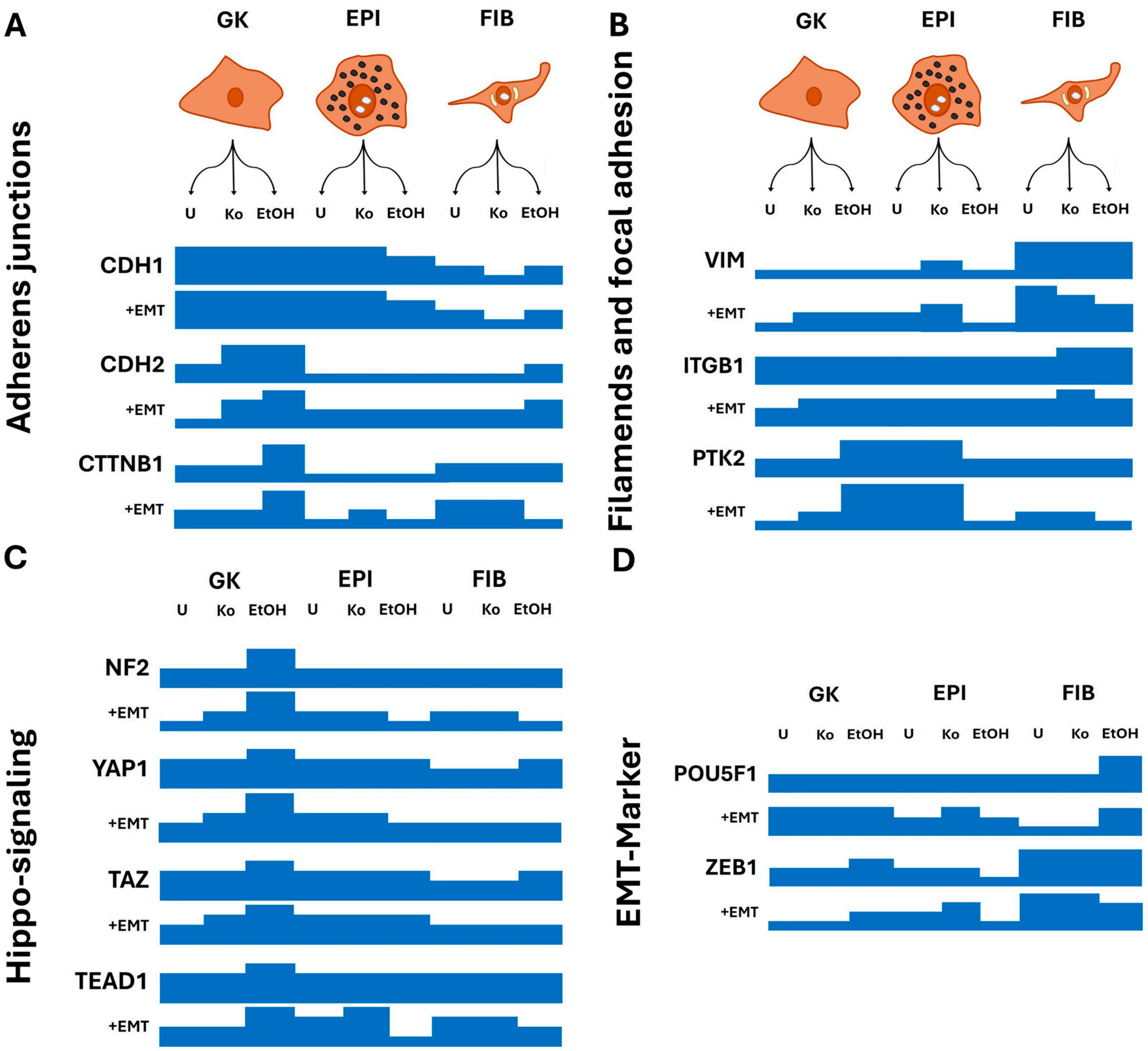

Supplementary Figure S3 Schematic summary of the effects of cell culture conditions, EtOH-treatment and "+EMT"-treatment on gene expression in GK, EPI and FIB cells and their derivatives as detected by quantitative PCR. U = untreated cell line; EtOH = EtOH-treatment; Ko = control culture condition; +EMT = treated with EMT-inducing medium. For color/symbol legend see Figure 1. The upper blue bars indicate the relative expression of the respective genes in comparison with GK cells (higher bars indicate a higher gene expression). The lower blue bars represent the direct comparison of gene expression in the respective cell line with and without addition of the EMT cocktail. A: Analysis of marker genes belonging to adherens junctions (AJs): *CDH1*, *CDH2* and *CTNNB1.* B: Analysis of marker genes belonging to focal adhesions (FAs) and the filament protein family: *VIM*, *ITGB1* and *PTK2*. C: Analysis of marker genes belonging to the Hippo-signaling pathway: *NF2*, *YAP1*, *TAZ* and *TEAD1*. D: Analysis of EMT-marker genes: *POU5F1* and *ZEB1*. Created in BioRender. Steinberg, T. (2025) https://BioRender.com/4gpars1

Supplementary Table S13 Statistical analyses (p-values) concerning the expression of different genes in untreated ("-EMT") and treated ("+EMT") cells

p-Wert	GK -EMT vs. GK +EMT	GK EtOH Ko -EMT vs. GK EtOH Ko +EMT	GK EtOH -EMT vs. GK EtOH +EMT	EPI -EMT vs. EPI +EMT	EPI EtOH Ko -EMT vs. EPI EtOH Ko +EMT	EPI EtOH -EMT vs. EPI EtOH +EMT	FIB -EMT vs. FIB +EMT	FIB EtOH Ko -EMT vs. FIB EtOH Ko +EMT	FIB EtOH -EMT vs. FIB EtOH +EMT
CDH1	0.5733	0.7186	0.4617	0.6335	0.2036	0.0953	0.6812	0.5962	0.8045
CDH2	0.199	0.5668	0.9961	0.0081	0.0041	0.2433	0.0001	0.0027	0.3756
CTNNB1	0.7056	0.1625	0.9628	0.2968	0.193	0.8914	0.0181	0.0311	0.0844
VIM	0.6366	0.5782	0.2482	0.0025	0.0248	0.7221	0.0005	0.5006	0.1884
ITGB1	0.3841	0.7092	0.7563	0.2396	0.0696	0.7896	0.3907	0.234	0.1126
PTK2	0.4747	0.8073	0.5038	0.5529	0.0398	0.3621	0.3952	0.7325	0.1735
NF2	0.2724	0.65	0.6676	0.7887	0.2181	0.0773	0.6628	0.6331	0.091
YAP1	0.5931	0.8832	0.4842	0.9037	0.3101	0.1868	0.8557	0.674	0.0544
TAZ	0.3173	0.4932	0.9371	0.8596	0.2506	0.7027	0.3345	0.801	0.1159
TEAD1	0.3645	0.636	0.9949	0.8097	0.1292	0.1875	0.6955	0.7398	0.141
POU5F1	0.6018	0.3846	0.1458	0.7808	0.2495	0.6076	0.0316	0.277	0.0363
ZEB1	0.0329	0.057	0.1314	0.3365	0.044	0.6342	0.1	0.9083	0.1201

Supplementary Table S14 Statistical analyses (p-values) concerning the amount of E-Cadherin protein in cells without experimental EMT induction (n.s. = not significant)

E-Cadherin	GK	GK EtOH Ko	GK EtOH	EPI	EPI EtOH Ko	EPI EtOH	FIB	FIB EtOH Ko	FIB EtOH
GK		n.s.	n.s.	n.s.	n.s.	0.0013	0.0045	0.0013	0.0013
GK EtOH Ko	n.s.		n.s.	n.s.	n.s.	0.0054	0.0187	0.0054	0.0054
GK EtOH	n.s.	n.s.		n.s.	n.s.	n.s.	n.s.	n.s.	n.s.
EPI	n.s.	n.s.	n.s.		n.s.	0.0003	0.0011	0.0003	0.0003
EPI EtOH Ko	n.s.	n.s.	n.s.	n.s.		n.s.	n.s.	n.s.	n.s.
EPI EtOH	0.0013	0.0054	n.s.	0.0003	n.s.		n.s.	n.s.	n.s.
FIB	0.0045	0.0187	n.s.	0.0011	n.s.	n.s.		n.s.	n.s.
FIB EtOH Ko	0.0013	0.0054	n.s.	0.0003	n.s.	n.s.	n.s.		n.s.
FIB EtOH	0.0013	0.0054	n.s.	0.0003	n.s.	n.s.	n.s.	n.s.	

Supplementary Table S15 Statistical analyses (p-values) concerning the amount of N-Cadherin protein in cells without experimental EMT induction (n.s. = not significant)

N-Cadherin	GK	GK EtOH Ko	GK EtOH	EPI	EPI EtOH Ko	EPI EtOH	FIB	FIB EtOH Ko	FIB EtOH
GK		n.s.	n.s.	n.s.	n.s.	<0,0001	<0,0001	<0,0001	<0,0001
GK EtOH Ko	n.s.		n.s.	n.s.	n.s.	0.0028	0.0028	0.0028	0.0028
GK EtOH	n.s.	n.s.		n.s.	n.s.	0.0034	0.0034	0.0034	0.0034
EPI	n.s.	n.s.	n.s.		0.0057	<0,0001	<0,0001	<0,0001	<0,0001
EPI EtOH Ko	n.s.	n.s.	n.s.	0.0057		0.0353	0.0353	0.0353	0.0353
EPI EtOH	<0,0001	0.0028	0.0034	<0,0001	0.0353		n.s.	n.s.	n.s.
FIB	<0,0001	0.0028	0.0034	<0,0001	0.0353	n.s.		n.s.	n.s.
FIB EtOH Ko	<0,0001	0.0028	0.0034	<0,0001	0.0353	n.s.	n.s.		n.s.
FIB EtOH	<0,0001	0.0028	0.0034	<0,0001	0.0353	n.s.	n.s.	n.s.	

Supplementary Table S16 Statistical analyses (p-values) concerning the amount of β-Catenin protein in cells without experimental EMT induction (n.s. = not significant)

β-Catenin	GK	GK EtOH Ko	GK EtOH	EPI	EPI EtOH Ko	EPI EtOH	FIB	FIB EtOH Ko	FIB EtOH
GK		0.001	n.s.	0.0004	0.0004	0.0004	0.0045	0.0004	0.0004
GK EtOH Ko	0.001		n.s.	<0,0001	<0,0001	<0,0001	<0,0001	<0,0001	<0,0001
GK EtOH	n.s.	n.s.		<0,0001	<0,0001	<0,0001	<0,0001	<0,0001	<0,0001
EPI	0.0004	<0,0001	<0,0001		n.s.	n.s.	n.s.	n.s.	n.s.
EPI EtOH Ko	0.0004	<0,0001	<0,0001	n.s.		n.s.	n.s.	n.s.	n.s.
EPI EtOH	0.0004	<0,0001	<0,0001	n.s.	n.s.		n.s.	n.s.	n.s.
FIB	0.0045	<0,0001	<0,0001	n.s.	n.s.	n.s.		n.s.	n.s.
FIB EtOH Ko	0.0004	<0,0001	<0,0001	n.s.	n.s.	n.s.	n.s.		n.s.
FIB EtOH	0.0004	<0,0001	<0,0001	n.s.	n.s.	n.s.	n.s.	n.s.	

Supplementary Table S17 Statistical analyses (p-values) concerning the amount of Vimentin protein in cells without experimental EMT induction (n.s. = not significant)

Vimentin	GK	GK EtOH Ko	GK EtOH	EPI	EPI EtOH Ko	EPI EtOH	FIB	FIB EtOH Ko	FIB EtOH
GK		n.s.	n.s.	n.s.	n.s.	n.s.	<0,0001	<0,0001	<0,0001
GK EtOH Ko	n.s.		n.s.	n.s.	n.s.	n.s.	<0,0001	<0,0001	<0,0001
GK EtOH	n.s.	n.s.		n.s.	n.s.	n.s.	<0,0001	<0,0001	<0,0001
EPI	n.s.	n.s.	n.s.		n.s.	n.s.	<0,0001	<0,0001	<0,0001
EPI EtOH Ko	n.s.	n.s.	n.s.	n.s.		n.s.	<0,0001	<0,0001	<0,0001
EPI EtOH	n.s.	n.s.	n.s.	n.s.	n.s.		<0,0001	<0,0001	<0,0001
FIB	<0,0001	<0,0001	<0,0001	<0,0001	<0,0001	<0,0001		<0,0001	<0,0001
FIB EtOH Ko	<0,0001	<0,0001	<0,0001	<0,0001	<0,0001	<0,0001	<0,0001		n.s.
FIB EtOH	<0,0001	<0,0001	<0,0001	<0,0001	<0,0001	<0,0001	<0,0001	n.s.	

Supplementary Table S18 Statistical analyses (p-values) concerning the amount of Merlin protein in cells without experimental EMT induction (n.s. = not significant)

Merlin	GK	GK EtOH Ko	GK EtOH	EPI	EPI EtOH Ko	EPI EtOH	FIB	FIB EtOH Ko	FIB EtOH
GK		n.s.	n.s.	n.s.	n.s.	n.s.	n.s.	0.0002	0.0001
GK EtOH Ko	n.s.		n.s.	n.s.	n.s.	n.s.	n.s.	0.0005	0.0003
GK EtOH	n.s.	n.s.		n.s.	n.s.	n.s.	n.s.	0.0004	0.0002
EPI	n.s.	n.s.	n.s.		n.s.	n.s.	n.s.	0.0002	0.0001
EPI EtOH Ko	n.s.	n.s.	n.s.	n.s.		n.s.	n.s.	0.0003	0.0002
EPI EtOH	n.s.	n.s.	n.s.	n.s.	n.s.		n.s.	0.0002	0.0001
FIB	n.s.	n.s.	n.s.	n.s.	n.s.	n.s.		0.0034	0.002
FIB EtOH Ko	0.0002	0.0005	0.0004	0.0002	0.0003	0.0002	0.0034		n.s.
FIB EtOH	0.0001	0.0003	0.0002	0.0001	0.0002	0.0001	0.002	n.s.	

Supplementary Table S19 Statistical analyses (p-values) concerning the amount of YAP1 protein in cells without experimental EMT induction (n.s. = not significant)

YAP1	GK	GK EtOH Ko	GK EtOH	EPI	EPI EtOH Ko	EPI EtOH	FIB	FIB EtOH Ko	FIB EtOH
GK		n.s.	n.s.	n.s.	n.s.	n.s.	n.s.	n.s.	n.s.
GK EtOH Ko	n.s.		n.s.	n.s.	n.s.	n.s.	n.s.	n.s.	n.s.
GK EtOH	n.s.	n.s.		n.s.	n.s.	n.s.	n.s.	n.s.	n.s.
EPI	n.s.	n.s.	n.s.		n.s.	n.s.	n.s.	n.s.	n.s.
EPI EtOH Ko	n.s.	n.s.	n.s.	n.s.		n.s.	n.s.	n.s.	n.s.
EPI EtOH	n.s.	n.s.	n.s.	n.s.	n.s.		n.s.	n.s.	n.s.
FIB	n.s.	n.s.	n.s.	n.s.	n.s.	n.s.		n.s.	n.s.
FIB EtOH Ko	n.s.	n.s.	n.s.	n.s.	n.s.	n.s.	n.s.		n.s.
FIB EtOH	n.s.	n.s.	n.s.	n.s.	n.s.	n.s.	n.s.	n.s.	

Supplementary Table S20 Statistical analyses (p-values) concerning the amount of TAZ protein in cells without experimental EMT induction (n.s. = not significant)

TAZ	GK	GK EtOH Ko	GK EtOH	EPI	EPI EtOH Ko	EPI EtOH	FIB	FIB EtOH Ko	FIB EtOH
GK		n.s.	n.s.	n.s.	n.s.	n.s.	n.s.	n.s.	n.s.
GK EtOH Ko	n.s.		n.s.	n.s.	n.s.	n.s.	n.s.	n.s.	n.s.
GK EtOH	n.s.	n.s.		n.s.	n.s.	n.s.	n.s.	n.s.	n.s.
EPI	n.s.	n.s.	n.s.		n.s.	n.s.	n.s.	n.s.	n.s.
EPI EtOH Ko	n.s.	n.s.	n.s.	n.s.		n.s.	n.s.	n.s.	n.s.
EPI EtOH	n.s.	n.s.	n.s.	n.s.	n.s.		n.s.	n.s.	n.s.
FIB	n.s.	n.s.	n.s.	n.s.	n.s.	n.s.		n.s.	n.s.
FIB EtOH Ko	n.s.	n.s.	n.s.	n.s.	n.s.	n.s.	n.s.		n.s.
FIB EtOH	n.s.	n.s.	n.s.	n.s.	n.s.	n.s.	n.s.	n.s.	

Supplementary Table 21 Statistical analyses (p-values) concerning the amount of TEAD2 protein in cells without experimental EMT induction (n.s. = not significant)

TEAD2	GK	GK EtOH Ko	GK EtOH	EPI	EPI EtOH Ko	EPI EtOH	FIB	FIB EtOH Ko	FIB EtOH
GK		n.s.	n.s.	n.s.	n.s.	n.s.	n.s.	n.s.	n.s.
GK EtOH Ko	n.s.		n.s.	0.0027	n.s.	0.0018	0.0237	0.005	0.0022
GK EtOH	n.s.	n.s.		n.s.	n.s.	n.s.	n.s.	n.s.	n.s.
EPI	n.s.	0.0027	n.s.		n.s.	n.s.	n.s.	n.s.	n.s.
EPI EtOH Ko	n.s.	n.s.	n.s.	n.s.		n.s.	n.s.	n.s.	n.s.
EPI EtOH	n.s.	0.0018	n.s.	n.s.	n.s.		n.s.	n.s.	n.s.
FIB	n.s.	0.0237	n.s.	n.s.	n.s.	n.s.		n.s.	n.s.
FIB EtOH Ko	n.s.	0.005	n.s.	n.s.	n.s.	n.s.	n.s.		n.s.
FIB EtOH	n.s.	0.0022	n.s.	n.s.	n.s.	n.s.	n.s.	n.s.	

Supplementary Table S22 Statistical analyses (p-values) concerning the amount of Oct4 protein in cells without experimental EMT induction (n.s. = not significant)

Oct4	GK	GK EtOH Ko	GK EtOH	EPI	EPI EtOH Ko	EPI EtOH	FIB	FIB EtOH Ko	FIB EtOH
GK		n.s.	n.s.	n.s.	n.s.	n.s.	n.s.	n.s.	n.s.
GK EtOH Ko	n.s.		n.s.	n.s.	n.s.	n.s.	n.s.	n.s.	n.s.
GK EtOH	n.s.	n.s.		n.s.	n.s.	n.s.	n.s.	n.s.	n.s.
EPI	n.s.	n.s.	n.s.		n.s.	n.s.	n.s.	n.s.	n.s.
EPI EtOH Ko	n.s.	n.s.	n.s.	n.s.		n.s.	n.s.	n.s.	n.s.
EPI EtOH	n.s.	n.s.	n.s.	n.s.	n.s.		n.s.	n.s.	n.s.
FIB	n.s.	n.s.	n.s.	n.s.	n.s.	n.s.		n.s.	n.s.
FIB EtOH Ko	n.s.	n.s.	n.s.	n.s.	n.s.	n.s.	n.s.		n.s.
FIB EtOH	n.s.	n.s.	n.s.	n.s.	n.s.	n.s.	n.s.	n.s.	

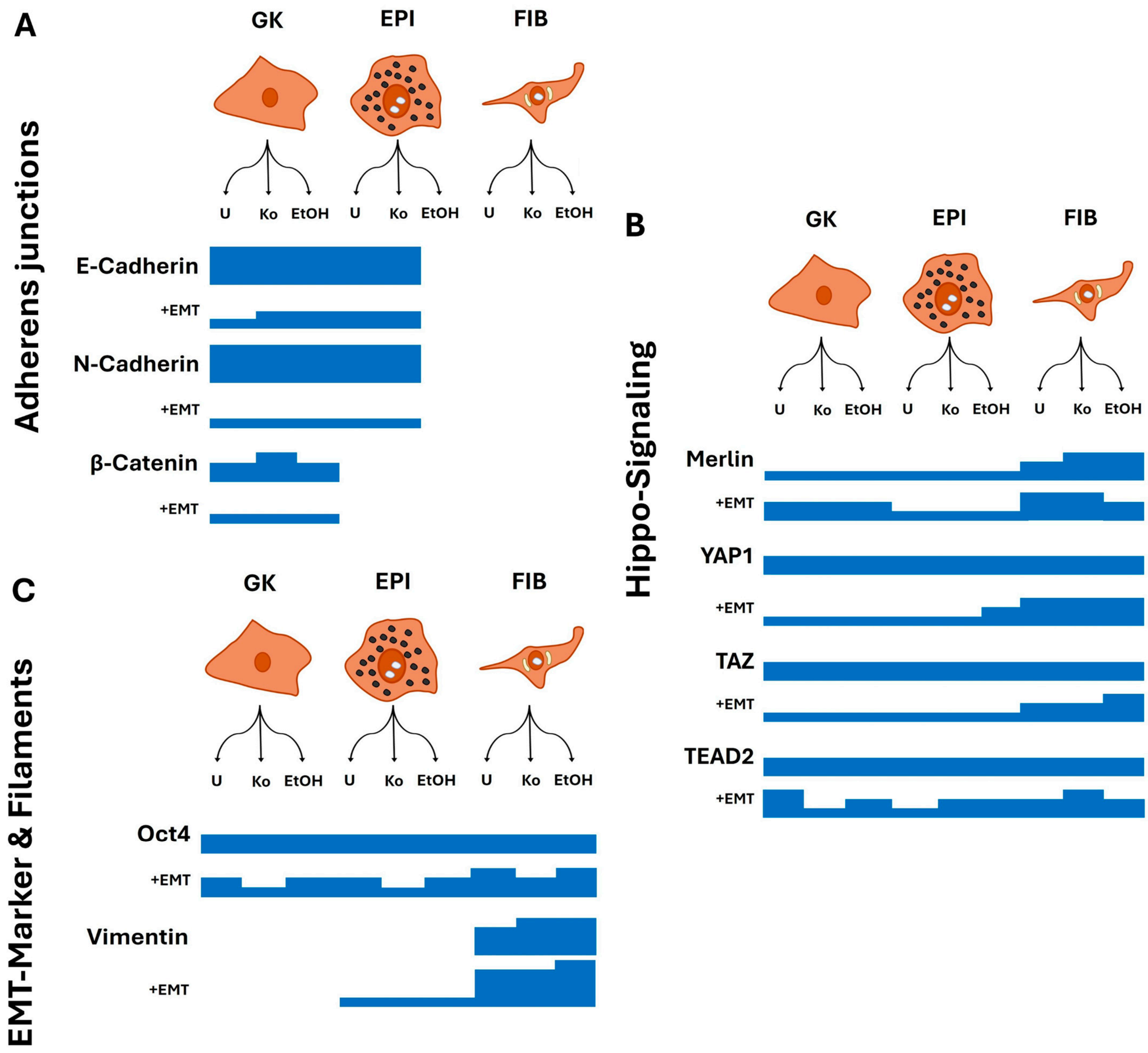

Supplementary Figure S4 Schematic summary of the effects of cell culture conditions, EtOH-treatment and "+EMT"-treatment on protein amounts in GK, EPI and FIB cells and their derivatives as detected by Western Blot. U = untreated cell line; EtOH = EtOH-treatment; Ko = control culture condition; +EMT = treated with EMT-inducing medium. For color/symbol legend see Figure 1. The upper blue bars indicate the relative amounts of the respective proteins in comparison with GK cells (higher bars indicate a higher protein amount). The lower blue bars represent the direct comparison of protein amounts in the respective cell line with and without addition of the EMT cocktail. A: Analysis of marker proteins belonging to adherens junctions (AJs): E-Cadherin, N-Cadherin and β-Catenin. B: Analysis of marker proteins belonging to the Hippo-signaling pathway: Merlin, YAP1, TAZ and TEAD2. C: Analysis of EMT-markers and filaments: Oct4 and Vimentin. Created in BioRender. Steinberg, T. (2025) https://BioRender.com/ulwh3c3

Supplementary Table S23 Statistical analyses (p-values) concerning the amounts of different proteins in untreated ("-EMT") and treated ("+EMT") cells

p-value	GK -EMT vs. GK +EMT	GK EtOH Ko -EMT vs. GK EtOH Ko +EMT	GK EtOH -EMT vs. GK EtOH +EMT	EPI -EMT vs. EPI +EMT	EPI EtOH Ko -EMT vs. EPI EtOH Ko +EMT	EPI EtOH -EMT vs. EPI EtOH +EMT	FIB -EMT vs. FIB +EMT	FIB EtOH Ko -EMT vs. FIB EtOH Ko +EMT	FIB EtOH -EMT vs. FIB EtOH +EMT
E-Cadherin	0.0307	0.3559	0.3545	0.0633	0.3277	0.6119	0.8387	0.8542	0.6055
N-Cadherin	0.0922	0.01	0.0286	0.0063	0.0994	-	-	-	-
β-Catenin	0.009	0.0085	0.0018	0.9063	0.5515	0.7897	0.909	0.3739	-
Vimentin	-	0.9031	0.8404	0.1265	0.3749	0.221	0.5582	0.9937	0.6007
Merlin	0.2019	0.5144	0.8589	0.7113	0.9562	0.9879	0.2096	0.9619	0.6345
YAP1	0.4643	0.7936	0.7102	0.3701	0.7494	0.9725	0.0373	0.6788	0.7878
TAZ	0.6242	0.7436	0.8329	0.5838	0.5269	0.7595	0.7742	0.5492	0.4704
TEAD2	0.5573	0.3146	0.9335	0.055	0.8074	0.8236	0.925	0.2005	0.896
Oct4	0.8735	0.7978	0.9095	0.8945	0.6601	0.8126	0.5107	0.885	0.6776

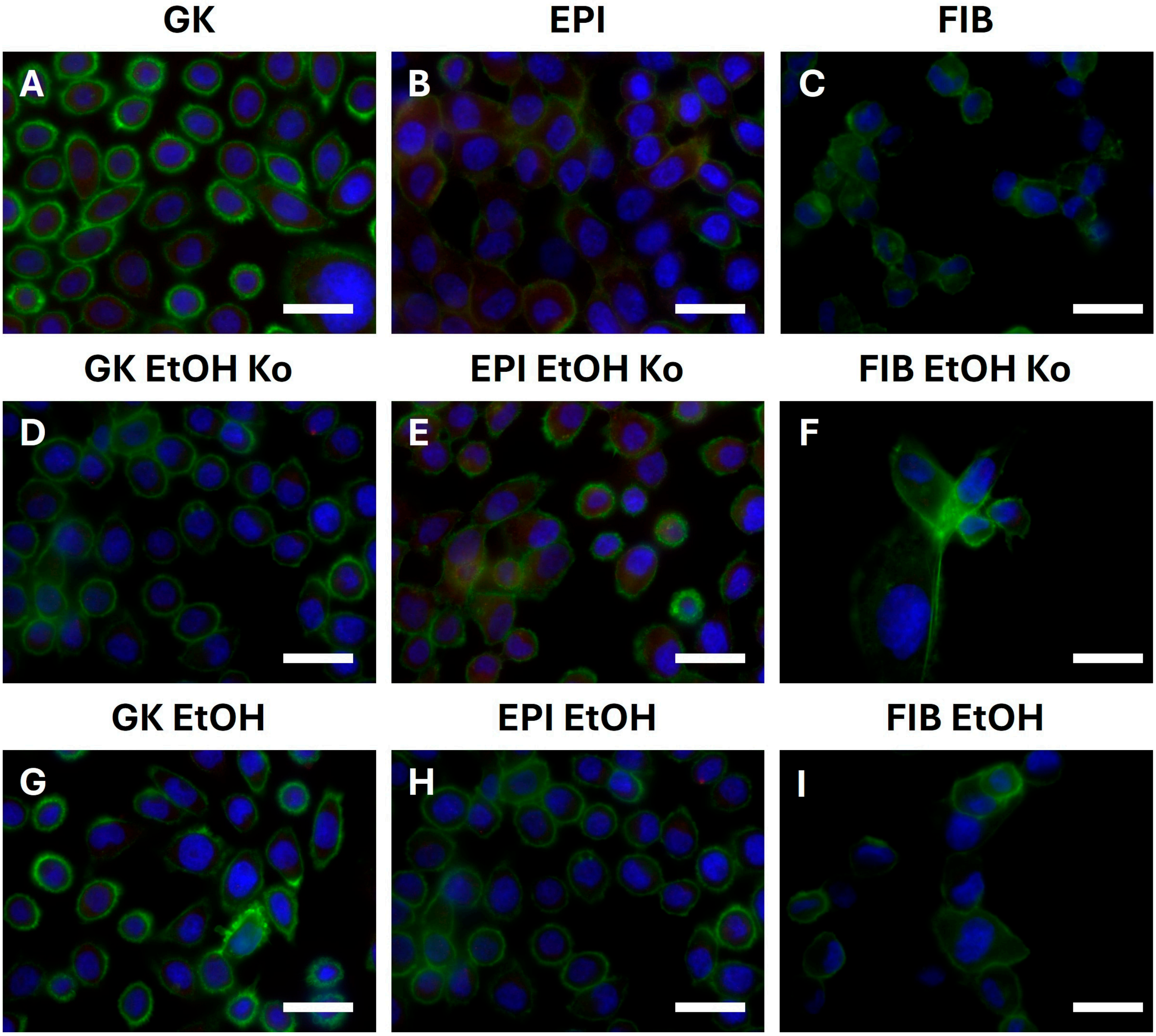

Supplementary Figure S5 Indirect immunofluorescence (IIF) micrographs with specific detection of E-Cadherin in GK, EPI and FIB cells as well as their derivatives. The specific protein signal is depicted in red, the cellular actin cytoskeleton in green and cell nuclei in blue. A: GK cells; B: EPI cells; C: FIB cells; D: GK EtOH Ko cells, E: EPI EtOH Ko cells; F: FIB EtOH Ko cells; G: GK EtOH cells; H: EPI EtOH cells; I: FIB EtOH cells. Scale bars = 40 µm.

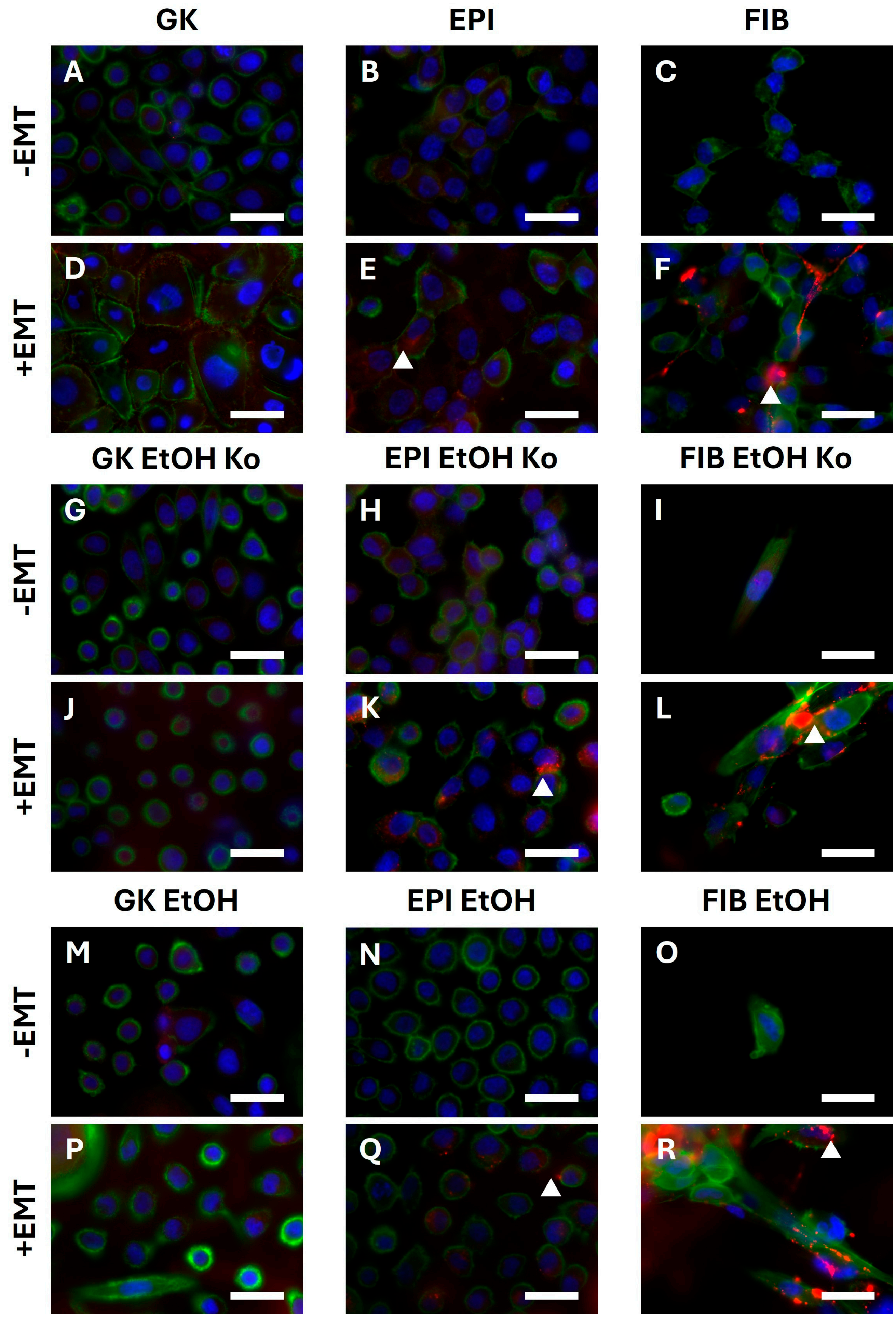
GK
EPI
FIB
-EMT
+EMT
A
B
C
D
E
F
GK EtOH Ko
EPI EtOH Ko
FIB EtOH Ko
G
H
I
J
K
L
GK EtOH
EPI EtOH
FIB EtOH
M
N
O
P
Q
R

Supplementary Figure S6 Indirect immunofluorescence (IIF) micrographs with specific detection of E-Cadherin in GK, EPI and FIB cells as well as their derivatives without ("-EMT") and with ("+EMT") EMT-inducing cocktail. The specific protein signal is depicted in red (see white arrowheads), the cellular actin cytoskeleton in green and cell nuclei in blue. A and D: GK cells; B and E: EPI cells; C and F: FIB cells; G and J: GK EtOH Ko cells, H and K: EPI EtOH Ko cells; I and L: FIB EtOH Ko cells; M and P: GK EtOH cells; N and Q: EPI EtOH cells; O and R: FIB EtOH cells. Scale bars = 40 μm.

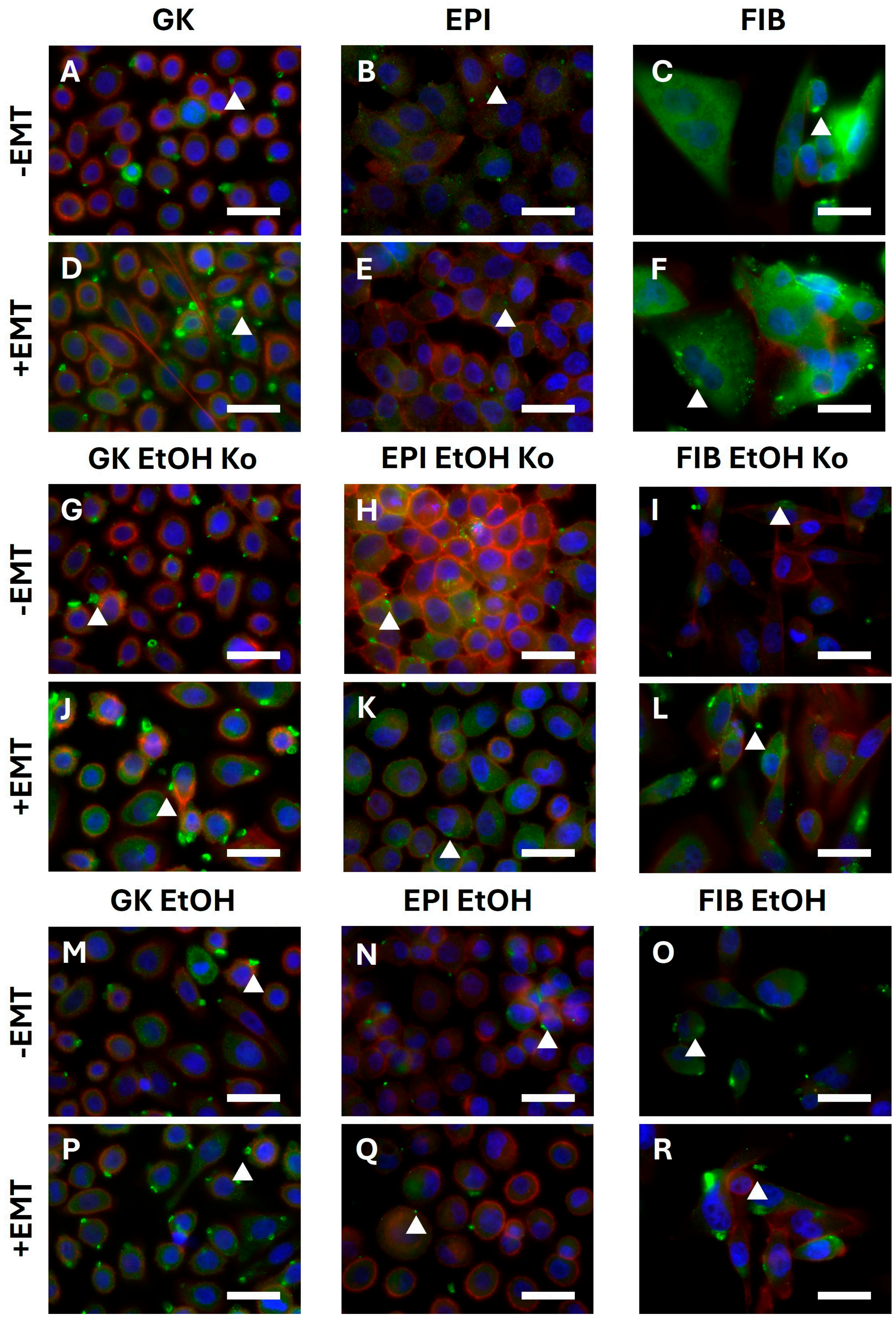
GK
EPI
FIB
-EMT
+EMT
A
B
C
D
E
F
GK EtOH Ko
EPI EtOH Ko
FIB EtOH Ko
G
H
I
J
K
L
GK EtOH
EPI EtOH
FIB EtOH
M
N
O
P
Q
R

Supplementary Figure S7 Indirect immunofluorescence (IIF) micrographs with specific detection of N-Cadherin in GK, EPI and FIB cells as well as their derivatives without ("-EMT") and with ("+EMT") EMT-inducing cocktail. The specific protein signal is depicted in green (see white arrowhead), the cellular actin cytoskeleton in red and cell nuclei in blue. A and D: GK cells; B and E: EPI cells; C and F: FIB cells; G and J: GK EtOH Ko cells, H and K: EPI EtOH Ko cells; I and L: FIB EtOH Ko cells; M and P: GK EtOH cells; N and Q: EPI EtOH cells; O and R: FIB EtOH cells. Scale bars = 40 µm.

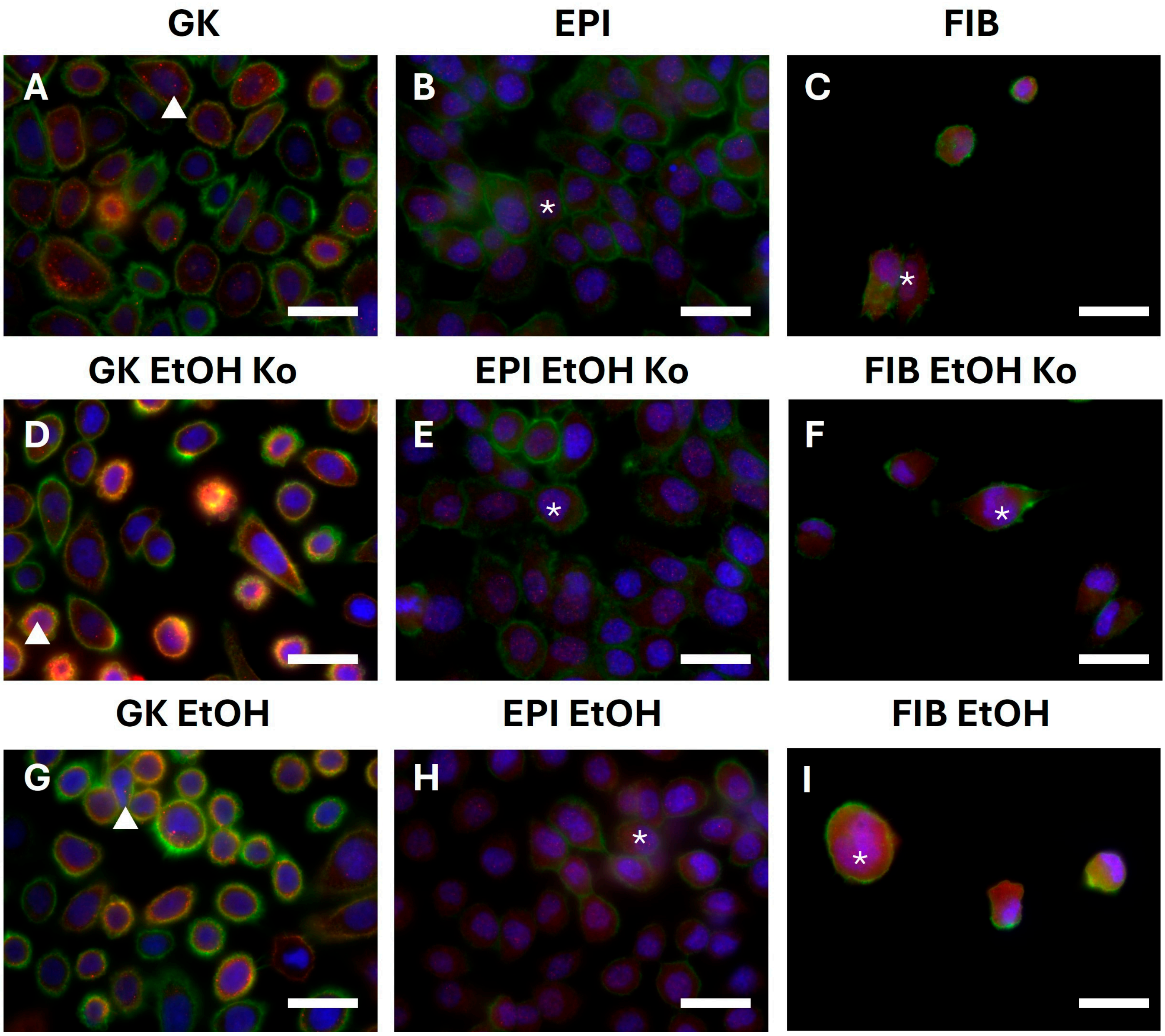

Supplementary Figure S8 Indirect immunofluorescence (IIF) micrographs with specific detection of β-Catenin in GK, EPI and FIB cells as well as their derivatives. The specific protein signal is depicted in red (white arrowheads = specific signal in the cytoplasm; white asterisks = specific signal in nuclei), the cellular actin cytoskeleton in green and cell nuclei in blue. A: GK cells; B: EPI cells; C: FIB cells; D: GK EtOH Ko cells, E: EPI EtOH Ko cells; F: FIB EtOH Ko cells; G: GK EtOH cells; H: EPI EtOH cells; I: FIB EtOH cells. Scale bars = 40 µm.

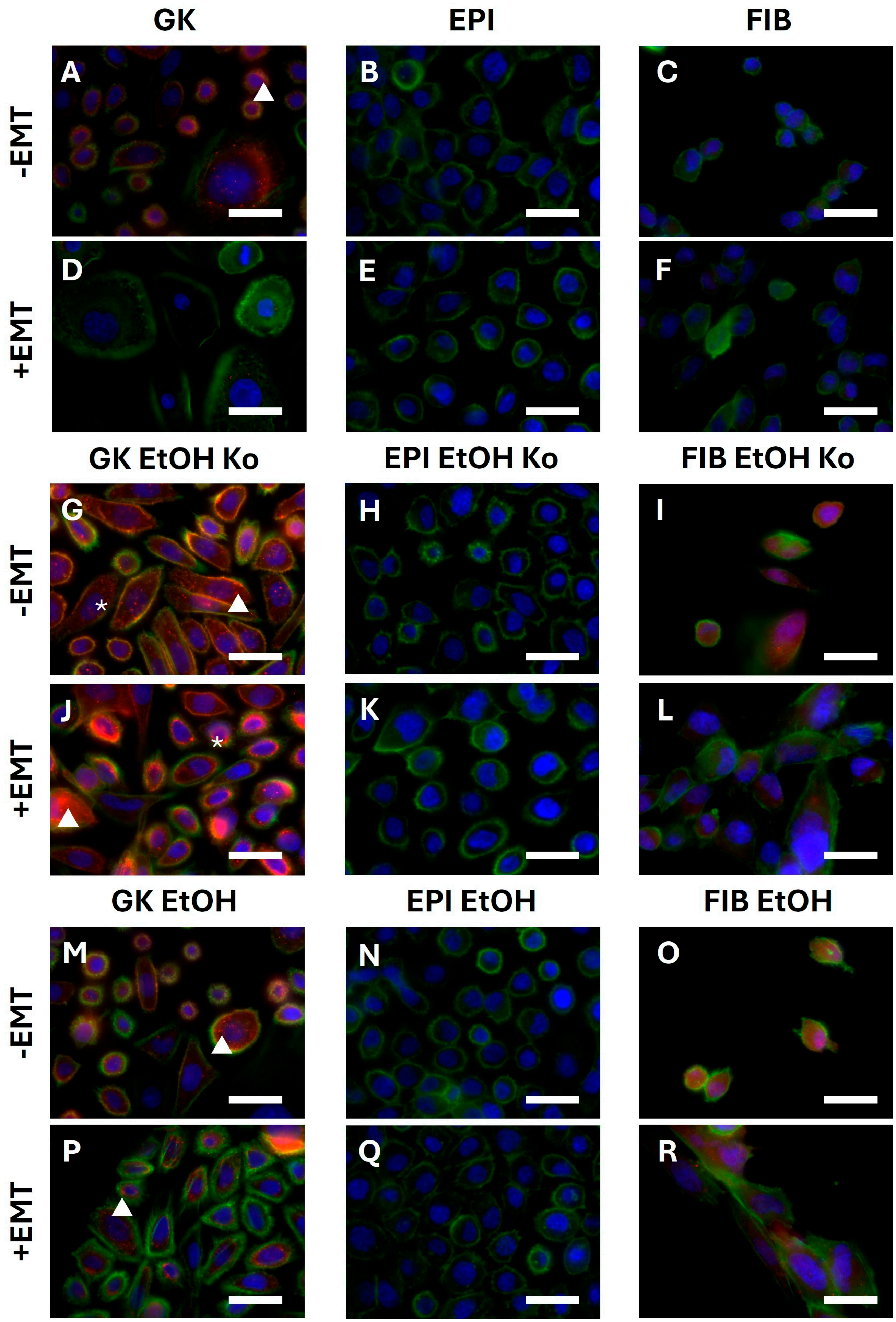
GK
EPI
FIB
-EMT
+EMT
A
B
C
D
E
F
GK EtOH Ko
EPI EtOH Ko
FIB EtOH Ko
G
H
I
J
K
L
GK EtOH
EPI EtOH
FIB EtOH
M
N
O
P
Q
R

Supplementary Figure S9 Indirect immunofluorescence (IIF) micrographs with specific detection of β-Catenin in GK, EPI and FIB cells as well as their derivatives without ("-EMT") and with ("+EMT") EMT-inducing cocktail. The specific protein signal is depicted in red (white arrowheads = specific signal in the cytoplasm; white asterisks = specific signal in nuclei), the cellular actin cytoskeleton in green and cell nuclei in blue. A and D: GK cells; B and E: EPI cells; C and F: FIB cells; G and J: GK EtOH Ko cells, H and K: EPI EtOH Ko cells; I and L: FIB EtOH Ko cells; M and P: GK EtOH cells; N and Q: EPI EtOH cells; O and R: FIB EtOH cells. Scale bars = 40 μm.

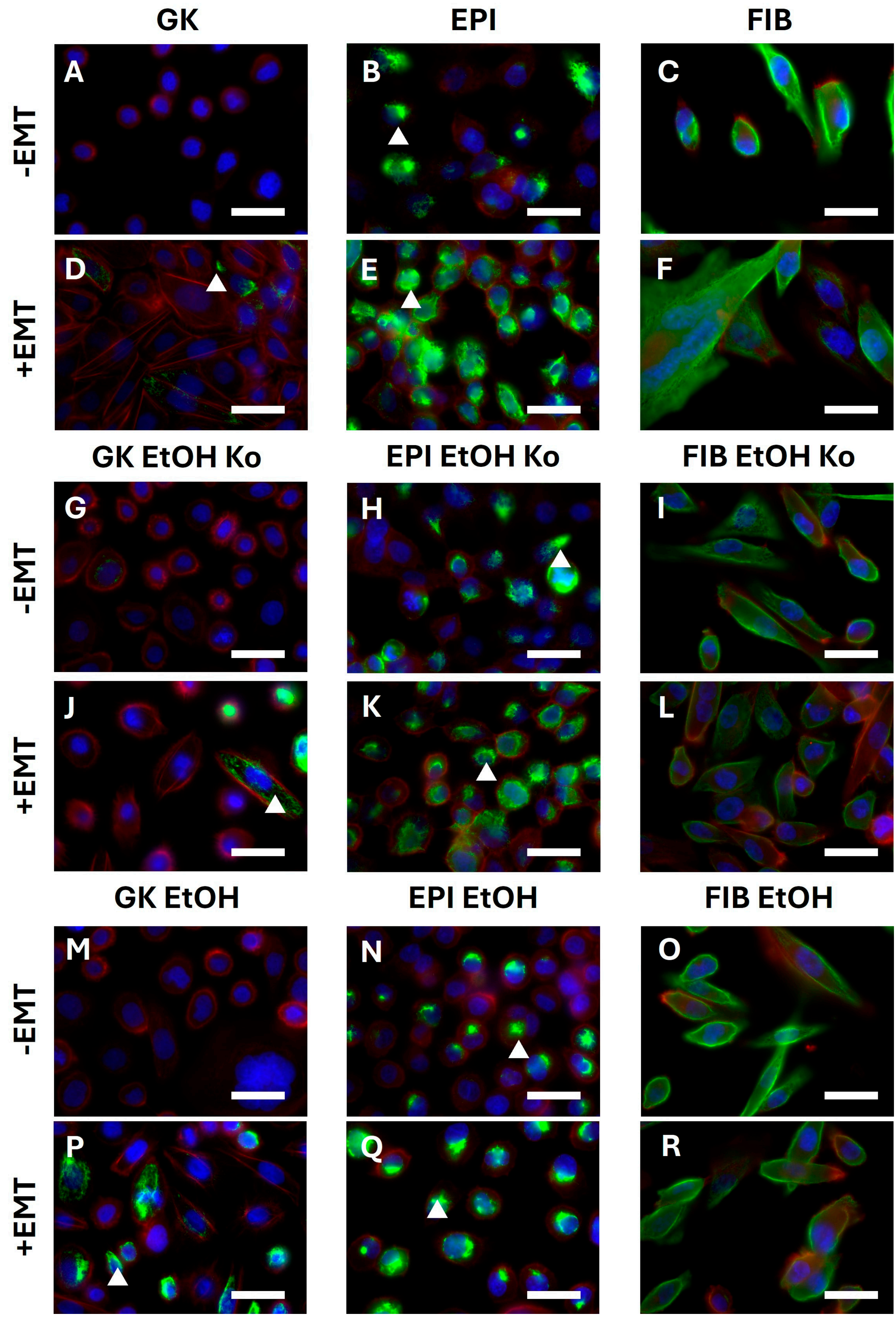
GK
EPI
FIB
-EMT
+EMT
A
B
C
D
E
F
GK EtOH Ko
EPI EtOH Ko
FIB EtOH Ko
G
H
I
J
K
L
GK EtOH
EPI EtOH
FIB EtOH
M
N
O
P
Q
R

Supplementary Figure S10 Indirect immunofluorescence (IIF) micrographs with specific detection of Vimentin in GK, EPI and FIB cells as well as their derivatives without ("-EMT") and with ("+EMT") EMT-inducing cocktail. The specific protein signal is depicted in green (see white arrowheads), the cellular actin cytoskeleton in red and cell nuclei in blue. A and D: GK cells; B and E: EPI cells; C and F: FIB cells; G and J: GK EtOH Ko cells, H and K: EPI EtOH Ko cells; I and L: FIB EtOH Ko cells; M and P: GK EtOH cells; N and Q: EPI EtOH cells; O and R: FIB EtOH cells. Scale bars = 40 µm.

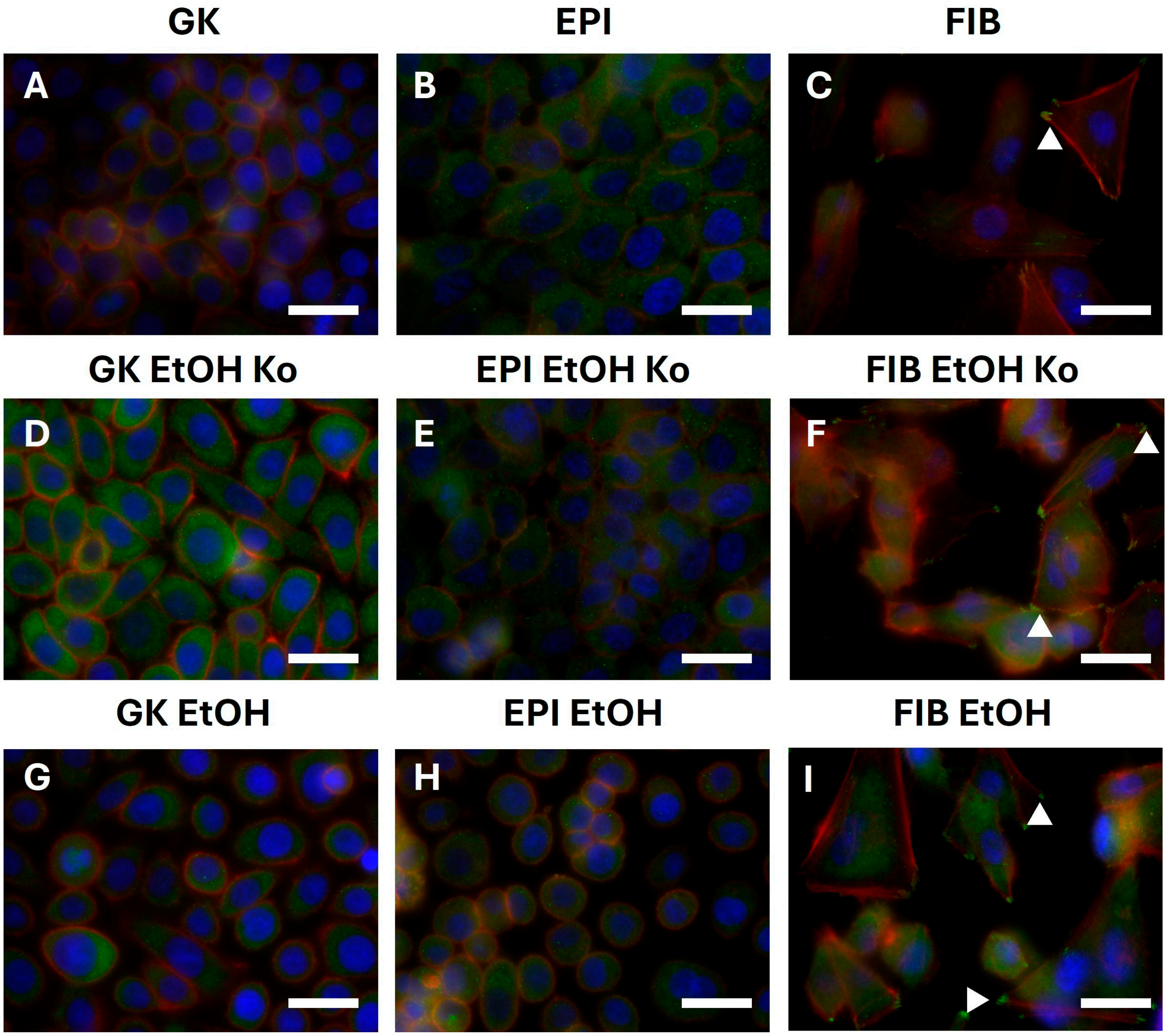

Supplementary Figure S11 Indirect immunofluorescence (IIF) micrographs with specific detection of pFAKY397 in GK, EPI and FIB cells as well as their derivatives. The specific protein signal is depicted in green (see white arrowheads), the cellular actin cytoskeleton in red and cell nuclei in blue. A: GK cells; B: EPI cells; C: FIB cells; D: GK EtOH Ko cells, E: EPI EtOH Ko cells; F: FIB EtOH Ko cells; G: GK EtOH cells; H: EPI EtOH cells; I: FIB EtOH cells. Scale bars = 40 µm.

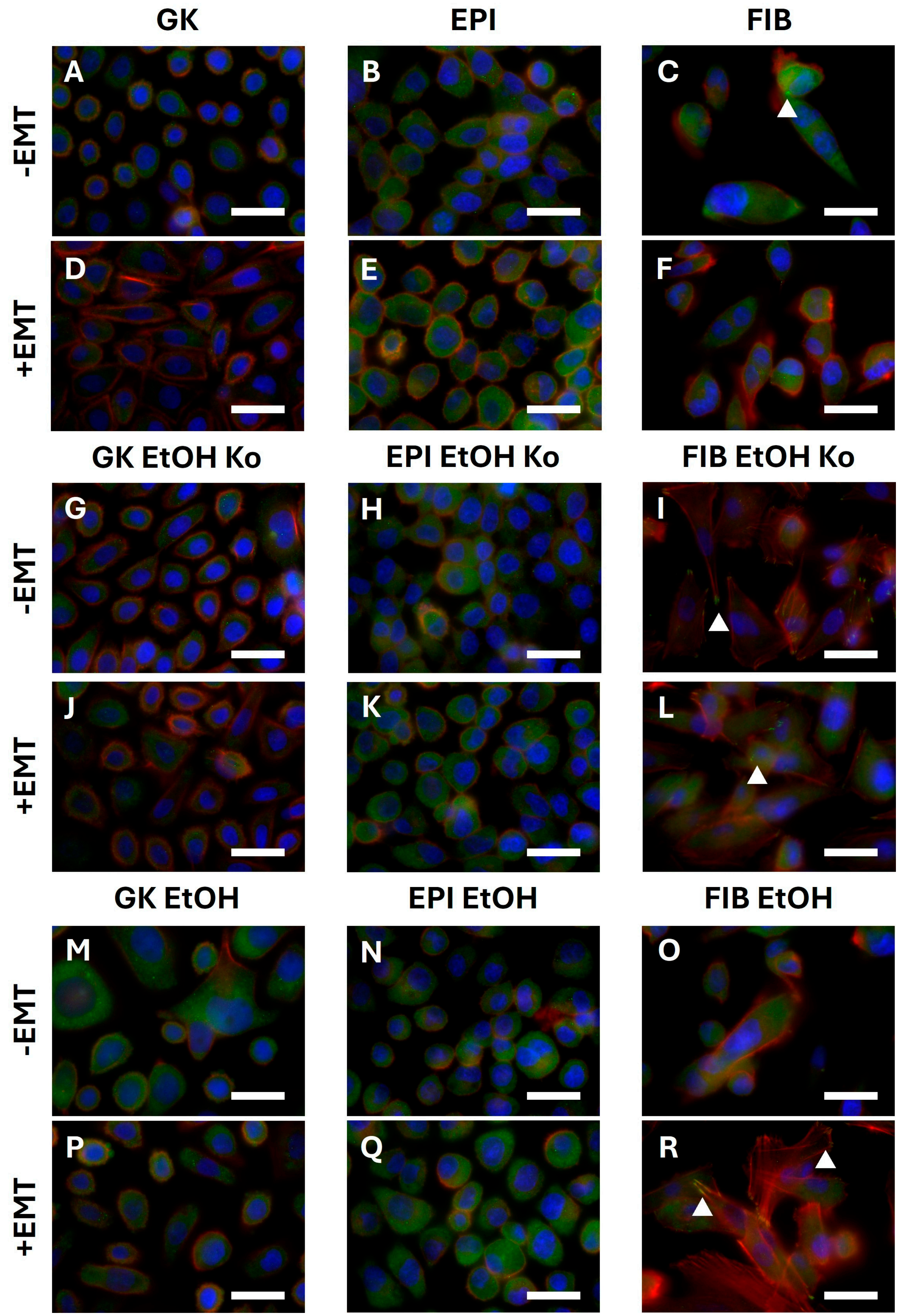
GK
EPI
FIB
-EMT
+EMT
A
B
C
D
E
F
GK EtOH Ko
EPI EtOH Ko
FIB EtOH Ko
G
H
I
J
K
L
GK EtOH
EPI EtOH
FIB EtOH
M
N
O
P
Q
R

Supplementary Figure S12 Indirect immunofluorescence (IIF) micrographs with specific detection of pFAKY397 in GK, EPI and FIB cells as well as their derivatives without ("-EMT") and with ("+EMT") EMT-inducing cocktail. The specific protein signal is depicted in green (see white arrowheads), the cellular actin cytoskeleton in red and cell nuclei in blue. A and D: GK cells; B and E: EPI cells; C and F: FIB cells; G and J: GK EtOH Ko cells, H and K: EPI EtOH Ko cells; I and L: FIB EtOH Ko cells; M and P: GK EtOH cells; N and Q: EPI EtOH cells; O and R: FIB EtOH cells. Scale bars = 40 μm.

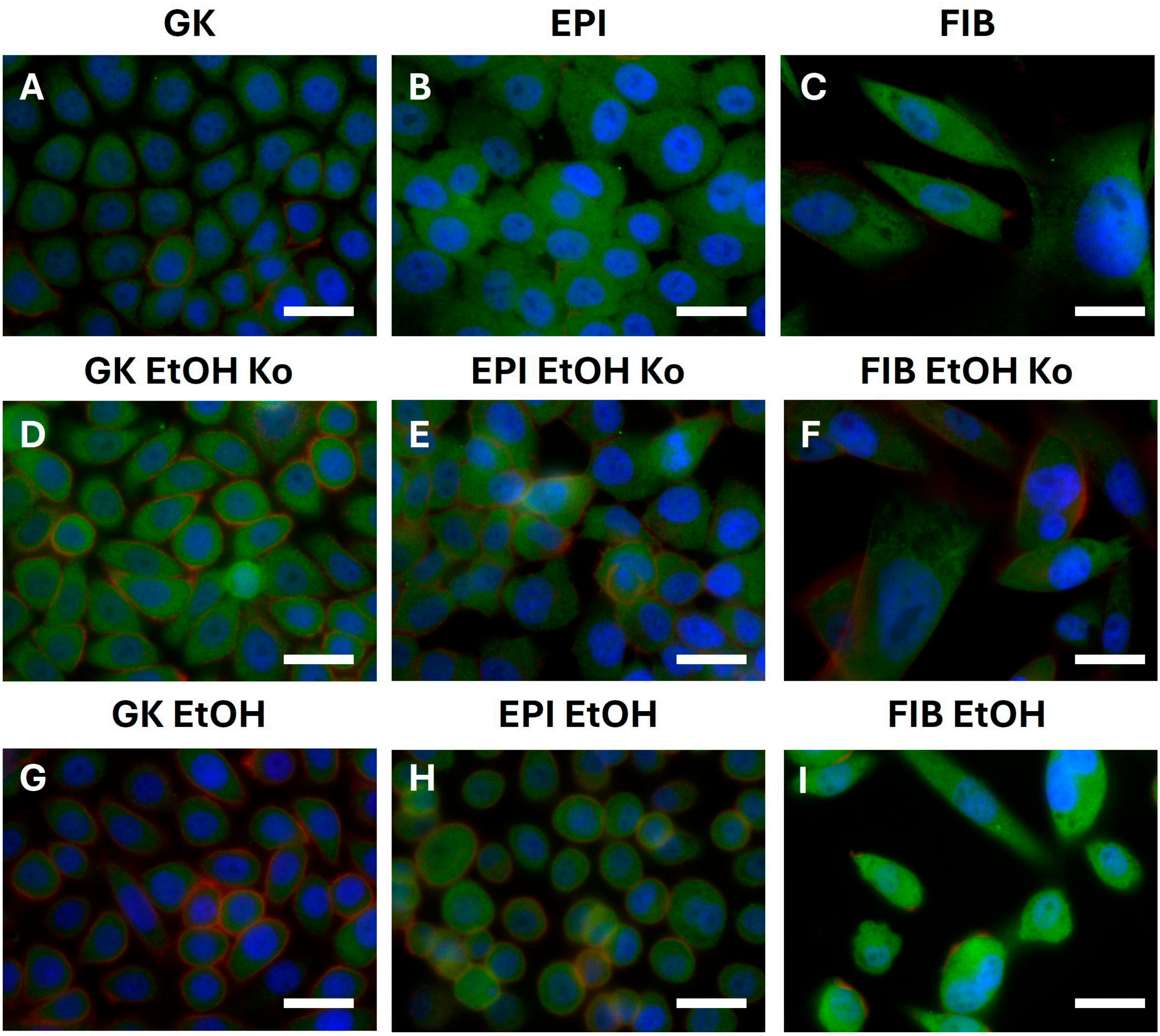

Supplementary Figure S13 Indirect immunofluorescence (IIF) micrographs with specific detection of Merlin in GK, EPI and FIB cells as well as their derivatives. The specific protein signal is depicted in green, the cellular actin cytoskeleton in red and cell nuclei in blue. A: GK cells; B: EPI cells; C: FIB cells; D: GK EtOH Ko cells, E: EPI EtOH Ko cells; F: FIB EtOH Ko cells; G: GK EtOH cells; H: EPI EtOH cells; I: FIB EtOH cells. Scale bars = 40 μm.

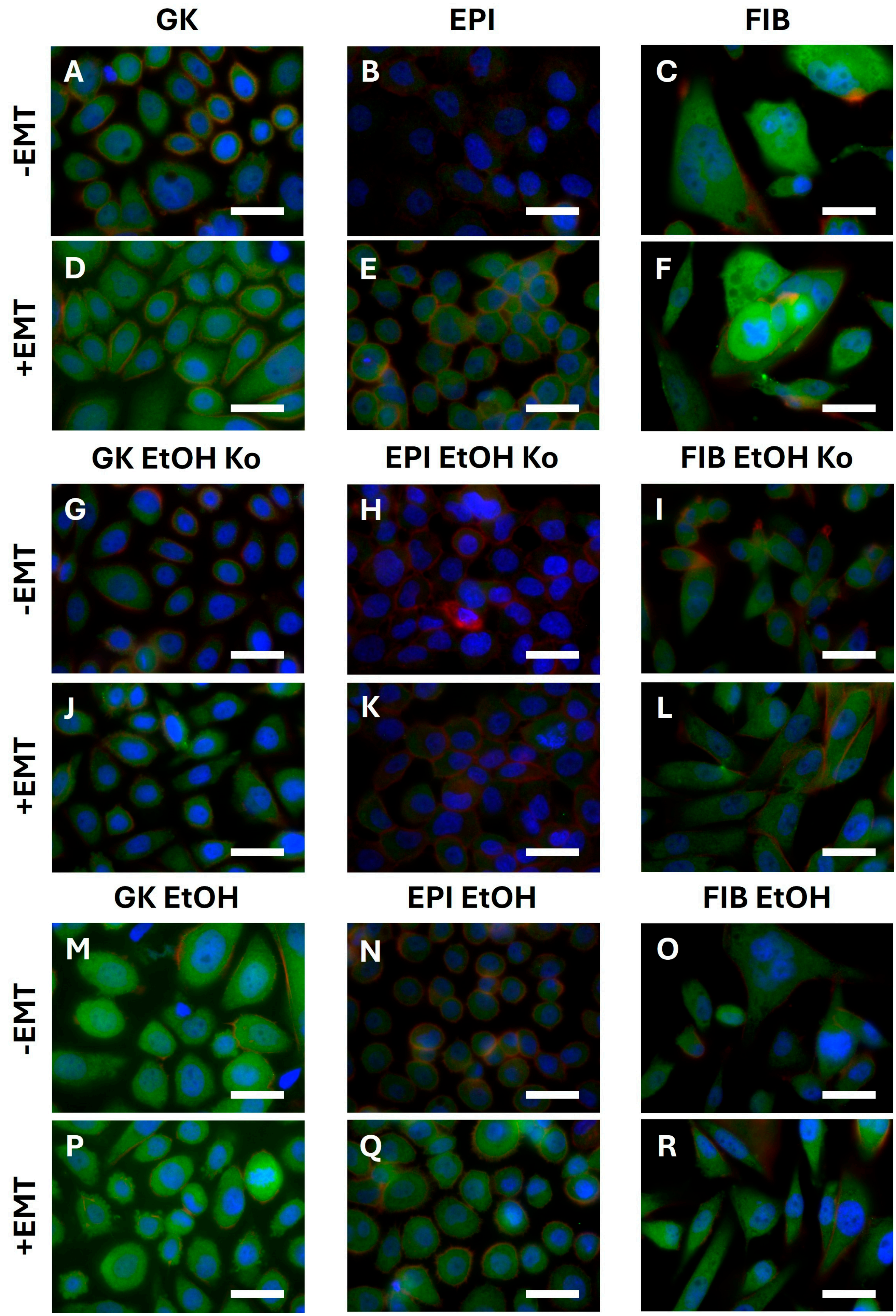
GK
EPI
FIB
A
B
C
-EMT
D
E
F
+EMT
GK EtOH Ko
EPI EtOH Ko
FIB EtOH Ko
G
H
I
-EMT
J
K
L
+EMT
GK EtOH
EPI EtOH
FIB EtOH
M
N
O
-EMT
P
Q
R
+EMT

Supplementary Figure S14 Indirect immunofluorescence (IIF) micrographs with specific detection of Merlin in GK, EPI and FIB cells as well as their derivatives without ("-EMT") and with ("+EMT") EMT-inducing cocktail. The specific protein signal is depicted in green, the cellular actin cytoskeleton in red and cell nuclei in blue. A and D: GK cells; B and E: EPI cells; C and F: FIB cells; G and J: GK EtOH Ko cells, H and K: EPI EtOH Ko cells; I and L: FIB EtOH Ko cells; M and P: GK EtOH cells; N and Q: EPI EtOH cells; O and R: FIB EtOH cells. Scale bars = 40 μm.

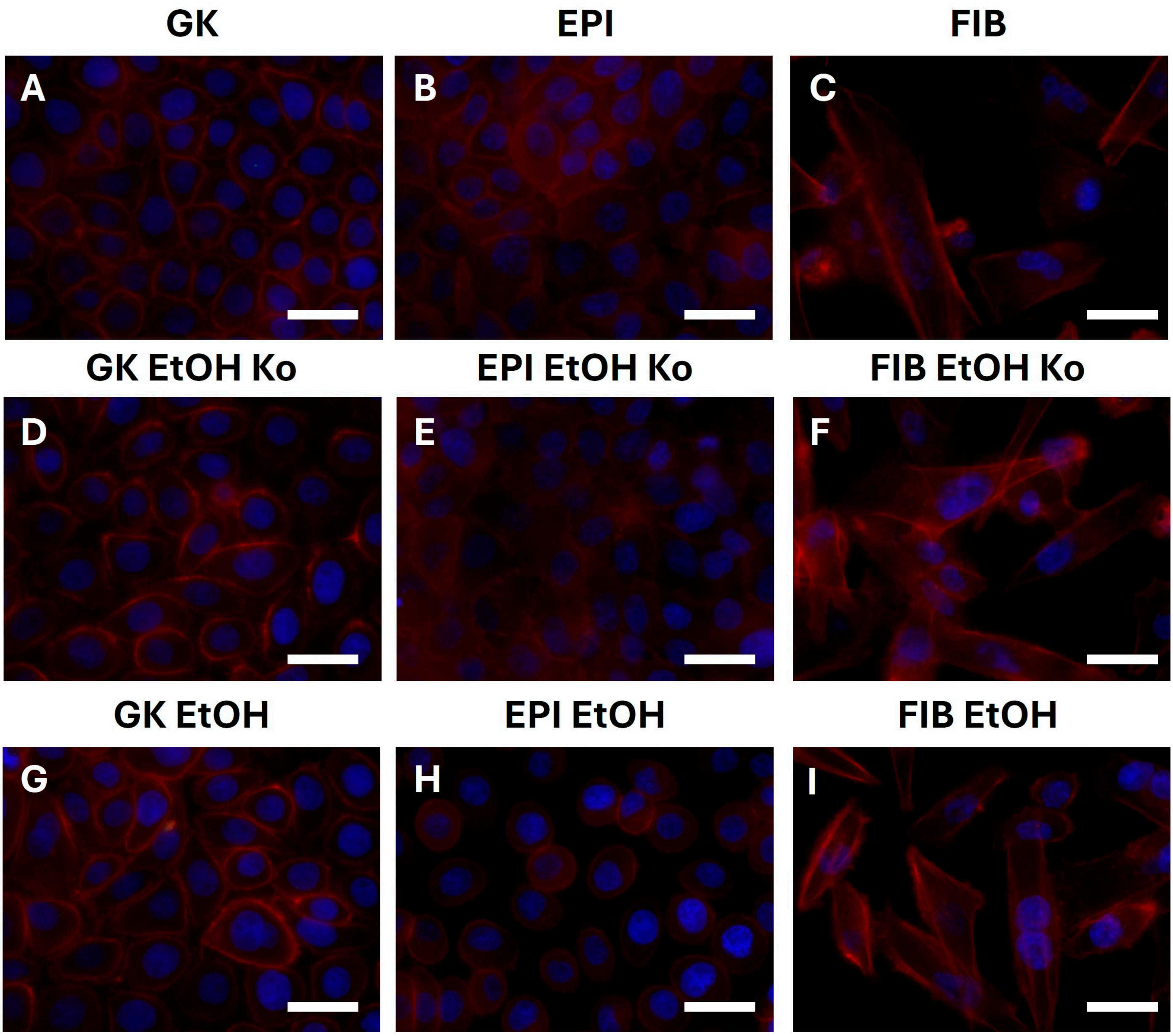

Supplementary Figure S15 Indirect immunofluorescence (IIF) micrographs with specific detection of YAP1 in GK, EPI and FIB cells as well as their derivatives. The specific protein signal is depicted in green, the cellular actin cytoskeleton in red and cell nuclei in blue. A: GK cells; B: EPI cells; C: FIB cells; D: GK EtOH Ko cells, E: EPI EtOH Ko cells; F: FIB EtOH Ko cells; G: GK EtOH cells; H: EPI EtOH cells; I: FIB EtOH cells. Scale bars = 40 μm.

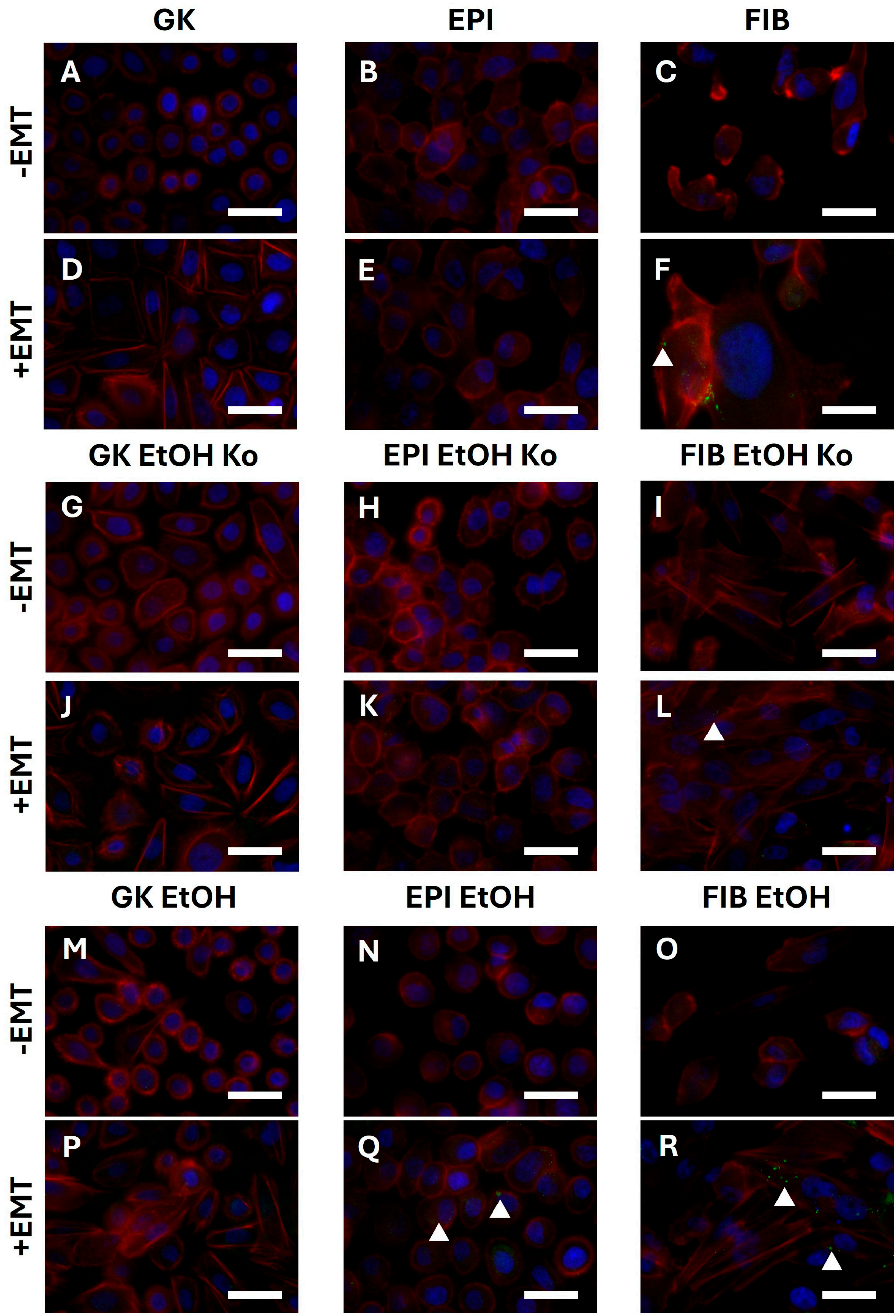

GK
EPI
FIB
-EMT
+EMT
A
B
C
D
E
F
GK EtOH Ko
EPI EtOH Ko
FIB EtOH Ko
G
H
I
J
K
L
GK EtOH
EPI EtOH
FIB EtOH
M
N
O
P
Q
R

Supplementary Figure S16 Indirect immunofluorescence (IIF) micrographs with specific detection of YAP1 in GK, EPI and FIB cells as well as their derivatives without ("-EMT") and with ("+EMT") EMT-inducing cocktail. The specific protein signal is depicted in green (see white arrowheads), the cellular actin cytoskeleton in red and cell nuclei in blue. A and D: GK cells; B and E: EPI cells; C and F: FIB cells; G and J: GK EtOH Ko cells, H and K: EPI EtOH Ko cells; I and L: FIB EtOH Ko cells; M and P: GK EtOH cells; N and Q: EPI EtOH cells; O and R: FIB EtOH cells. Scale bars = 40 µm.

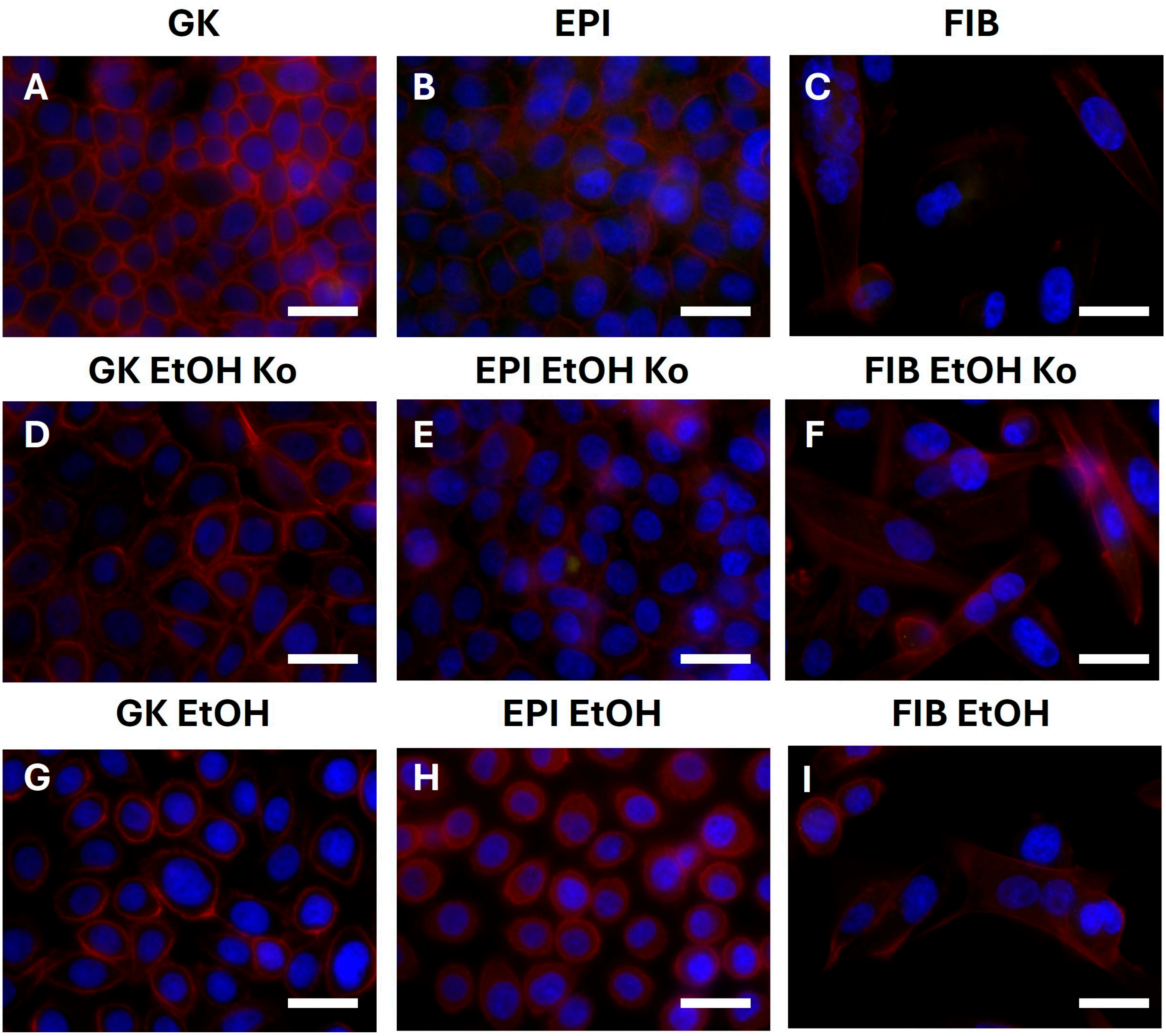

Supplementary Figure S17 Indirect immunofluorescence (IIF) micrographs with specific detection of TAZ in GK, EPI and FIB cells as well as their derivatives. The specific protein signal is depicted in green, the cellular actin cytoskeleton in red and cell nuclei in blue. A: GK cells; B: EPI cells; C: FIB cells; D: GK EtOH Ko cells, E: EPI EtOH Ko cells; F: FIB EtOH Ko cells; G: GK EtOH cells; H: EPI EtOH cells; I: FIB EtOH cells. Scale bars = 40 µm.

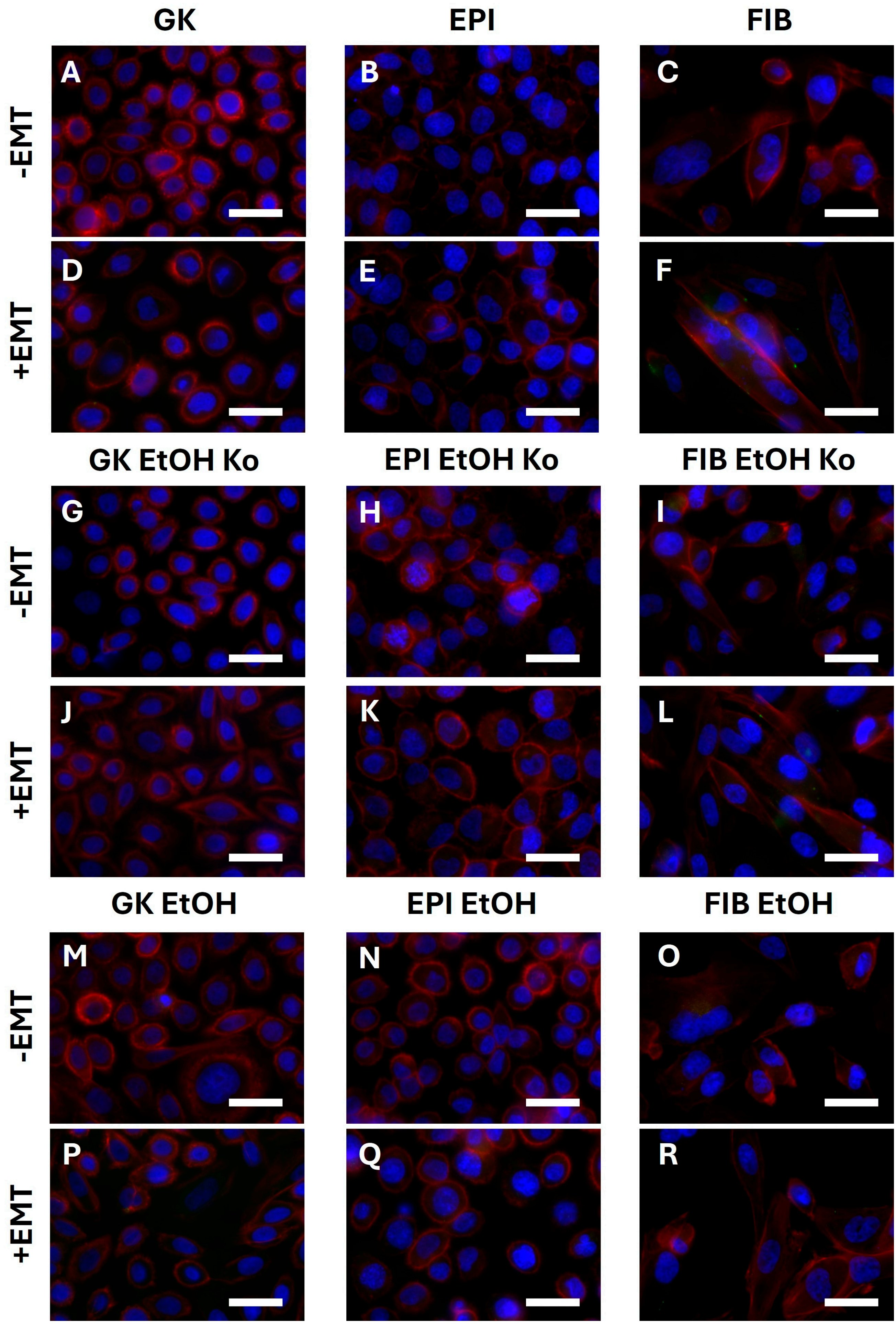
GK
EPI
FIB
A
B
C
-EMT
D
E
F
+EMT
GK EtOH Ko
EPI EtOH Ko
FIB EtOH Ko
G
H
I
-EMT
J
K
L
+EMT
GK EtOH
EPI EtOH
FIB EtOH
M
N
O
-EMT
P
Q
R
+EMT

Supplementary Figure S18 Indirect immunofluorescence (IIF) micrographs with specific detection of TAZ in GK, EPI and FIB cells as well as their derivatives without ("-EMT") and with ("+EMT") EMT-inducing cocktail. The specific protein signal is depicted in green, the cellular actin cytoskeleton in red and cell nuclei in blue. A and D: GK cells; B and E: EPI cells; C and F: FIB cells; G and J: GK EtOH Ko cells, H and K: EPI EtOH Ko cells; I and L: FIB EtOH Ko cells; M and P: GK EtOH cells; N and Q: EPI EtOH cells; O and R: FIB EtOH cells. Scale bars = 40 µm.

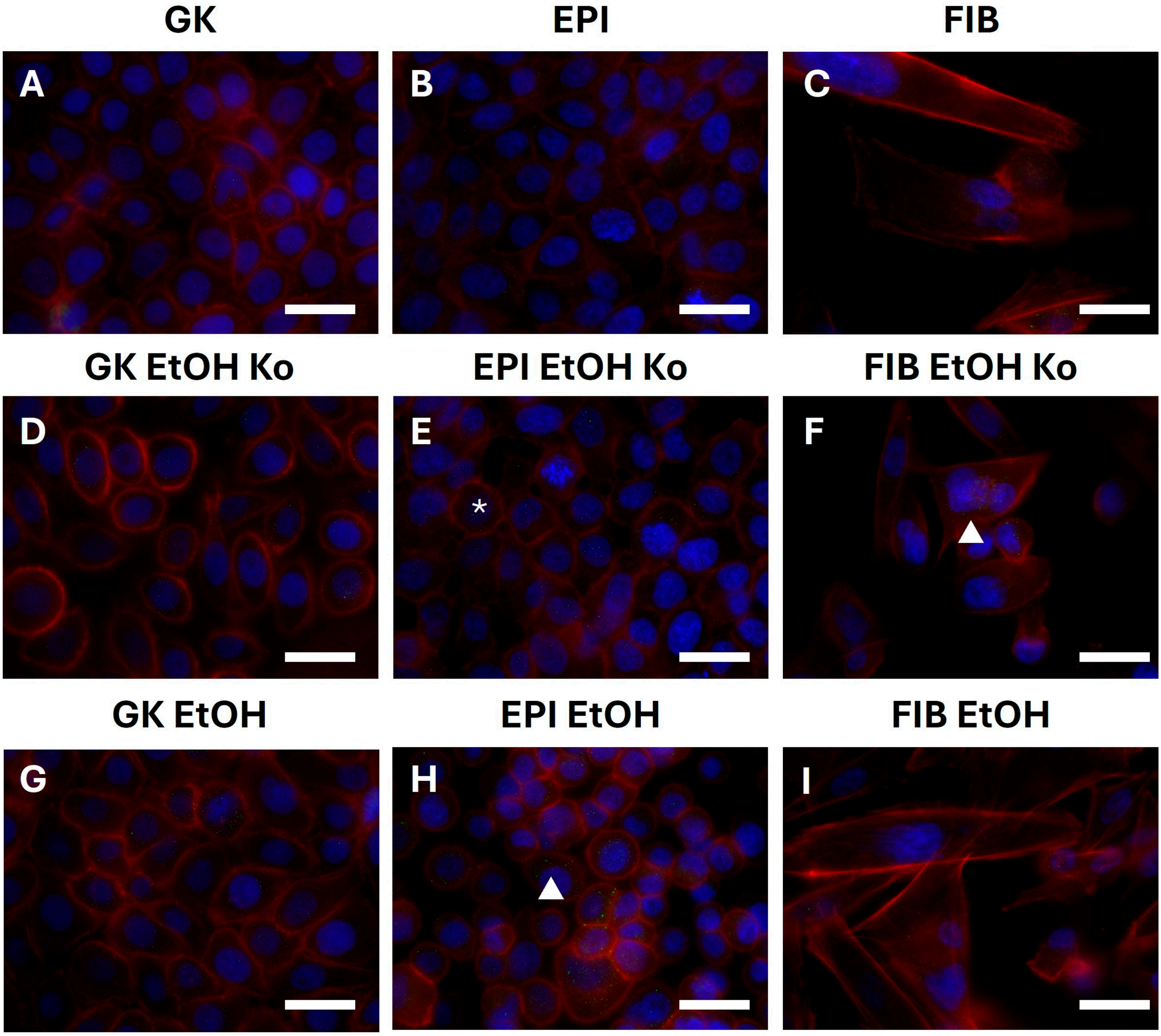

Supplementary Figure S19 Indirect immunofluorescence (IIF) micrographs with specific detection of TEAD1 in GK, EPI and FIB cells as well as their derivatives. The specific protein signal is depicted in green (white arrowheads = specific signal in the cytoplasm; white asterisks = specific signal in nuclei), the cellular actin cytoskeleton in red and cell nuclei in blue. A: GK cells; B: EPI cells; C: FIB cells; D: GK EtOH Ko cells, E: EPI EtOH Ko cells; F: FIB EtOH Ko cells; G: GK EtOH cells; H: EPI EtOH cells; I: FIB EtOH cells. Scale bars = 40 µm.

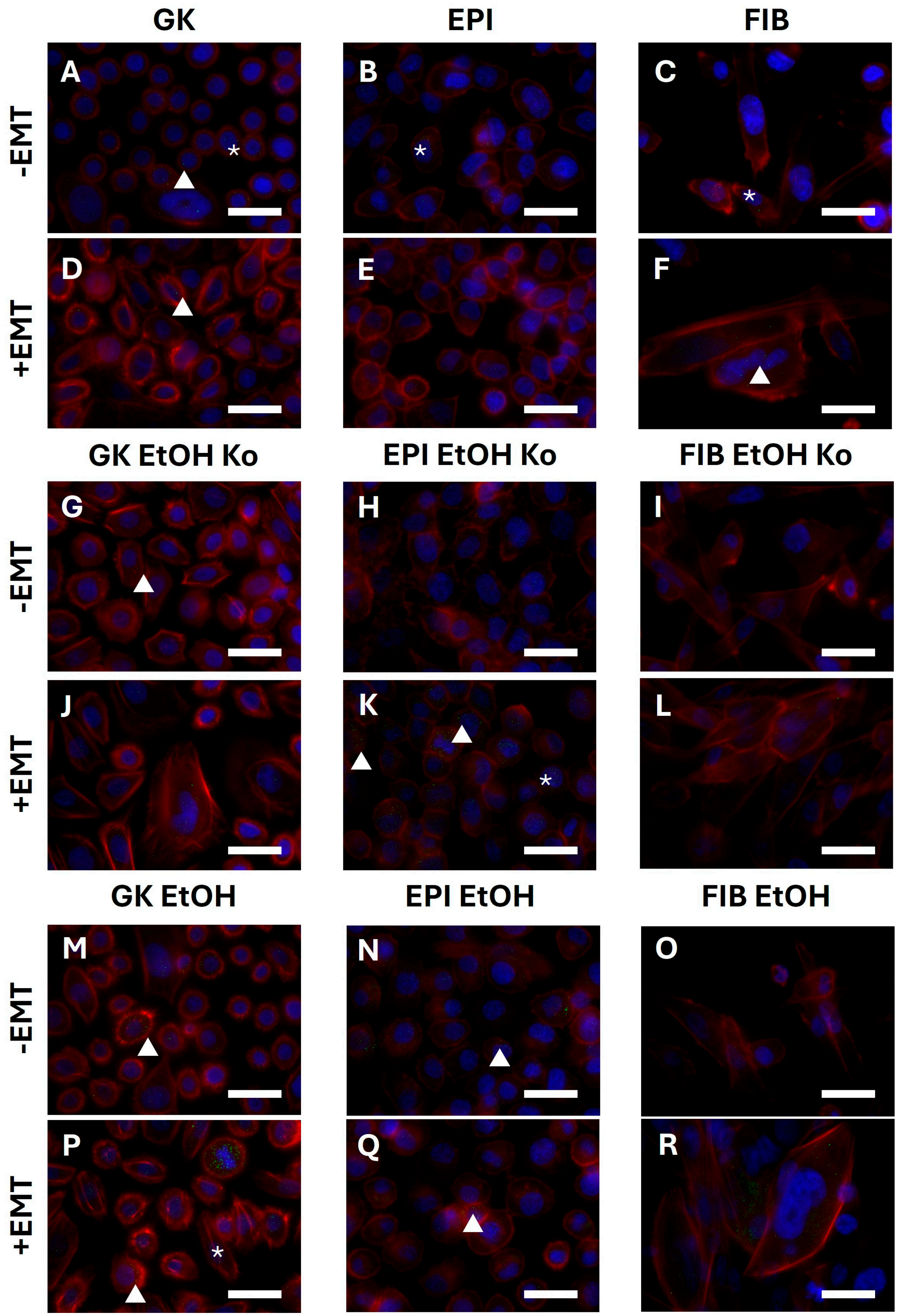
GK
EPI
FIB
-EMT
+EMT
A
B
C
D
E
F
GK EtOH Ko
EPI EtOH Ko
FIB EtOH Ko
G
H
I
J
K
L
GK EtOH
EPI EtOH
FIB EtOH
M
N
O
P
Q
R

Supplementary Figure S20 Indirect immunofluorescence (IIF) micrographs with specific detection of TEAD1 in GK, EPI and FIB cells as well as their derivatives without ("-EMT") and with ("+EMT") EMT-inducing cocktail. The specific protein signal is depicted in green (white arrowheads = specific signal in the cytoplasm; white asterisks = specific signal in nuclei), the cellular actin cytoskeleton in red and cell nuclei in blue. A and D: GK cells; B and E: EPI cells; C and F: FIB cells; G and J: GK EtOH Ko cells, H and K: EPI EtOH Ko cells; I and L: FIB EtOH Ko cells; M and P: GK EtOH cells; N and Q: EPI EtOH cells; O and R: FIB EtOH cells. Scale bars = 40 µm.

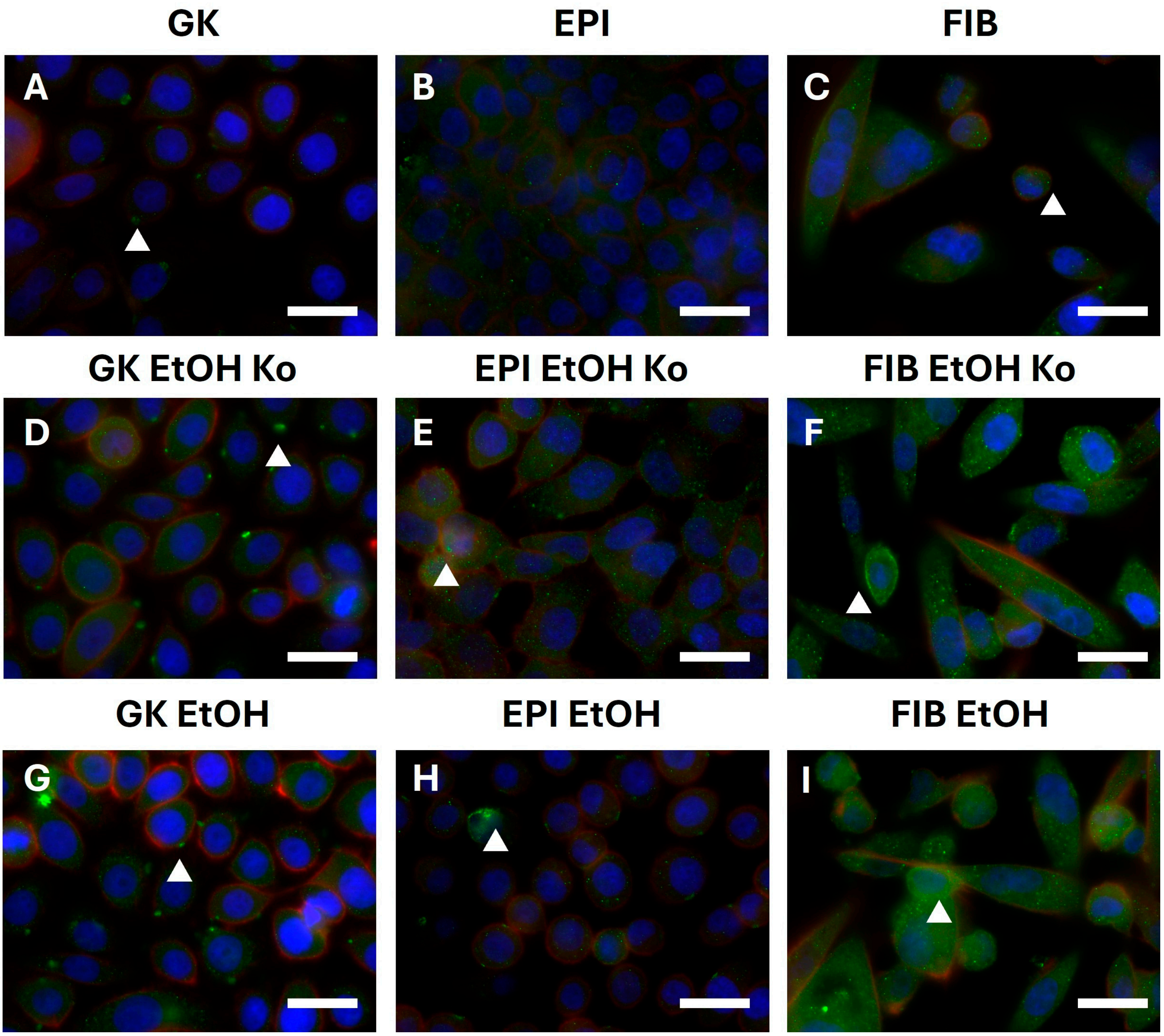

Supplementary Figure S21 Indirect immunofluorescence (IIF) micrographs with specific detection of Oct4 in GK, EPI and FIB cells as well as their derivatives. The specific protein signal is depicted in green (see white arrowheads), the cellular actin cytoskeleton in red and cell nuclei in blue. A: GK cells; B: EPI cells; C: FIB cells; D: GK EtOH Ko cells, E: EPI EtOH Ko cells; F: FIB EtOH Ko cells; G: GK EtOH cells; H: EPI EtOH cells; I: FIB EtOH cells. Scale bars = 40 µm.

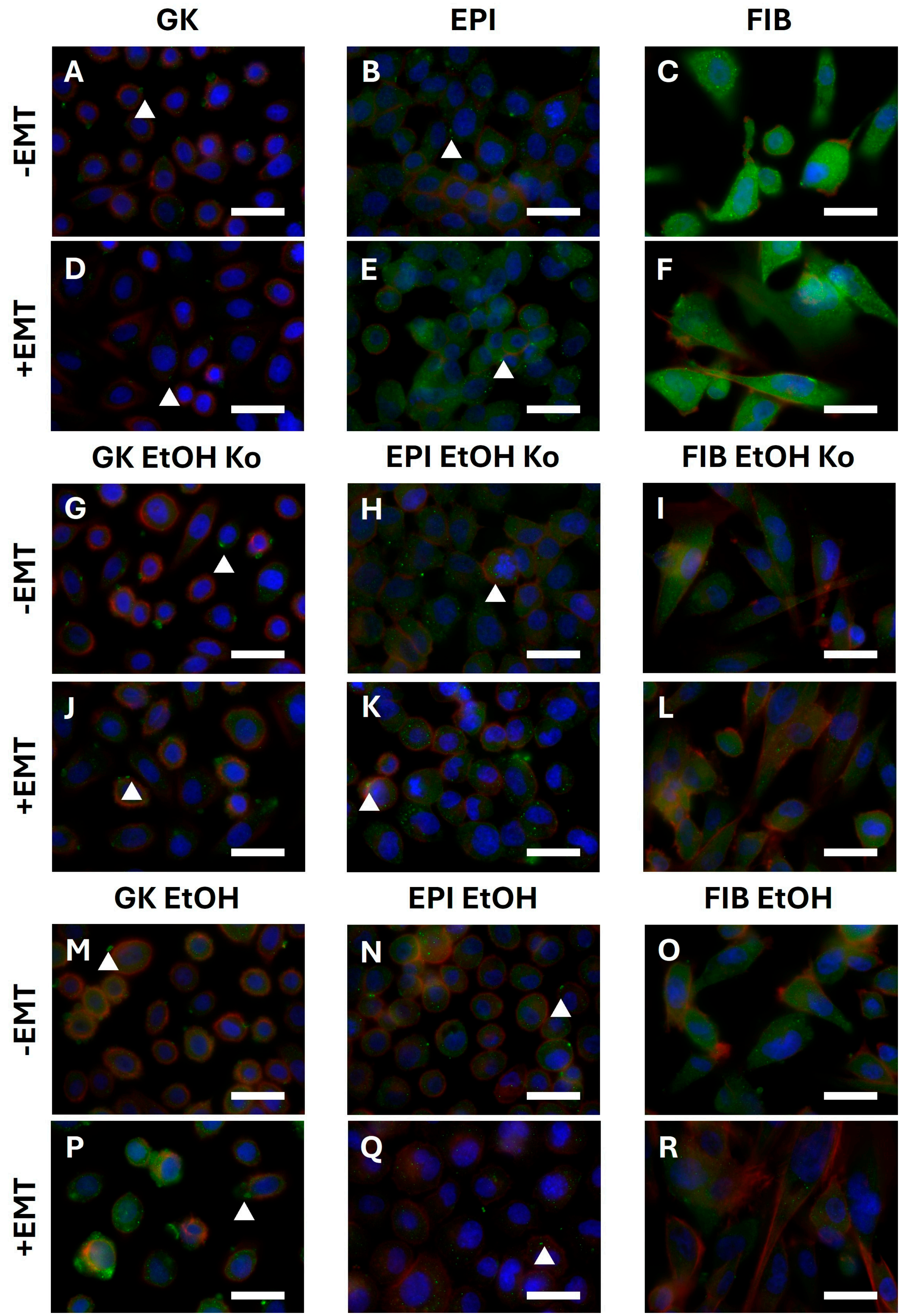
GK
EPI
FIB
-EMT
+EMT
A
B
C
D
E
F
GK EtOH Ko
EPI EtOH Ko
FIB EtOH Ko
G
H
I
J
K
L
GK EtOH
EPI EtOH
FIB EtOH
M
N
O
P
Q
R

Supplementary Figure S22 Indirect immunofluorescence (IIF) micrographs with specific detection of Oct4 in GK, EPI and FIB cells as well as their derivatives without ("-EMT") and with ("+EMT") EMT-inducing cocktail. The specific protein signal is depicted in green (see white arrowheads), the cellular actin cytoskeleton in red and cell nuclei in blue. A and D: GK cells; B and E: EPI cells; C and F: FIB cells; G and J: GK EtOH Ko cells, H and K: EPI EtOH Ko cells; I and L: FIB EtOH Ko cells; M and P: GK EtOH cells; N and Q: EPI EtOH cells; O and R: FIB EtOH cells. Scale bars = 40 µm.

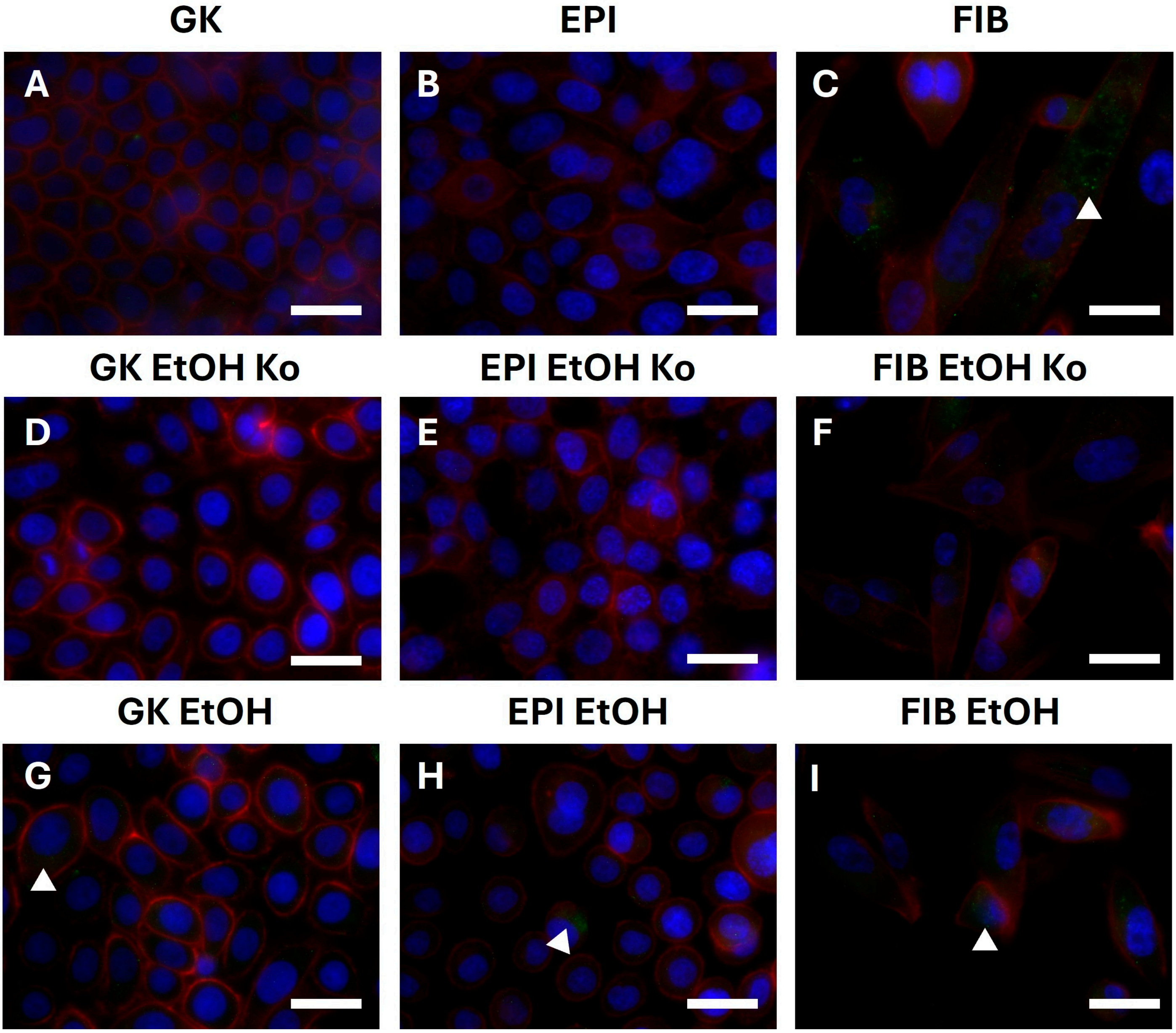

Supplementary Figure S23 Indirect immunofluorescence (IIF) micrographs with specific detection of ZEB1 in GK, EPI and FIB cells as well as their derivatives. The specific protein signal is depicted in green (see white arrowheads), the cellular actin cytoskeleton in red and cell nuclei in blue. A: GK cells; B: EPI cells; C: FIB cells; D: GK EtOH Ko cells, E: EPI EtOH Ko cells; F: FIB EtOH Ko cells; G: GK EtOH cells; H: EPI EtOH cells; I: FIB EtOH cells. Scale bars = 40 µm.

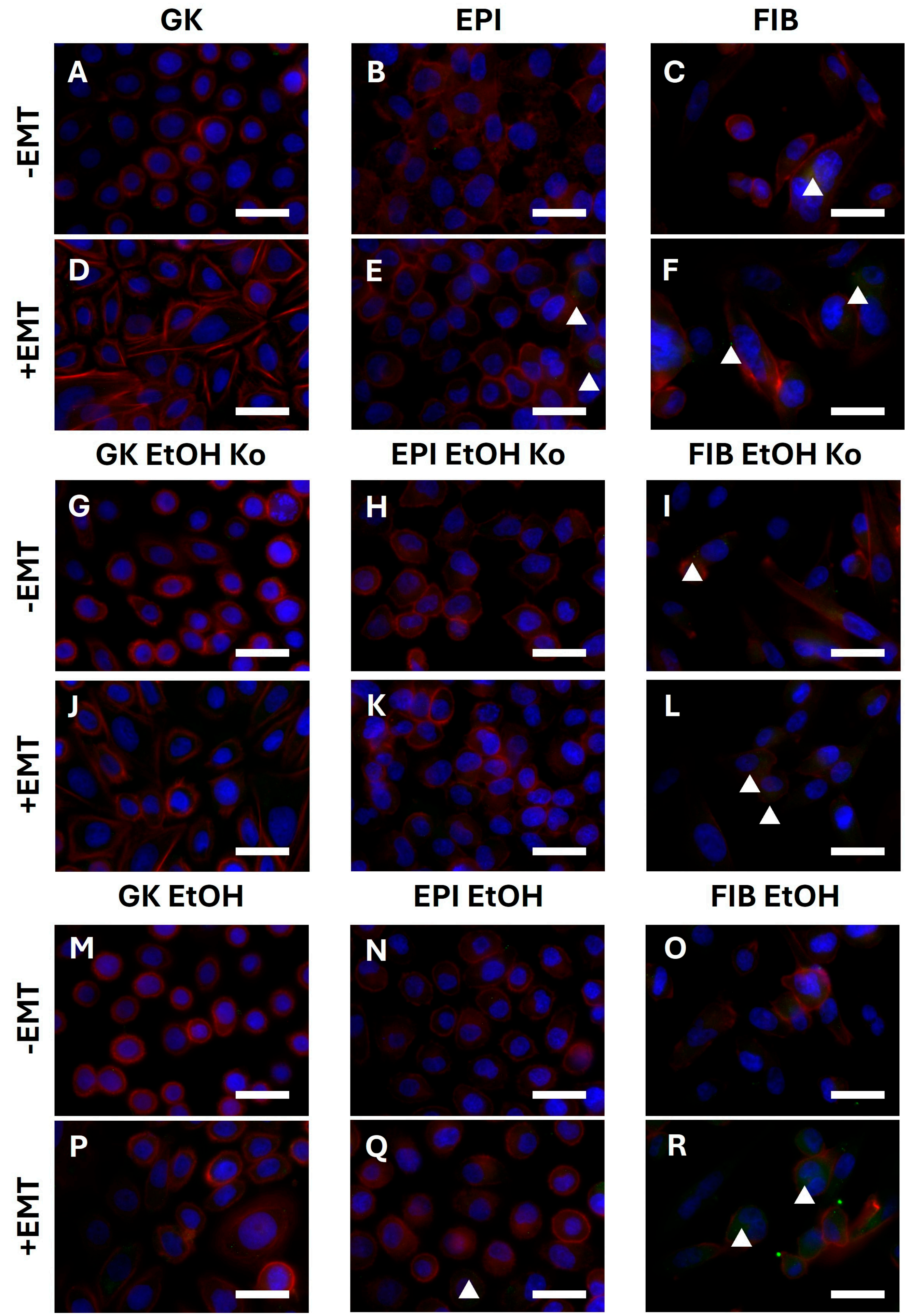
GK
EPI
FIB
A
B
C
D
E
F
-EMT
+EMT
GK EtOH Ko
EPI EtOH Ko
FIB EtOH Ko
G
H
I
J
K
L
-EMT
+EMT
GK EtOH
EPI EtOH
FIB EtOH
M
N
O
P
Q
R
-EMT
+EMT

Supplementary Figure S24 Indirect immunofluorescence (IIF) micrographs with specific detection of ZEB1 in GK, EPI and FIB cells as well as their derivatives without ("-EMT") and with ("+EMT") EMT-inducing cocktail. The specific protein signal is depicted in green (see white arrowheads), the cellular actin cytoskeleton in red and cell nuclei in blue. A and D: GK cells; B and E: EPI cells; C and F: FIB cells; G and J: GK EtOH Ko cells, H and K: EPI EtOH Ko cells; I and L: FIB EtOH Ko cells; M and P: GK EtOH cells; N and Q: EPI EtOH cells; O and R: FIB EtOH cells. Scale bars = 40 μm.

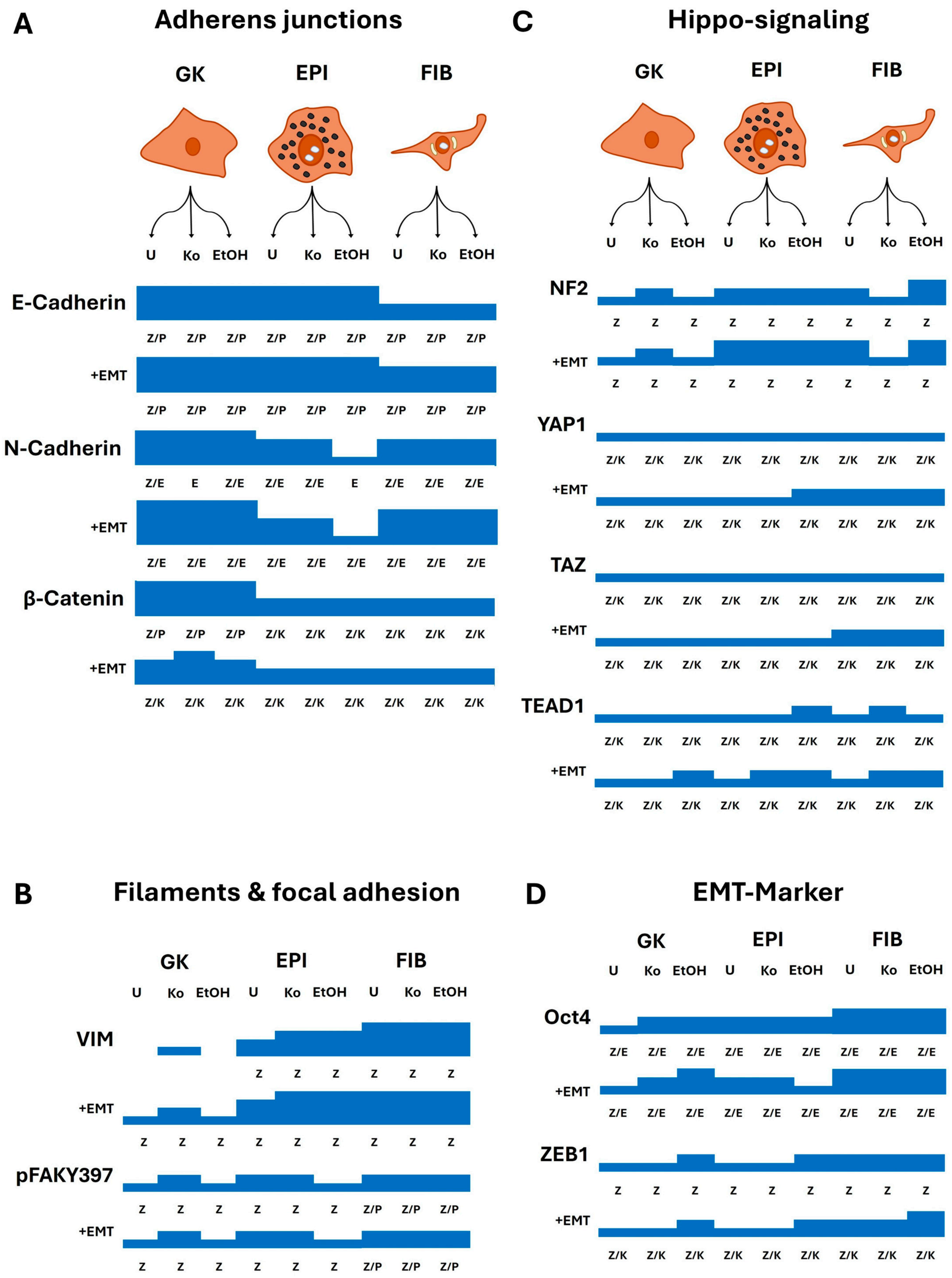
A
Adherens junctions
GK
EPI
FIB
U
Ko
EtOH
E-Cadherin
+EMT
N-Cadherin
β-Catenin
Z/P
Z/E
E
Z/K
C
Hippo-signaling
NF2
YAP1
TAZ
TEAD1
Z
B
Filaments & focal adhesion
VIM
pFAKY397
D
EMT-Marker
Oct4
ZEB1

Supplementary Figure S25 Schematic summary of the effects of cell culture conditions, EtOH-treatment and "+EMT"-treatment on protein amounts and localization in GK, EPI and FIB cells and their derivatives as detected by indirect immunofluorescence (IIF). U = untreated cell line; EtOH = EtOH-treatment; Ko = control culture condition; +EMT = treated with EMT-inducing medium; Z = localization in the cytoplasm; P = perimembranous protein localization; E = localization in inclusion bodies; K = nuclear localization. For color/symbol legend see Figure 1. The upper blue bars indicate the relative amounts of the respective proteins in comparison with GK cells (higher bars indicate a higher protein amount). The lower blue bars represent the direct comparison of protein amounts in the respective cell line with and without addition of the EMT cocktail. A: Analysis of marker proteins belonging to adherens junctions (AJs): E-Cadherin, N-Cadherin and β-Catenin. B: Analysis of marker proteins of the filament system and focal adhesions: Vimentin and pFAKY397. C: Analysis of marker proteins belonging to the Hippo-signaling pathway: Merlin (NF2), YAP1, TAZ and TEAD1. D: Analysis of EMT-markers: Oct4 and ZEB1. Created in BioRender. Steinberg, T. (2025) https://BioRender.com/dnuzqjm

Supplementary Table S24 Statistical analyses (p-values) concerning the ROS production in cells without experimental EMT induction (n.s. = not significant)

ROS-Assay	GK	GK EtOH Ko	GK EtOH	EPI	EPI EtOH Ko	EPI EtOH	FIB	FIB EtOH Ko	FIB EtOH
**GK**		n.s.	n.s.	n.s.	n.s.	<0,0001	0.002	<0,0001	<0,0001
**GK EtOH Ko**	n.s.		n.s.	n.s.	n.s.	0.0023	n.s.	<0,0001	<0,0001
**GK EtOH**	n.s.	n.s.		n.s.	n.s.	0.0029	n.s.	<0,0001	<0,0001
**EPI**	n.s.	n.s.	n.s.		n.s.	0.0494	n.s.	<0,0001	<0,0001
**EPI EtOH Ko**	n.s.	n.s.	n.s.	n.s.		0.0362	n.s.	<0,0001	<0,0001
**EPI EtOH**	<0,0001	0.0023	0.0029	0.0494	0.0362		n.s.	<0,0001	<0,0001
**FIB**	0.002	n.s.	n.s.	n.s.	n.s.	n.s.		<0,0001	<0,0001
**FIB EtOH Ko**	<0,0001	<0,0001	<0,0001	<0,0001	<0,0001	<0,0001	<0,0001		n.s.
**FIB EtOH**	<0,0001	<0,0001	<0,0001	<0,0001	<0,0001	<0,0001	<0,0001	n.s.	

Supplementary Table S25 Statistical analyses (p-values) concerning the ROS production in untreated ("-EMT") and treated ("+EMT") cells

p-value	GK -EMT vs. GK +EMT	GK EtOH Ko -EMT vs. GK EtOH Ko +EMT	GK EtOH -EMT vs. GK EtOH +EMT	EPI -EMT vs. EPI +EMT	EPI EtOH Ko -EMT vs. EPI EtOH Ko +EMT	EPI EtOH -EMT vs. EPI EtOH +EMT	FIB -EMT vs. FIB +EMT	FIB EtOH Ko -EMT vs. FIB EtOH Ko +EMT	FIB EtOH -EMT vs. FIB EtOH +EMT
**ROS**	0.3899	0.0062	0.0324	0.677	0.252	0.4878	0.2555	0.3144	0.0051

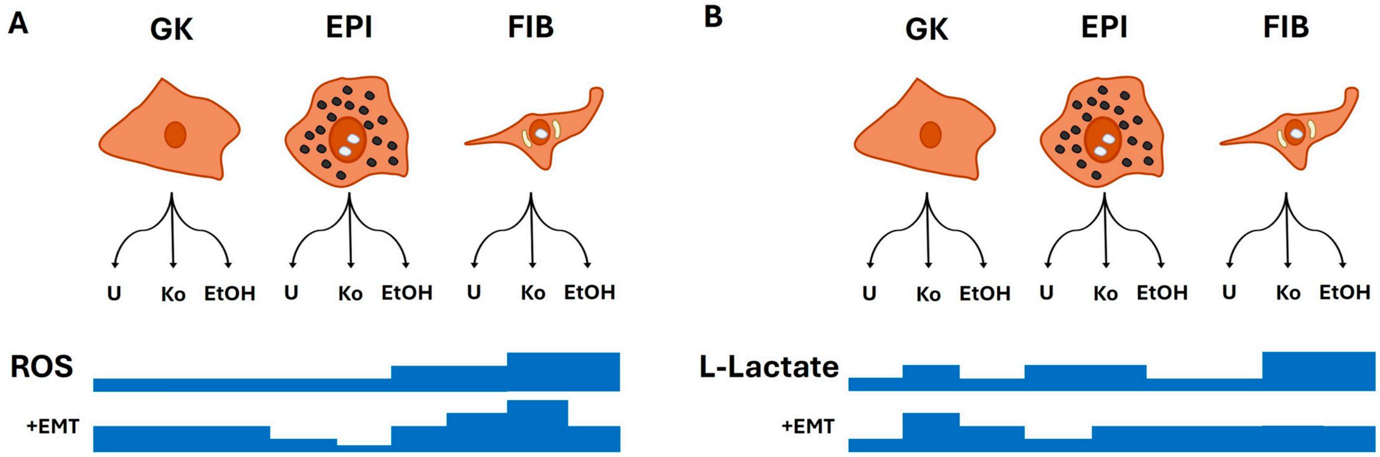

Supplementary Figure S26 Schematic summary of the effects of cell culture conditions, EtOH-treatment and "+EMT"-treatment on cellular ROS and L-lactate production in GK, EPI and FIB cells and their derivatives. U = untreated cell line; EtOH = EtOH-treatment; Ko = control culture condition; +EMT = treated with EMT-inducing medium. For color/symbol legend see Figure 1. The upper blue bars indicate the relative amounts of ROS (A) and L-lactate (B), respectively, in comparison with GK cells (higher bars indicate higher metabolite amounts). The lower blue bars represent the direct comparison of ROS (A) and L-lactate (B) amounts in the respective cell line with and without addition of the EMT cocktail. Created in BioRender. Steinberg, T. (2025) https://BioRender.com/uqnhe16

Supplementary Table S26 Statistical analyses (p-values) concerning the L-lactate production in cells without experimental EMT induction (n.s. = not significant)

L-Lactate	GK	GK EtOH Ko	GK EtOH	EPI	EPI EtOH Ko	EPI EtOH	FIB	FIB EtOH Ko	FIB EtOH
GK		0.0158	n.s.	0.0009	0.0016	n.s.	n.s.	<0,0001	<0,0001
GK EtOH Ko	0.0158		n.s.	n.s.	n.s.	n.s.	n.s.	n.s.	0.0002
GK EtOH	n.s.	n.s.		0.0225	0.0375	n.s.	n.s.	0.0008	<0,0001
EPI	0.0009	n.s.	0.0225		n.s.	n.s.	n.s.	n.s.	0.0033
EPI EtOH Ko	0.0016	n.s.	0.0375	n.s.		n.s.	n.s.	n.s.	0.0019
EPI EtOH	n.s.	n.s.	n.s.	n.s.	n.s.		n.s.	0.0027	<0,0001
FIB	n.s.	n.s.	n.s.	n.s.	n.s.	n.s.		0.0443	0.0003
FIB EtOH Ko	<0,0001	n.s.	0.0008	n.s.	n.s.	0.0027	0.0443		n.s.
FIB EtOH	<0,0001	0.0002	<0,0001	0.0033	0.0019	<0,0001	0.0003	n.s.	

Supplementary Table S27 Statistical analyses (p-values) concerning the L-lactate production in untreated ("-EMT") and treated ("+EMT") cells

p-value	GK -EMT vs. GK +EMT	GK EtOH Ko -EMT vs. GK EtOH Ko +EMT	GK EtOH -EMT vs. GK EtOH +EMT	EPI -EMT vs. EPI +EMT	EPI EtOH Ko -EMT vs. EPI EtOH Ko +EMT	EPI EtOH -EMT vs. EPI EtOH +EMT	FIB -EMT vs. FIB +EMT	FIB EtOH Ko -EMT vs. FIB EtOH Ko +EMT	FIB EtOH -EMT vs. FIB EtOH +EMT
L-Lactate	0.5793	0.1689	0.001	0.0764	0.1085	0.0009	0.0004	0.3165	0.0055

Supplementary Table S28 Statistical analyses (p-values) concerning the *k*-values of the cell proliferation modelling in cells without experimental EMT induction (n.s. = not significant)

k	GK	GK EtOH Ko	GK EtOH	EPI	EPI EtOH Ko	EPI EtOH	FIB	FIB EtOH Ko	FIB EtOH
GK		n.s.	n.s.	n.s.	n.s.	n.s.	n.s.	n.s.	n.s.
GK EtOH Ko	n.s.		n.s.	n.s.	n.s.	n.s.	n.s.	n.s.	n.s.
GK EtOH	n.s.	n.s.		0.0018	0.0029	0.0041	n.s.	n.s.	n.s.
EPI	n.s.	n.s.	0.0018		n.s.	n.s.	0.0022	0.0008	0.0117
EPI EtOH Ko	n.s.	n.s.	0.0029	n.s.		n.s.	0.0035	0.0012	0.0183
EPI EtOH	n.s.	n.s.	0.0041	n.s.	n.s.		0.0049	0.0018	0.0251
FIB	n.s.	n.s.	n.s.	0.0022	0.0035	0.0049		n.s.	n.s.
FIB EtOH Ko	n.s.	n.s.	n.s.	0.0008	0.0012	0.0018	n.s.		n.s.
FIB EtOH	n.s.	n.s.	n.s.	0.0117	0.0183	0.0251	n.s.	n.s.	

Supplementary Table S29 Statistical analyses (p-values) concerning the Y_m-values of the cell proliferation modelling in cells without experimental EMT induction (n.s. = not significant)

Ym	GK	GK EtOH Ko	GK EtOH	EPI	EPI EtOH Ko	EPI EtOH	FIB	FIB EtOH Ko	FIB EtOH
GK		n.s.	n.s.	<0,0001	n.s.	n.s.	n.s.	n.s.	n.s.
GK EtOH Ko	n.s.		n.s.	<0,0001	n.s.	n.s.	n.s.	n.s.	n.s.
GK EtOH	n.s.	n.s.		<0,0001	n.s.	n.s.	n.s.	n.s.	n.s.
EPI	<0,0001	<0,0001	<0,0001		n.s.	0.002	<0,0001	<0,0001	<0,0001
EPI EtOH Ko	n.s.	n.s.	n.s.	n.s.		n.s.	n.s.	n.s.	n.s.
EPI EtOH	n.s.	n.s.	n.s.	0.002	n.s.		n.s.	n.s.	n.s.
FIB	n.s.	n.s.	n.s.	<0,0001	n.s.	n.s.		n.s.	n.s.
FIB EtOH Ko	n.s.	n.s.	n.s.	<0,0001	n.s.	n.s.	n.s.		n.s.
FIB EtOH	n.s.	n.s.	n.s.	<0,0001	n.s.	n.s.	n.s.	n.s.	

Supplementary Table S30 Statistical analyses (p-values) concerning the k- and Y_m-values of the cell proliferation modelling in untreated ("-EMT") and treated ("+EMT") cells

p-value	GK -EMT vs. GK +EMT	GK EtOH Ko -EMT vs. GK EtOH Ko +EMT	GK EtOH -EMT vs. GK EtOH +EMT	EPI -EMT vs. EPI +EMT	EPI EtOH Ko -EMT vs. EPI EtOH Ko +EMT	EPI EtOH -EMT vs. EPI EtOH +EMT	FIB -EMT vs. FIB +EMT	FIB EtOH Ko -EMT vs. FIB EtOH Ko +EMT	FIB EtOH -EMT vs. FIB EtOH +EMT
k	0.6268	0.0748	0.577	0.8117	0.343	0.9697	0.5164	0.5336	0.7566
Ym	0.0034	0.0022	0.0012	0.9761	0.0888	0.2597	0.7255	0.9075	0.1415

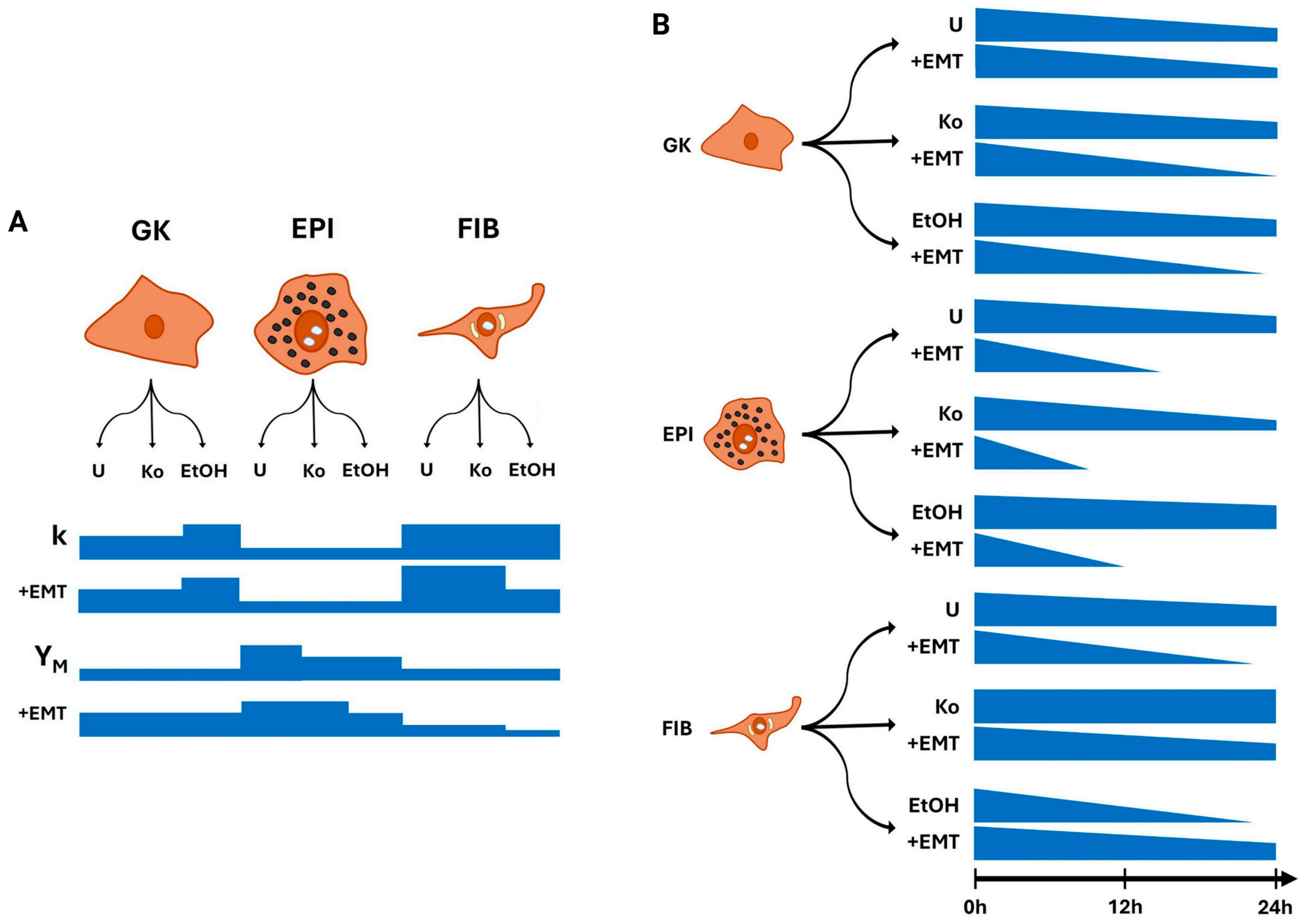

Supplementary Figure S27 Schematic summary of the effects of cell culture conditions, EtOH-treatment and "+EMT"-treatment on cellular proliferation and migration in GK, EPI and FIB cells and their derivatives. U = untreated cell line; EtOH = EtOH-treatment; Ko = control culture condition; +EMT = treated with EMT-inducing medium. For color/symbol legend see Figure 1. A: The upper blue bars indicate the relative cell proliferation as approximated by the parameters *k* and Y_m (details see main text). The lower blue bars represent the direct comparison of cell proliferation in the respective cell line with and without addition of the EMT cocktail. B: The upper blue bars indicate migratory behavior of the respective cell lines as a function of time. The lower blue bars represent the direct comparison of cell migration in the respective cell line with and without addition of the EMT cocktail as a function of time. Created in BioRender. Steinberg, T. (2025) https://BioRender.com/riyp5yj

Supplementary Table S31 Statistical analyses (p-values) concerning the cellular migration in cells without experimental EMT induction at t_0 = 0 h (n.s. = not significant)

0h	GK	GKEtOH Ko	GKEtOH	EPI	EPI EtOH Ko	EPI EtOH	FIB	FIBEtOH Ko	FIBEtOH
GK		n.s.	n.s.	n.s.	n.s.	n.s.	n.s.	n.s.	n.s.
GKEtOH Ko	n.s.		n.s.	n.s.	n.s.	n.s.	n.s.	n.s.	n.s.
GKEtOH	n.s.	n.s.		n.s.	n.s.	n.s.	n.s.	n.s.	n.s.
EPI	n.s.	n.s.	n.s.		n.s.	n.s.	n.s.	n.s.	n.s.
EPI EtOH Ko	n.s.	n.s.	n.s.	n.s.		n.s.	n.s.	n.s.	n.s.
EPI EtOH	n.s.	n.s.	n.s.	n.s.	n.s.		n.s.	n.s.	n.s.
FIB	n.s.	n.s.	n.s.	n.s.	n.s.	n.s.		n.s.	n.s.
FIBEtOH Ko	n.s.	n.s.	n.s.	n.s.	n.s.	n.s.	n.s.		n.s.
FIBEtOH	n.s.	n.s.	n.s.	n.s.	n.s.	n.s.	n.s.	n.s.	

Supplementary Table S32 Statistical analyses (p-values) concerning the cellular migration in cells without experimental EMT induction at t_1 = 1 h (n.s. = not significant)

1h	GK	GKEtOH Ko	GKEtOH	EPI	EPI EtOH Ko	EPI EtOH	FIB	FIBEtOH Ko	FIBEtOH
GK		n.s.	n.s.	n.s.	n.s.	n.s.	n.s.	n.s.	n.s.
GKEtOH Ko	n.s.		n.s.	n.s.	n.s.	n.s.	n.s.	n.s.	n.s.
GKEtOH	n.s.	n.s.		n.s.	n.s.	n.s.	n.s.	n.s.	n.s.
EPI	n.s.	n.s.	n.s.		n.s.	n.s.	n.s.	n.s.	n.s.
EPI EtOH Ko	n.s.	n.s.	n.s.	n.s.		n.s.	n.s.	n.s.	n.s.
EPI EtOH	n.s.	n.s.	n.s.	n.s.	n.s.		n.s.	n.s.	n.s.
FIB	n.s.	n.s.	n.s.	n.s.	n.s.	n.s.		n.s.	n.s.
FIBEtOH Ko	n.s.	n.s.	n.s.	n.s.	n.s.	n.s.	n.s.		n.s.
FIBEtOH	n.s.	n.s.	n.s.	n.s.	n.s.	n.s.	n.s.	n.s.	

Supplementary Table S33 Statistical analyses (p-values) concerning the cellular migration in cells without experimental EMT induction at t_2 = 2 h (n.s. = not significant)

2h	GK	GKEtOH Ko	GKEtOH	EPI	EPI EtOH Ko	EPI EtOH	FIB	FIBEtOH Ko	FIBEtOH
GK		n.s.	n.s.	n.s.	n.s.	n.s.	n.s.	n.s.	n.s.
GKEtOH Ko	n.s.		n.s.	n.s.	n.s.	n.s.	n.s.	n.s.	n.s.
GKEtOH	n.s.	n.s.		n.s.	n.s.	n.s.	n.s.	n.s.	n.s.
EPI	n.s.	n.s.	n.s.		n.s.	n.s.	n.s.	n.s.	n.s.
EPI EtOH Ko	n.s.	n.s.	n.s.	n.s.		n.s.	n.s.	0.0304	n.s.
EPI EtOH	n.s.	n.s.	n.s.	n.s.	n.s.		n.s.	0.003	n.s.
FIB	n.s.	n.s.	n.s.	n.s.	n.s.	n.s.		n.s.	n.s.
FIBEtOH Ko	n.s.	n.s.	n.s.	n.s.	0.0304	0.003	n.s.		0.0283
FIBEtOH	n.s.	n.s.	n.s.	n.s.	n.s.	n.s.	n.s.	0.0283	

Supplementary Table S34 Statistical analyses (p-values) concerning the cellular migration in cells without experimental EMT induction at t_3 = 3 h (n.s. = not significant)

3h	GK	GKEtOH Ko	GKEtOH	EPI	EPI EtOH Ko	EPI EtOH	FIB	FIBEtOH Ko	FIBEtOH
GK		n.s.	n.s.	n.s.	n.s.	n.s.	n.s.	n.s.	n.s.
GKEtOH Ko	n.s.		n.s.	n.s.	n.s.	n.s.	n.s.	n.s.	n.s.
GKEtOH	n.s.	n.s.		n.s.	n.s.	n.s.	n.s.	n.s.	n.s.
EPI	n.s.	n.s.	n.s.		n.s.	n.s.	n.s.	n.s.	n.s.
EPI EtOH Ko	n.s.	n.s.	n.s.	n.s.		n.s.	n.s.	0.042	n.s.
EPI EtOH	n.s.	n.s.	n.s.	n.s.	n.s.		n.s.	0.0192	n.s.
FIB	n.s.	n.s.	n.s.	n.s.	n.s.	n.s.		n.s.	n.s.
FIBEtOH Ko	n.s.	n.s.	n.s.	n.s.	0.042	0.0192	n.s.		n.s.
FIBEtOH	n.s.	n.s.	n.s.	n.s.	n.s.	n.s.	n.s.	n.s.	

Supplementary Table S35 Statistical analyses (p-values) concerning the cellular migration in cells without experimental EMT induction at $t_4 = 4$ h (n.s. = not significant)

4h	GK	GKEtOH Ko	GKEtOH	EPI	EPI EtOH Ko	EPI EtOH	FIB	FIBEtOH Ko	FIBEtOH
GK		n.s.	n.s.	n.s.	n.s.	n.s.	n.s.	n.s.	n.s.
GKEtOH Ko	n.s.		n.s.	n.s.	n.s.	n.s.	n.s.	n.s.	n.s.
GKEtOH	n.s.	n.s.		n.s.	n.s.	n.s.	n.s.	n.s.	n.s.
EPI	n.s.	n.s.	n.s.		n.s.	n.s.	n.s.	n.s.	n.s.
EPI EtOH Ko	n.s.	n.s.	n.s.	n.s.		n.s.	n.s.	0.018	n.s.
EPI EtOH	n.s.	n.s.	n.s.	n.s.	n.s.		n.s.	0.0068	n.s.
FIB	n.s.	n.s.	n.s.	n.s.	n.s.	n.s.		n.s.	n.s.
FIBEtOH Ko	n.s.	n.s.	n.s.	n.s.	0.018	0.0068	n.s.		n.s.
FIBEtOH	n.s.	n.s.	n.s.	n.s.	n.s.	n.s.	n.s.	n.s.	

Supplementary Table S36 Statistical analyses (p-values) concerning the cellular migration in cells without experimental EMT induction at $t_5 = 5$ h (n.s. = not significant)

5h	GK	GKEtOH Ko	GKEtOH	EPI	EPI EtOH Ko	EPI EtOH	FIB	FIBEtOH Ko	FIBEtOH
GK		n.s.	n.s.	n.s.	n.s.	n.s.	n.s.	n.s.	n.s.
GKEtOH Ko	n.s.		n.s.	n.s.	n.s.	n.s.	n.s.	n.s.	n.s.
GKEtOH	n.s.	n.s.		n.s.	n.s.	n.s.	n.s.	n.s.	n.s.
EPI	n.s.	n.s.	n.s.		n.s.	n.s.	n.s.	0.044	n.s.
EPI EtOH Ko	n.s.	n.s.	n.s.	n.s.		n.s.	n.s.	0.0041	n.s.
EPI EtOH	n.s.	n.s.	n.s.	n.s.	n.s.		n.s.	0.0021	n.s.
FIB	n.s.	n.s.	n.s.	n.s.	n.s.	n.s.		0.0298	n.s.
FIBEtOH Ko	n.s.	n.s.	n.s.	0.044	0.0041	0.0021	0.0298		0.0308
FIBEtOH	n.s.	n.s.	n.s.	n.s.	n.s.	n.s.	n.s.	0.0308	

Supplementary Table S37 Statistical analyses (p-values) concerning the cellular migration in cells without experimental EMT induction at $t_6 = 6$ h (n.s. = not significant)

6h	GK	GKEtOH Ko	GKEtOH	EPI	EPI EtOH Ko	EPI EtOH	FIB	FIBEtOH Ko	FIBEtOH
GK		n.s.	n.s.	n.s.	n.s.	n.s.	n.s.	0.007	n.s.
GKEtOH Ko	n.s.		n.s.	n.s.	n.s.	n.s.	n.s.	0.0091	n.s.
GKEtOH	n.s.	n.s.		n.s.	n.s.	n.s.	n.s.	0.0143	n.s.
EPI	n.s.	n.s.	n.s.		n.s.	n.s.	n.s.	0.006	n.s.
EPI EtOH Ko	n.s.	n.s.	n.s.	n.s.		n.s.	n.s.	0.0006	n.s.
EPI EtOH	n.s.	n.s.	n.s.	n.s.	n.s.		n.s.	0.0003	n.s.
FIB	n.s.	n.s.	n.s.	n.s.	n.s.	n.s.		0.0054	n.s.
FIBEtOH Ko	0.007	0.0091	0.0143	0.006	0.0006	0.0003	0.0054		0.0008
FIBEtOH	n.s.	n.s.	n.s.	n.s.	n.s.	n.s.	n.s.	0.0008	

Supplementary Table S38 Statistical analyses (p-values) concerning the cellular migration in cells without experimental EMT induction at $t_7 = 8$ h (n.s. = not significant)

8h	GK	GKEtOH Ko	GKEtOH	EPI	EPI EtOH Ko	EPI EtOH	FIB	FIBEtOH Ko	FIBEtOH
**GK**		n.s.	n.s.	n.s.	n.s.	n.s.	n.s.	n.s.	n.s.
**GKEtOH Ko**	n.s.		n.s.	n.s.	n.s.	n.s.	n.s.	n.s.	n.s.
**GKEtOH**	n.s.	n.s.		n.s.	n.s.	n.s.	n.s.	n.s.	n.s.
**EPI**	n.s.	n.s.	n.s.		n.s.	n.s.	n.s.	n.s.	n.s.
**EPI EtOH Ko**	n.s.	n.s.	n.s.	n.s.		n.s.	n.s.	0.0258	n.s.
**EPI EtOH**	n.s.	n.s.	n.s.	n.s.	n.s.		n.s.	0.005	n.s.
**FIB**	n.s.	n.s.	n.s.	n.s.	n.s.	n.s.		n.s.	n.s.
**FIBEtOH Ko**	n.s.	n.s.	n.s.	n.s.	0.0258	0.005	n.s.		0.0213
**FIBEtOH**	n.s.	n.s.	n.s.	n.s.	n.s.	n.s.	n.s.	0.0213	

Supplementary Table S39 Statistical analyses (p-values) concerning the cellular migration in cells without experimental EMT induction at t_8 = 10 h (n.s. = not significant)

10h	GK	GKEtOH Ko	GKEtOH	EPI	EPI EtOH Ko	EPI EtOH	FIB	FIBEtOH Ko	FIBEtOH
**GK**		n.s.	n.s.	n.s.	n.s.	n.s.	n.s.	0.0033	n.s.
**GKEtOH Ko**	n.s.		n.s.	n.s.	n.s.	n.s.	n.s.	0.0027	n.s.
**GKEtOH**	n.s.	n.s.		n.s.	n.s.	n.s.	n.s.	0.0057	n.s.
**EPI**	n.s.	n.s.	n.s.		n.s.	n.s.	n.s.	0.0029	n.s.
**EPI EtOH Ko**	n.s.	n.s.	n.s.	n.s.		n.s.	n.s.	0.0004	n.s.
**EPI EtOH**	n.s.	n.s.	n.s.	n.s.	n.s.		n.s.	<0,0001	n.s.
**FIB**	n.s.	n.s.	n.s.	n.s.	n.s.	n.s.		0.0023	n.s.
**FIBEtOH Ko**	0.0033	0.0027	0.0057	0.0029	0.0004	<0,0001	0.0023		0.0015
**FIBEtOH**	n.s.	n.s.	n.s.	n.s.	n.s.	n.s.	n.s.	0.0015	

Supplementary Table S40 Statistical analyses (p-values) concerning the cellular migration in cells without experimental EMT induction at t_9 = 12 h (n.s. = not significant)

12h	GK	GKEtOH Ko	GKEtOH	EPI	EPI EtOH Ko	EPI EtOH	FIB	FIBEtOH Ko	FIBEtOH
**GK**		n.s.	n.s.	n.s.	n.s.	n.s.	n.s.		n.s.
**GKEtOH Ko**	n.s.		n.s.	n.s.	n.s.	n.s.	n.s.		n.s.
**GKEtOH**	n.s.	n.s.		n.s.	n.s.	n.s.	n.s.		n.s.
**EPI**	n.s.	n.s.	n.s.		n.s.	n.s.	n.s.		n.s.
**EPI EtOH Ko**	n.s.	n.s.	n.s.	n.s.		n.s.	n.s.		n.s.
**EPI EtOH**	n.s.	n.s.	n.s.	n.s.	n.s.		n.s.		n.s.
**FIB**	n.s.	n.s.	n.s.	n.s.	n.s.	n.s.			n.s.
**FIBEtOH Ko**									
**FIBEtOH**	n.s.	n.s.	n.s.	n.s.	n.s.	n.s.	n.s.		

Supplementary Table S41 Statistical analyses (p-values) concerning the cellular migration in cells without experimental EMT induction at t_{10} = 16 h (n.s. = not significant)

16h	GK	GKEtOH Ko	GKEtOH	EPI	EPI EtOH Ko	EPI EtOH	FIB	FIBEtOH Ko	FIBEtOH
**GK**		n.s.	n.s.	n.s.	n.s.	n.s.	n.s.		n.s.
**GKEtOH Ko**	n.s.		n.s.	n.s.	n.s.	n.s.	n.s.		n.s.
**GKEtOH**	n.s.	n.s.		n.s.	n.s.	n.s.	n.s.		n.s.
**EPI**	n.s.	n.s.	n.s.		n.s.	n.s.	n.s.		n.s.
**EPI EtOH Ko**	n.s.	n.s.	n.s.	n.s.		n.s.	n.s.		n.s.
**EPI EtOH**	n.s.	n.s.	n.s.	n.s.	n.s.		n.s.		n.s.
**FIB**	n.s.	n.s.	n.s.	n.s.	n.s.	n.s.			n.s.
**FIBEtOH Ko**									
**FIBEtOH**	n.s.	n.s.	n.s.	n.s.	n.s.	n.s.	n.s.		

Supplementary Table S42 Statistical analyses (p-values) concerning the cellular migration in cells without experimental EMT induction at t_{11} = 20 h (n.s. = not significant)

20h	GK	GK EtOH Ko	GK EtOH	EPI	EPI EtOH Ko	EPI EtOH	FIB	FIB EtOH Ko	FIB EtOH
GK		n.s.	n.s.	n.s.	n.s.	n.s.	n.s.		n.s.
GK EtOH Ko	n.s.		n.s.	n.s.	n.s.	n.s.	n.s.		n.s.
GK EtOH	n.s.	n.s.		n.s.	n.s.	n.s.	n.s.		n.s.
EPI	n.s.	n.s.	n.s.		n.s.	n.s.	n.s.		n.s.
EPI EtOH Ko	n.s.	n.s.	n.s.	n.s.		n.s.	n.s.		n.s.
EPI EtOH	n.s.	n.s.	n.s.	n.s.	n.s.		n.s.		n.s.
FIB	n.s.	n.s.	n.s.	n.s.	n.s.	n.s.			n.s.
FIB EtOH Ko									
FIB EtOH	n.s.	n.s.	n.s.	n.s.	n.s.	n.s.	n.s.		

Supplementary Table S43 Statistical analyses (p-values) concerning the cellular migration in cells without experimental EMT induction at t_{12} = 24 h (n.s. = not significant)

24h	GK	GK EtOH Ko	GK EtOH	EPI	EPI EtOH Ko	EPI EtOH	FIB	FIB EtOH Ko	FIB EtOH
GK		n.s.	n.s.	n.s.	n.s.	n.s.	n.s.	0.0086	n.s.
GK EtOH Ko	n.s.		n.s.	n.s.	n.s.	n.s.	n.s.	0.0021	n.s.
GK EtOH	n.s.	n.s.		n.s.	n.s.	n.s.	n.s.	0.0466	n.s.
EPI	n.s.	n.s.	n.s.		n.s.	n.s.	n.s.	0.0405	n.s.
EPI EtOH Ko	n.s.	n.s.	n.s.	n.s.		n.s.	n.s.	0.0109	n.s.
EPI EtOH	n.s.	n.s.	n.s.	n.s.	n.s.		n.s.	0.0026	n.s.
FIB	n.s.	n.s.	n.s.	n.s.	n.s.	n.s.		0.0145	n.s.
FIB EtOH Ko	0.0086	0.0021	0.0466	0.0405	0.0109	0.0026	0.0145		0.0054
FIB EtOH	n.s.	n.s.	n.s.	n.s.	n.s.	n.s.	n.s.	0.0054	

Supplementary Table S44 Statistical analyses (p-values) concerning the cellular migration in untreated ("-EMT") and treated ("+EMT") cells at different points in time

p-value	GK -EMT vs. GK +EMT	GK EtOH Ko -EMT vs. GK EtOH Ko +EMT	GK EtOH -EMT vs. GK EtOH +EMT	EPI -EMT vs. EPI +EMT	EPI EtOH Ko -EMT vs. EPI EtOH Ko +EMT	EPI EtOH -EMT vs. EPI EtOH +EMT	FIB -EMT vs. FIB +EMT	FIB EtOH Ko -EMT vs. FIB EtOH Ko +EMT	FIB EtOH -EMT vs. FIB EtOH +EMT
0h	0.648	0.1809	0.0137	0.1925	0.725	0.287	0.0154	0.0458	0.4274
1h	0.9626	0.012	0.0684	0.0871	0.0296	0.1869	0.4339	0.7089	0.3036
2h	0.985	0.1449	0.1184	0.005	0.0002	0.0225	0.0473	0.2878	0.4721
3h	0.3571	0.0891	0.0265	0.0158	0.0362	0.0014	0.2323	0.1362	0.1278
4h	0.3756	0.5316	0.0105	0.1619	0.0331	0.0006	0.0069	0.2791	0.0062
5h	0.205	0.992	0.0558	<0,0001	0.0876	0.0001	0.1835	0.1852	0.3679
6h	0.5209	0.2243	0.0016	0.0065	0.1269	0.0022	0.1568	0.0798	0.0277
8h	0.2537	0.7329	0.0614	0.0068	0.1389	<0,0001	0.031	0.0381	0.0105
10h	0.3899	0.3505	0.0085	0.0102	0.0762	0.0002	0.0585	0.1972	0.017
12h	0.1877	0.0148	0.1151	0.0116	0.3002	<0,0001	0.0023	0.0596	0.0065
16h	0.2296	0.0078	0.0045	0.0568	0.2958	<0,0001	0.0015	0.1823	0.0092
20h	0.3032	0.0224	0.0198	0.0112	0.2713	<0,0001	<0,0001	0.1078	0.0007
24h	0.7244	0.0448	0.0658	0.0991	0.3739	<0,0001	<0,0001	0.086	0.0002

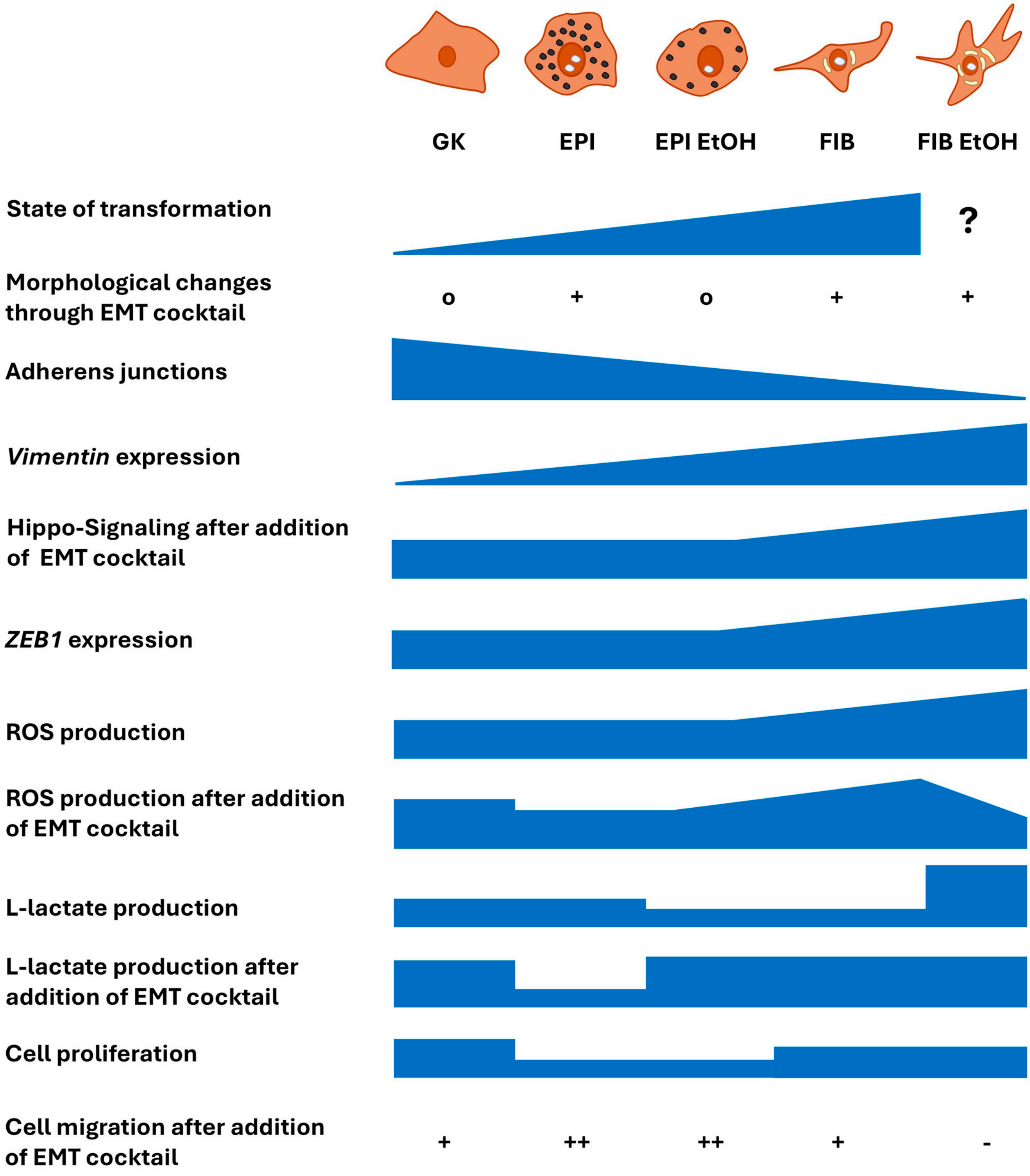

Supplementary Figure S28 Schematic synopsis of the main findings of this work. The graphic summarizes the differentiating properties of the cell lines in various domains such as cell morphology, gene expression and cell behavior. The combination of these characteristics defines the state of transformation (see main text). For color/symbol legend see Figure 1. The height of the blue bars is proportional to the magnitude of the respective property. O = no influence; + = slight positive influence; ++ = strong positive influence: - = negative influence. Created in BioRender. Steinberg, T. (2025) https://BioRender.com/v4bvvjp
